# Validation of 46 loci associated with female fertility traits in cattle

**DOI:** 10.1186/s12864-019-5935-3

**Published:** 2019-07-12

**Authors:** Jennifer N. Kiser, Elizabeth M. Keuter, Christopher M. Seabury, Mahesh Neupane, Joao G. N. Moraes, Joseph Dalton, Gregory W. Burns, Thomas E. Spencer, Holly L. Neibergs

**Affiliations:** 1 Department of Animal Sciences and Center for Reproductive Biology, Washington State University, Pullman, WA USA; 2 Department of Veterinary Pathobiology, College of Veterinary Medicine, Texas A&M University, College Station, TX USA; 3 Division of Animal Sciences, University of Missouri, Columbia, MO USA; 4 Department of Animal and Veterinary Sciences, University of Idaho, Caldwell, ID USA

**Keywords:** Cattle, Conception rate, Dairy, Loci, Fertility

## Abstract

**Background:**

Subfertility is one challenge facing the dairy industry as the average Holstein heifer conception rate (HCR), the proportion of heifers that conceive and maintain a pregnancy per breeding, is estimated at 55–60%. Of the loci associated with HCR, few have been validated in an independent cattle population, limiting their usefulness for selection or furthering our understanding of the mechanisms involved in successful pregnancy. Therefore, the objectives here were to identify loci associated with HCR: 1) to the first artificial insemination (AI) service (HCR1), 2) to repeated AI services required for a heifer to conceive (TBRD) and 3) to validate loci previously associated with fertility*.* Breeding and health records from 3359 Holstein heifers were obtained after heifers were bred by AI at observed estrus, with pregnancy determined at day 35 via palpation. Heifer DNA was genotyped using the Illumina BovineHD BeadChip, and genome-wide association analyses (GWAA) were performed with additive, dominant and recessive models using the Efficient Mixed Model Association eXpedited (EMMAX) method with a relationship matrix for two phenotypes. The HCR1 GWAA compared heifers that were pregnant after the first AI service (*n* = 497) to heifers that were open following the first AI service (*n* = 405), which included those that never conceived. The TBRD GWAA compared only those heifers which did conceive, across variable numbers of AI service (*n* = 712). Comparison of loci previously associated with fertility, HCR1 or TBRD were considered the same locus for validation when in linkage disequilibrium (D’ > 0.7).

**Results:**

The HCR1 GWAA identified 116, 187 and 28 loci associated (*P* < 5 × 10^− 8^) in additive, dominant and recessive models, respectively. The TBRD GWAA identified 235, 362, and 69 QTL associated (*P* < 5 × 10^− 8^) with additive, dominant and recessive models, respectively. Loci previously associated with fertility were in linkage disequilibrium with 22 loci shared with HCR1 and TBRD, 5 HCR1 and 19 TBRD loci.

**Conclusions:**

Loci associated with HCR1 and TBRD that have been identified and validated can be used to improve HCR through genomic selection, and to better understand possible mechanisms associated with subfertility.

**Electronic supplementary material:**

The online version of this article (10.1186/s12864-019-5935-3) contains supplementary material, which is available to authorized users.

## Background

Despite recent upward trends for heifer fertility, conception rates remain an area of concern for dairy producers, with heifer conception rate (HCR), or the percentage of heifers that conceive following a breeding, averaging between 55 and 60% industry-wide [1, 2]. Fertility problems are one of the most frequent reasons for culling, and poor conception rates can affect profitability and sustainability of dairy production [3]. Pregnancy rate and conception rate are two commonly measured, complex traits in dairy cattle with previous heritability estimates ranging from 1 to 4% [4–10]. Direct selection for female fertility was initiated in the U.S. in 2003 with the introduction of genetic evaluation for daughter pregnancy rate, and its inclusion as a component of selection indices [11]. Daughter pregnancy rate is defined as the percentage of a bull’s daughters who become pregnant in a 21-day period without considering the number of services required, or whether the females were exposed to a breeding [12]. In 2014, HCR was included in selection indices, as HCR provides a more quantitative assessment of the ability of a heifer to successfully conceive and maintain a pregnancy [13]. Selection for HCR has been moderately effective, as evidenced by a 6% increase in HCR since 2010 [14–18]. However, the genetic mechanisms impacting HCR are unknown.

Genome-wide association analysis (GWAA) can potentially enhance genomic selection by identifying loci strongly associated with HCR, and by providing a more complete understanding of the genes underlying differential conception rate as well as early pregnancy establishment. In recent years, several GWAA have been conducted to identify regions strongly associated with both heifer and cow fertility traits. However, to date, few loci have been identified by independent populations or across breeds [19, 20]. Therefore, the objective of this study was to identify loci associated with HCR using GWAA in U.S. Holstein heifers and determine if any previously identified loci were validated through these HCR associations.

## Methods

### Study population

This study was conducted with approval from the Institutional Animal Care and Use Committee at Washington State University (4295). Holstein heifers (*n* = 3359) from a commercial heifer raising facility in Parma, Idaho were artificially inseminated (AI) to determine HCR. Heifers were bred following observed estrus after reaching approximately 11–13 months of age, a minimum height of 129 cm at the withers, and a minimum weight of 390 kg. Heifers were bred via AI with semen representing 51 different Holstein sires. Pregnancy was determined via rectal palpation of the uterus 35 days after AI, and DairyComp305 (Valley Agricultural Software, Tulare, CA) health records were used to eliminate heifers that subsequently aborted their calf. After phenotypic evaluation of 3359 heifers, 497 heifers that conceived via the first AI service, 215 heifers that conceived on or after the 4th AI service, and 190 heifers that never conceived despite at least 4 AI services were selected for enrollment in the present study.

### Phenotypes

Pregnancy success after first AI service was used as the first phenotype. The HCR to first service (HCR1) compared 497 heifers that were pregnant following the first AI service to 405 heifers that did not conceive following the first service. The second phenotype of interest, which reflects the number of times a heifer was bred to achieve a pregnancy (TBRD) with semen from one or more sires, compared 497 heifers that were pregnant following the first AI service (coded as 1) to 215 heifers that only achieved pregnancy following the fourth (*n* = 208) and fifth (*n* = 7) AI service (coded as 4, and 5, respectively). The conception rate between AI sires was not significant (*P* = 0.07) so failure to conceive was not due to service sire infertility. None of the AI services utilized semen that was sorted by sex which could have influenced conception rate. In addition, heifers that did not conceive at the first service were typically bred to different sires before they were culled which further limited the potential of heifers to fail to conceive due to repeatedly being bred to a sire with poor fertility. Heifers achieving pregnancy success at the fourth and fifth AI service remained as replacements although they were less desirable due to the additional cost incurred prior to their first lactation. Notably, 159 heifers that failed to conceive at the fourth AI attempt were culled prior to the fifth AI service. As these heifers never conceived in this study, they were removed from the TBRD analysis but were included in the HCR1 analysis. Only 23 heifers that failed to conceive after the fourth AI attempt were bred a fifth time. This comparison of first service conception to those heifers requiring 4 or 5 services to maintain a pregnancy to day 35 was used to represent extreme phenotypic differences in heifer fertility and to increase statistical power.

Evaluation of pregnancy status at day 35 after breeding measures the outcome of several physiological processes including ovulation, fertilization, blastocyst formation with growth into an elongated conceptus, pregnancy recognition signaling, and development of the embryo as well as the chorioallantoic placenta. Therefore, the HCR1 and TBRD phenotypes represent a complex phenotype where many factors have a role in a heifer being pregnant or open at day 35 after breeding. The identification of loci associated with HCR1 and TBRD, and their functional relationships with these specific factors, will assist in further understanding the genetic mechanisms important for pregnancy establishment in Holstein dairy cattle.

### DNA extraction and genotyping

Approximately 16 ml of whole blood was collected in EDTA tubes via tail vein venipuncture. DNA was subsequently extracted from white blood cell pellets using the Puregene DNA extraction kit (Gentra, Minneapolis, MN) as per manufacturer’s directions. DNA was quantified using the Nanodrop 1000 spectrophotometer (Thermo Fisher Scientific, Wilmington, DE) and genotyped with the Illumina (San Diego, CA) BovineHD BeadChip at Neogen Laboratories (Lincoln, NE). The Illumina BovineHD BeadChip contains 778,962 single nucleotide polymorphisms (SNPs) with an average and median distance between SNPs of 3.43 kb and 2.68 kb, respectively [21].

### Quality control

Animals were removed from the study when > 10% of BovineHD BeadChip genotypes failed (*n* = 49) or if there were inconsistencies in their breeding records (*n* = 14). This left 468 heifers that conceived on the first AI, 203 heifers that conceived on or after the fourth AI, and 168 heifers that never conceived. SNPs were removed when > 10% of the genotypes failed (*n* = 52,507), the minor allele frequency was < 1% (*n* = 134,405), and/or if SNPs failed Hardy-Weinberg Equilibrium testing (*P* < 1 × 10^− 25^, *n* = 497). After quality control, a total of 590,553 SNPs remained for the GWAA.

### Genome-wide association analysis

Two GWAA were performed for this study. The first investigated loci associated with HCR1, and the second investigated loci associated with TBRD. The GWAA were performed using an efficient mixed-model association eXpedited approach with a relationship matrix (EMMAX) using additive, dominant and recessive models as incorporated within the SNP and Variation Suite (SVS) software version 9.1 (Golden Helix, Bozeman, MT) [22]. The additive model was designed to identify associations which depend on the value of alleles having additive effects; where having two minor alleles (aa) is twice as likely to effect the phenotype (conception) as having no minor alleles (AA), and half as likely when only one minor allele (Aa) is present. In the dominant model the test of association was whether having at least one minor (Aa or aa) allele was associated with conception when compared to the homozygous genotype (AA) that had no minor allele. The recessive model tested whether there was an association with conception when two minor alleles (aa) were present compared to having one minor allele (Aa) or no minor alleles (AA). The general mixed model was described as ***y*** = **Xβ** ***+ Z****u* + ϵ, where **y** explains the *n* × 1 vector of observed phenotypes, **X** was an *n* × *f* matrix of fixed effects (*f*), **β** was an *f* × 1 vector containing the fixed effect coefficients, and **Z** was an *n* × *t* matrix relating the random effects (*t*) to the phenotype, and *u* was the random effect of the mixed model [23]. The model assumes residuals to be independent with an identical distribution such that *Var*(*u*) = *σ*_*g*_^2^***K*** and (*ϵ*) = *σ*_*e*_^2^***I***, and *Var*(*y*) = *σ*_*g*_^2^***ZKZ***^′^ + *σ*_*e*_^2^***I*.** For this study **K** was a matrix of pairwise genomic relationships and **Z** was the identity matrix, **I** [23]. Pseudo-heritability was estimated using the equation ${h}^2={\upsigma}_g^2/\left({\upsigma}_g^2+{\upsigma}_e^2\right)$ in SVS, where ${\upsigma}_g^2$ was the genetic variance and ${\upsigma}_e^2$ was the environmental (error) variance [24]. SVS can potentially overestimate heritability and standard error when performing EMMAX if the population size is small. Given this possibility, a second analysis, AI-REML, was also conducted in SVS to estimate heritability in conjunction with genomic best linear unbiased prediction (GBLUP), which utilizes a genomic relationship matrix of allele substitution effects by marker [25, 26] and an average information algorithm to estimate the variance components [27, 28]. Further documentation on methods applied in SVS are detailed at: (http://doc.goldenhelix.com/SVS/latest/svsmanual/mixedModelMethods/overview.html) [22].

Identification of loci associated with HCR1 and TBRD was based on a genome-wide threshold for unadjusted *P-*values of *P* < 5.0 × 10^− 8^ as recommended by the International HapMap Consortium [29, 30]. Positional candidate genes were identified within 17 kb regions surrounding SNPs associated with HCR and TBRD. The size of the positional candidate region surrounding the associated SNPs was based on the average (17 kb) haplotype block size of Holstein heifers using the criteria proposed by Gabriel et al. [31] and carried out in SVS. Loci locations were provided based on UMD 3.1 (ftp://ftp.cbcb.umd.edu/pub/data/Bos_taurus/) and ARS-UCD 1.2 (https://www.animalgenome.org/repository/cattle/UMC_bovine_coordinates/) to facilitate comparisons between previous studies with UMD 3.1 coordinates and the newly published assembly [32].

Validation of associated loci is needed from independent cattle populations for loci to be used in genomic selection or in functional studies to tease out the mechanisms of fertility. Therefore, loci associated with HCR1 and TBRD were compared to 22 previous studies investigating both dairy and beef cattle fertility. These studies investigated a variety of fertility traits, including: days to first service, non-return rate and calving interval [19, 33], pregnancy success at day 28 [34] or day 42 [35], calving to first service [36], conception rates in heifers and in cows [37–39], and postpartum anestrous interval [40]. Loci locations were provided based on UMD 3.1 (ftp://ftp.cbcb.umd.edu/pub/data/Bos_taurus/) and ARS-UCD 1.2 (https://www.animalgenome.org/repository/cattle/UMC_bovine_coordinates/). Loci were determined to be the same when associated SNPs from two different populations were in linkage disequilibrium (D’ > 0.7) [41].

### Network analysis

To identify the interaction of positional candidate genes identified in the GWAA, Ingenuity Pathways Analysis (IPA, Ingenuity® Systems, https://www.ingenuity.com) was conducted and canonical pathways were identified using a Fisher’s exact test with a Benjamini-Hochberg multiple testing correction (*P* < 0.01). The canonical pathways identify gene networks where positional candidate genes are present and interact with one another. Master and upstream regulators were identified using a network bias corrected significance value (*P* < 0.01) that removed the bias for hub genes. The upstream regulator analysis identifies molecules that directly regulate genes. The analysis of master regulators identifies molecules that have indirect relationships to positional candidate genes through upstream regulators. Of the positional candidate genes identified in the GWAA, 296 genes were mapped to human, mouse or rat gene data sets curated by IPA, and were used for the identification of canonical pathways, upstream and master regulators.

### Results and discussion

**Fig. 1:**
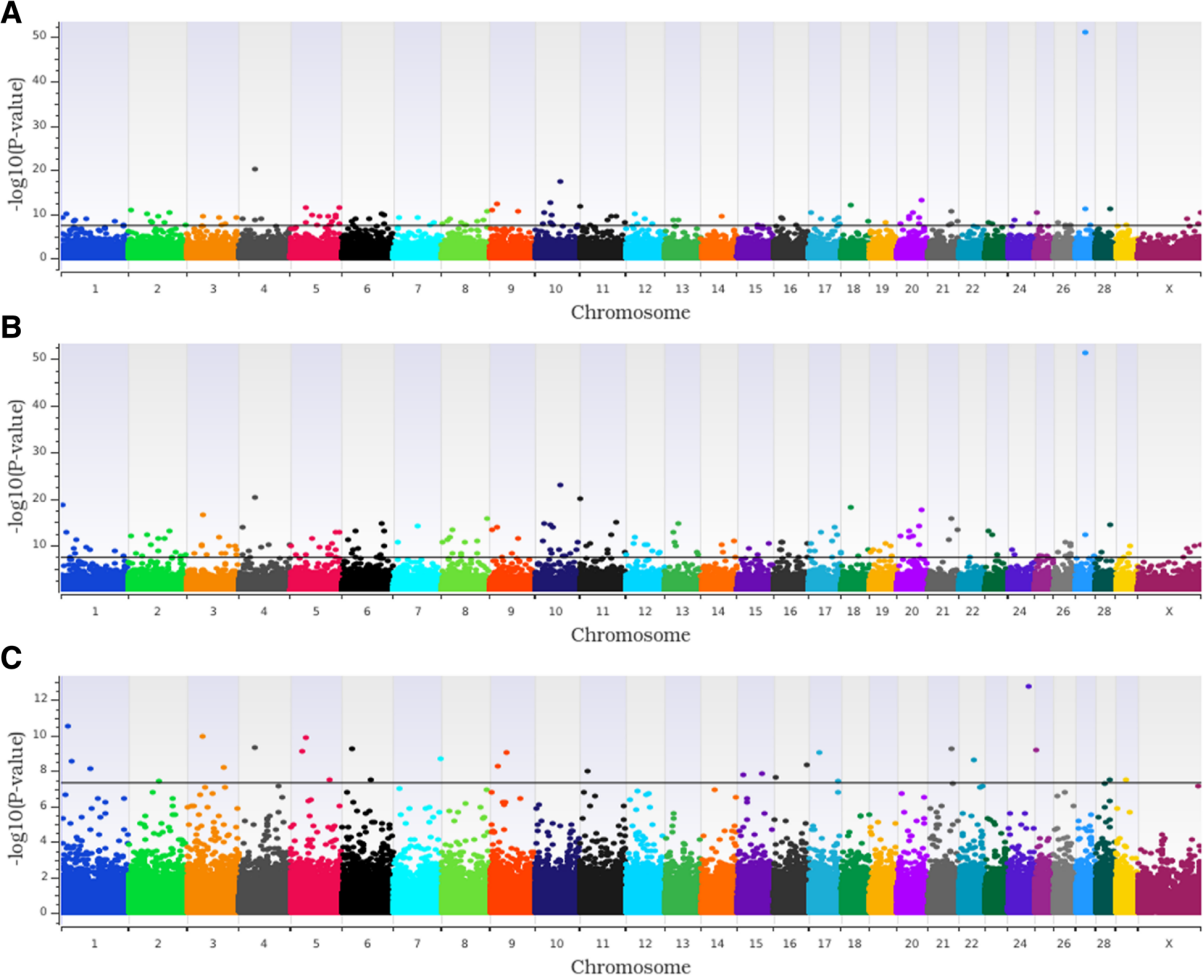
Additive (**a**), dominant (**b**), and recessive (**c**) Manhattan plots for first service conception rate. Single nucleotide polymorphisms are represented by a single dot. Negative log^10^ (*P*-values) ≥ 7.3 (black line) on the y-axis provided evidence for association (*P* < 5.0 × 10^−8^) [25, 26]. Bovine chromosomes are listed on the x-axis

**Fig. 2:**
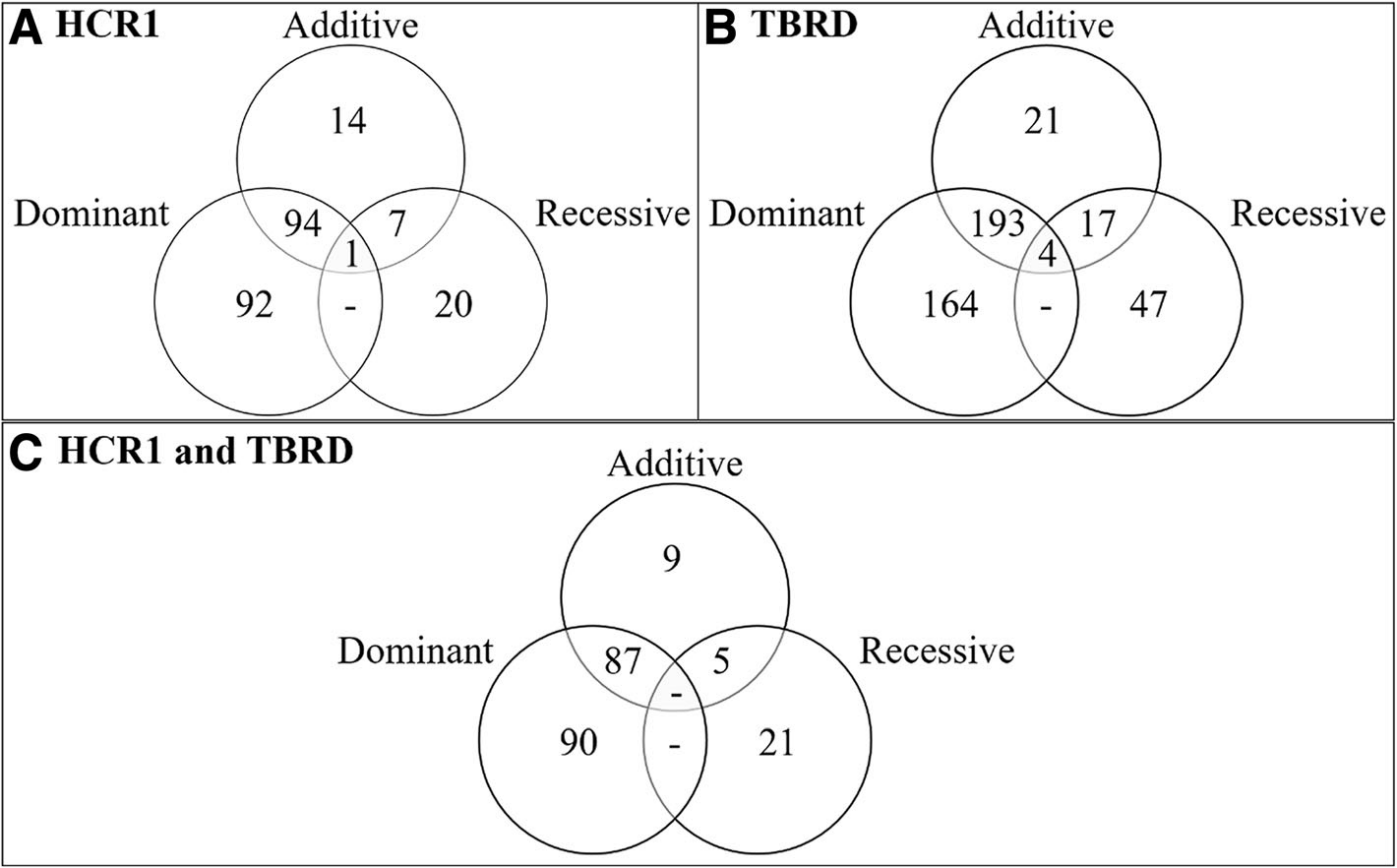
Relationships of loci associated across genotypic models and between phenotypes. Comparison of loci associated with heifer conception rate at first service (HCR1; Panel **a**), number of services to conception (TBRD; Panel **b**) and between HCR1 and TBRD (Panel **c**) across the three (additive, dominant, and recessive) genotypic models

The additive GWAA model identified 116 loci associated (*P* < 5.0 × 10^− 8^) with HCR1, whereas the dominant and recessive models identified 187 and 28 loci, respectively (Figs. 1 and 2a; Additional file 1: Table S1). All but nine of the loci shared between the additive and dominant models (Fig. 2a) were more significant in the dominant model, including four loci that had equivalent *P*-values in both, suggesting that these dominant loci exhibited some additive effects. One of the loci located on *Bos taurus* chromosome (BTA) 4 was associated (*P* < 5.0 × 10^− 8^) with HCR1 across all (additive, dominant and recessive) models (Table 1).

**Table 1:** Top five loci associated with heifer conception rate at first service (HCR1)

BTA^a^	BP Position^b^	SNP ID^c^	Model^d^	*P*-value^e^
1	3,546,098 (4,253,950)	*rs136767715*	Dominant	3.14 × 10^−19^
1	14,740,447 (15,273,288)	*rs109845303*	Recessive	3.31 × 10^−11^
3	37,781,561 (37,660,099)	*rs137663076*	Recessive	1.10 × 10^−10^
4	37,235,676 (37,056,340), 37,618,902 (37,439,721)	*rs133417267, rs43388620*	Additive, Dominant, Recessive	6.93 × 10^− 21^, 6.93 × 10^− 21^, 5.18 × 10^− 09^
5	35,925,646 (35,745,274)	*rs135774967*	Recessive	1.39 × 10^− 10^
10	37,205,266 (37,088,125)	*rs135421839*	Additive	3.15 × 10^− 13^
10	59,882,200 (59,695,958)	*rs110617366*	Additive, Dominant	7.12 × 10^−18^, 1.34 × 10^− 23^
11	2,125,437 (2,159,226)	*rs133812771*	Dominant	1.34 × 10^− 20^
20	57,113,135 (57,038,642)	*rs41956232*	Additive	1.15 × 10^−13^
24	50,503,943 (50,038,623)	*rs110770205*	Recessive	1.74 × 10^−13^
27	21,375,791 (22,307,598)	*rs132728892*	Additive, Dominant	8.35 × 10^−52^, 8.35 × 10^− 52^

^a^ Chromosome location of the locus

^b^ Single nucleotide polymorphism (SNP) location as measured by numbered nucleotides in reference to the UMD 3.1 genome assembly (http://bovinegenome.org/?q=node/61; accessed 15 September 2016) or the ARS-UCD 1.2 genome assembly (https://www.animalgenome.org/repository/cattle/UMC_bovine_coordinates/; accessed 19 September 2018) in parentheses

^c^ The most significant SNP in the locus associated with heifer conception rate as identified by *rs* number which is a reference number assigned to markers submitted to the National Center for Biotechnology Information SNP database (https://www.ncbi.nlm.nih.gov/projects/SNP/; accessed 9 March 2016)

^d^ Genome-wide association model

^e^ Significance (*P-*value) of the most significant SNP associated with heifer conception rate

**Fig. 3:**
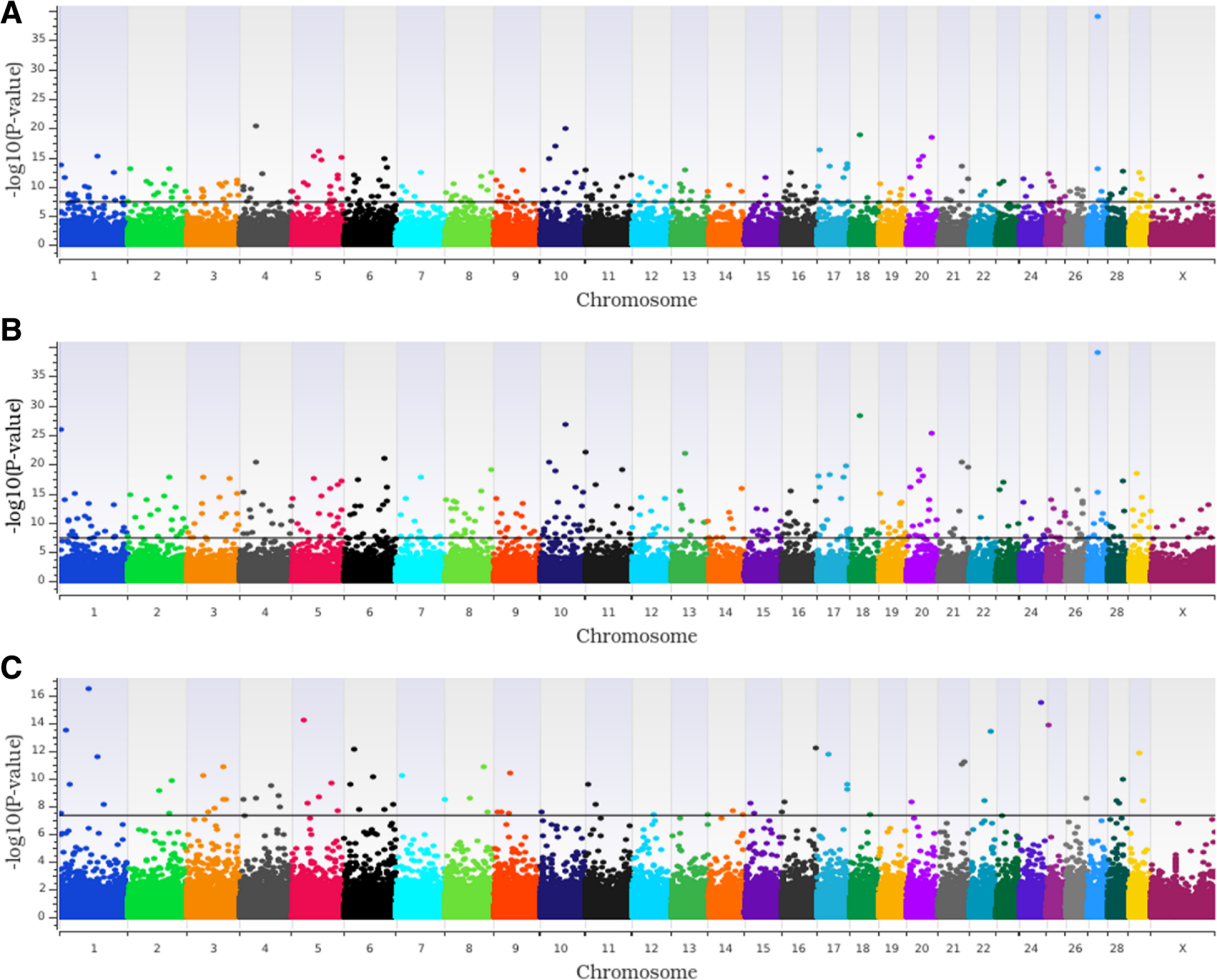
Additive (**a**), dominant (**b**), and recessive (**c**) Manhattan plots for the number of breedings required for a successful pregnancy. Single nucleotide polymorphisms are represented by a single dot. Negative log^10^ (*P*-values) ≥ 7.3 (black line) on the y-axis provided evidence for association (*P* < 5.0 × 10^− 8^) [24, 25]. Bovine chromosomes are listed on the x-axis

For the TBRD GWAA, the additive, dominant and recessive models identified 235, 362 and 69 loci associated (*P* < 5.0 × 10^− 8^) with TBRD, respectively (Figs. 2b and 3; Additional file 1: Table S2). Four loci that had strong associations in the additive model, (BTA1 at 88 Mb, BTA4 at 37 Mb, BTA5 at 62 Mb and BTA17 at 68 Mb) were associated with TBRD in all three models (Fig. 2b; Table 2; Additional file 1: Table S2).

**Table 2:** Top five loci associated with heifer conception rate to repeated AI services (TBRD)

BTA^a^	BP Position^b^	SNP ID^c^	Model^d^	*P*-value^e^
1	3,546,098 (4,253,950)	*rs136767715*	Dominant	1.56 × 10^− 26^
1	14,740,447 (15,273,288)	*rs109845303*	Recessive	3.55 × 10^−14^
1	69,793,194 (69,188,246)	*rs137088827*	Recessive	3.57 × 10^−17^
4	37,235,676 (37,056,340)	*rs133417267*	Additive	4.23 × 10^− 21^
5	27,837,406 (27,683,283)	*rs134147281*	Recessive	6.66 × 10^−15^
10	59,882,200 (59,695,958)	*rs110617366*	Additive, Dominant	1.17 × 10^−20^, 1.57 × 10^−27^
18	21,929,721 (21,851,964)	*rs41872094*	Additive, Dominant	2.15 × 10^−19^, 6.27 × 10^−29^
20	57,113,135 (57,038,642)	*rs41956232*	Additive, Dominant	3.76 × 10^−19^, 4.81 × 10^−26^
24	50,503,943 (50,038,623)	*rs110770205*	Recessive	3.46 × 10^−16^
25	4,169,181 (4,151,257)	*rs110089590*	Recessive	1.76 × 10^−14^
27	21,375,791 (22,307,598)	*rs132728892*	Additive, Dominant	9.10 × 10^−40^, 9.10 × 10^− 40^

^a^ Chromosome location of the locus

^b^ Single nucleotide polymorphism (SNP) location as measured by numbered nucleotides in reference to the UMD 3.1 genome assembly (http://bovinegenome.org/?q=node/61; accessed 15 September 2016) or the ARS-UCD 1.2 genome assembly (https://www.animalgenome.org/repository/cattle/UMC_bovine_coordinates/; accessed 19 September 2018) in parentheses

^c^ The most significant SNP in the locus associated with heifer conception rate as identified by *rs* number which is a reference number assigned to markers submitted to the National Center for Biotechnology Information SNP database (https://www.ncbi.nlm.nih.gov/projects/SNP/; accessed 9 March 2016)

^d^ Genome-wide association model

^e^ Significance (*P-*value) of the most significant SNP associated with heifer conception rate

The loci associated with HCR1 and TBRD were compared to determine whether loci were shared between both phenotypes (Table 3; Fig. 2c). When the top five loci identified in HCR1 and TBRD were compared in the additive and dominant models, four loci on BTA4, BTA10, BTA20 and BTA27 were shared (Table 3). Additionally, the locus on BTA4 was shared across all phenotypes and models (Fig. 2c).

**Table 3:** Top additive and dominant loci across conception rate at first service and to repeated AI services

BTA^a^	BP Position^b^	SNP ID^c^	Model^d^	*P*-value^e^		Positional Candidate Gene
				HCR1	TBRD	
4	37,235,676 (37,056,340)	*rs133417267*	Additive, Dominant	6.93 × 10^−21^, 6.93 × 10^− 21^	4.23 × 10^− 21^, 4.23 × 10^− 21^	*–*
10	59,882,200 (59,695,958)	*rs110617366*	Additive, Dominant	7.12 × 10^−18^, 1.34 × 10^− 23^	1.17 × 10^− 20^, 1.57 × 10^− 27^	*TRPM7*
20	57,113,135 (57,038,642)	*rs41956232*	Additive, Dominant	1.15 × 10^−13^, 3.03 × 10^− 18^	3.76 × 10^− 19^, 4.81 × 10^− 26^	*MARCH11*
27	21,375,791 (22,307,598)	*rs132728892*	Additive, Dominant	8.35 × 10^− 52^, 8.35 × 10^− 52^	9.10 × 10^− 40^, 9.10 × 10^− 40^	*MIR383*

^a^ Chromosome location of the locus

^b^ Single nucleotide polymorphism (SNP) location as measured by numbered nucleotides in reference to the UMD 3.1 genome assembly (http://bovinegenome.org/?q=node/61; accessed 15 September 2016) or the ARS-UCD 1.2 genome assembly (https://www.animalgenome.org/repository/cattle/UMC_bovine_coordinates/; accessed 19 September 2018) in parentheses

^c^ The most significant SNP in the locus associated with heifer conception rate as identified by *rs* number which is a reference number assigned to markers submitted to the National Center for Biotechnology Information SNP database (https://www.ncbi.nlm.nih.gov/projects/SNP/; accessed 9 March 2016)

^d^ Genome-wide association model

^e^ Significance (*P-*value) of the most significant SNP associated with heifer conception rate

Many of the SNPs associated with HCR1 and TBRD did not exhibit many nearby supporting SNPs or “trees” that often are expected to accompany significant SNPs defining associated loci. Traits with dominant or recessive gene effects would exhibit these characteristics where there is a lack of surrounding significant SNPs. When associated SNPs were evaluated across the three inheritance models, many of the loci were found to have higher significance in dominant or recessive gene models than the additive gene model. While this explains why some of these regions may exhibit a paucity of supporting SNPs, further investigation revealed another factor that may have also influenced this architecture.

Structural or copy number variants (CNV) including deletions, duplications, insertions, inversions, and translocations of chromosomal regions may also impact the number of supporting SNPs underlying a detected association [42]. In cattle, it has been estimated that CNVs make up approximately 2 to 7% of the genome [43]. However, the true frequency of CNVs in cattle is not well characterized and can be influenced by breeds, sample size, CNV detection algorithms and the platforms used for genotyping/sequencing [43]. To explore this possibility, associated SNPs were investigated to determine if they were located within CNVs boundaries and if the defined boundaries of the CNV could potentially explain why neighboring SNPs were not associated with HCR1 or TBRD. To do this, SNPs were searched in Ensembl (http://ensembl.org/) [44] by location to identify if any associated SNP was located within a CNV of any kind. Of the 228 HCR1 loci, 38 loci (16.6%) were present within one or more CNVs, while 70 of the 446 TBRD loci (15.7%) were within a CNV (Additional file 1: Tables S1-S2). If only loci that were most significant in the additive model were evaluated, 6 of the 24 (25%) HCR1 loci and 7 of the 71 (9.8%) TBRD loci were located within a CNV. Additionally, SNPs that are near the boundary or edge of a CNV will also have a similar effect as SNPs within a CNV. Therefore, limiting the identification of SNPs within CNVs that are associated with HCR1 and TBRD represents a conservative estimate of loci that are affected by CNVs and that lack the typical number of supporting SNPs underlying a detected association.

Because the BovineHD BeadChip was not designed to identify CNVs, it is not a good tool for evaluating the association of CNVs with traits [45]. Therefore, we did not perform a separate CNV analysis for this study. However, previous studies in cattle have indicated that CNVs have a role in fertility. For example, a study in Nordic Red dairy cattle breeds identified a 660 kb deletion on BTA12 that was associated with impaired cow fertility [46]. While another study by McDaneld et al. (2014) [47] identified a deletion of approximately 70 kb in length on BTA5 that was associated with reduced reproductive efficiency in multiple *Bos indicus* populations. The identification of many significant loci within CNVs in the current study supports the supposition that CNVs may play an important role in bovine female fertility. Additional CNV analyses across a diverse array of bovine breeds are needed to fully elucidate loci associated with HCR1 and TBRD.

Finally, a lack of supporting SNPs surrounding the lead SNPs associated with HCR1 and TBRD may be an indication that some of these associations are false positives. The loci at greatest risk for being a false positive are 9 additive loci associated with HCR1, and 18 additive loci (including 2 QTL that were shared with HCR1) associated with TBRD that were not present within a CNV, and that had not been found to be associated with fertility in a previous study. To reduce the possibility that false positive results were reported, a high significance threshold was imposed (*P* < 5 × 10^− 8^) for an association with HCR1 and TBRD. To identify if other factors may be contributing to a lack of supporting SNPs, the density and linkage disequilibrium of SNPs surrounding each locus was examined and the minor allele frequency (MAF) of the associated SNP was evaluated to determine if it was less than 0.05 (rare). After investigating the density of SNPs surrounding the 25 associated loci, there was no evidence of a lack of SNPs flanking these loci. Rare MAF (0.01 < MAF < 0.05) may also influence the possibility of a false positive. However, none of the nine loci associated with HCR1 had a MAF < 0.05 that might have enhanced the likelihood of a false positive, although two of the 18 loci associated with TBRD had MAF = 0.04 (BTA13 at 3.7 Mb and BTA17 at 45.5 Mb). Therefore, it is possible that some proportion of the 25 associated SNPs which exhibited no further marker-based support are false positives. This determination will only be possible through additional validation studies in independent populations.

Of the four most significant loci shared between HCR1 and TBRD in the additive and dominant models, three have positional candidate genes (Table 3). The three loci and their positional candidate genes are: microRNA 383 (*MIR383*) on BTA27, transient receptor potential cation channel subfamily M member 7 (*TRPM7*) on BTA10, and membrane associated ring-CH-type finger 11 (*MARCH11*) on BTA20. The major functional theme identified among the positional candidate genes was cell proliferation, which is critical during early pregnancy for follicle growth, corpus luteum formation, uterine receptivity and embryo development. Mutations affecting proliferation could inhibit pregnancy recognition and implantation resulting in reduced fertility.

*MIR383, is* a microRNA that resides within intron 1 of the large zeta sarcoglycan (*SGCZ)* gene in humans, mice, and rats [48]. MicroRNAs are small non-coding RNAs of 19–25 nucleotides that post-transcriptionally regulate gene expression through binding of 3′ untranslated regions. In mice, *MIR383* acts to inhibit the transcription factor RNA binding motif *RBMS1* that interacts with *c-Myc* to repress estrogen release [48]. *Mir383* also stimulates the expression of *CYP19A1*, which is the rate limiting enzyme for estrogen synthesis [48]. In the mouse, Sgcz and Mir383 function together to affect hormone synthesis, endometrial receptivity and embryo growth [48]. While little research exists on *MIR383* function in cattle, work in other species emphasizes that a mutation affecting *MIR383* could impair pregnancy success.

*TRPM7* is involved in multiple cellular processes and has a high affinity for calcium and magnesium [49, 50]. Calcium regulates numerous processes ranging from cell motility to cell proliferation, including the resumption of meiosis after fertilization occurs [51]. Similarly, magnesium plays a fundamental role during embryonic development in humans, as its deficiency has been linked to defects in fetal growth and development [52]. The genetic ablation of *TRPM7* results in death before day 7.5 of embryogenesis in mice, suggesting a role in pre-implantation development through dysregulation of magnesium homeostasis in oocytes and embryos [53]. Zinc is another ion that utilizes *TRPM7* channels which is also involved in fertility and embryonic development. Zinc highly regulates oocyte maturation [54] that begins with the meiotic division of growing oocytes. Oocytes arrest at prophase I until ovulation occurs at which time meiosis continues until metaphase II, where it arrests again until fertilization [55]. Once fertilized, the oocyte proceeds through meiosis and begins the mitotic division of embryogenesis [55]. Studies have suggested that intracellular deficiency of zinc provokes meiotic arrest shortly after ovulation in mammals [54, 56], which can lead to nonviable embryos and implantation failure. Therefore, mutations affecting the ability of a fertilized embryo to successfully proceed through meiosis have the potential to result in pregnancy loss.

Unlike *MIR383* and *TRPM7*, little is known about the role of *MARCH11,* especially in female fertility. *MARCH11* has been implicated in male fertility through its role of protein sorting in spermatids and exhibits enriched expression in the testis [57]. *MARCH11* is also expressed in the brain and pituitary and targets CD4 for ubiquitination. Methylation of *MARCH11* has been identified as a poor prognostic indicator for small cell lung cancer, but its role in female fertility is unknown [58].

Network analysis identified (*P* < 0.01) two IPA canonical pathways, G - protein coupled receptor signaling and cAMP - mediated signaling pathways, that were associated with the positional candidate genes for HCR1 and TBRD (Table 4). The G - protein coupled receptor signaling pathway included 14 positional candidate genes and of these, 10 were shared with the cAMP - mediated signaling pathway. There was a total of 12 positional candidate genes present in the cAMP - mediated signaling pathway.

**Table 4:** Ingenuity Pathway Analysis canonical pathways associated with heifer conception rate (B-H *P* < 0.01)

Canonical Pathways^a^	B-H *P*-value^b^	Positional Candidates Genes
cAMP-Mediated Signaling	0.0041	*ADCY5, AKAP9, CAMK2D, CHRM3, CNGA3, ENPP6, GRM8, PDE11A, PDE4D, PDE5A, RGS10, RGS7*
G-Protein Coupled Receptor Signaling	0.0041	*ADCY5, CAMK2D, CHRM3, ENPP6, GNAQ, GRM8, IKBKE, PDE11A, PDE4D, PDE5A, PIK3CB, PIK3R1, RGS10, RGS7*

^a^ Canonical pathways identified in the Ingenuity Pathway Analysis with positional candidate genes associated with heifer conception rate at first service and/or the number of times bred to become pregnant

^b^ Benjamini-Hochberg multiple testing correction significance value for the Ingenuity Pathway Analysis

Although no upstream regulators were identified that were significant, 197 master regulators were identified (*P* < 0.01) (Additional file 1: Table S3). The master regulator that was most significant (*P* = 3 × 10^− 4^) was E2F transcription factor 3 (*E2F3*), which is down regulated during the window of implantation in human stromal cells of the uterus [59]. This transcription factor plays a role in cell proliferation, cell cycle progression, apoptosis and the transition of the cell from the G1 and S phase [60, 61]. It also has been associated with intrauterine growth retardation and fetal growth restriction through its target gene *miR-141* [62]. The E2F transcription factor indirectly regulates 87 positional candidate genes associated with HCR1 and TBRD including the shared positional candidates *MIR383* and *SGCZ* (Additional file 1: Table S3). There were 67 significant (*P* < 0.01) master regulators for positional candidate gene *MIR383* and 66 for *SGCZ* but no master regulators were significant for *MARCH11* or *TRPM7.* Most of the master regulators for *MIR383* and *SGCZ* were shared as only seven master regulators were unique to *MIR383* and six master regulators were unique to *SGCZ* (Additional file 1: Table S4).

After comparing the loci identified in the current study with the 22 previous GWAA, 46 genomic regions were validated (Table 5). These loci were identified both within the Holstein breed [19, 33, 35, 37–39] and among other breeds such as crossbreed Angus [34], Brahman [40], Brangus [40], Jersey [19] and a tropical composite breed [40]. Identifying genomic regions in independent populations and across breeds suggests that these regions are near the causal mutation and have a common function related to fertility in cattle. Ultimately, the identification of causal mutations that are associated with fertility will allow the dairy and beef industries to more efficiently use genomic selection to make genetic improvement without the need to continually reassess the validity of markers that are only in LD with the causal mutations and to better understand the reproductive process.

**Table 5:** Validated loci associated with cattle fertility

BTA^a^	Region (Mb)^b^	Previous Studies^c^	Previous Phenotype(s)^d^	Previous Breeds^e^	Current Study Phenotype(s)^f^
1	61–62	1 [37]	AISC	Holstein^N^	HCR1 & TBRD
1	68–69	1 [37]	AISH	Holstein^N^	HCR1 & TBRD
1	147–148	1 [37]	AISC	Holstein^N^	HCR1 & TBRD
2	53–54	1 [37]	AISC	Holstein^N^	HCR1 & TBRD
2	123–124	1 [37]	AISH	Holstein^N^	HCR1 & TBRD
2	136–137	1 [33]	CI	Holstein^It^	HCR1 & TBRD
3	90–91	1 [37]	AISC	Holstein^N^	HCR1 & TBRD
4	29–30	1 [37]	AISC	Holstein^N^	TBRD
4	37–38	1 [40]	Reproduction	Brangus^U^, Brahman^A^, Tropical Composite^A^	HCR1 & TBRD
5	64–65	1 [40]	Reproduction	Brangus^U^, Brahman^A^, Tropical Composite^A^	TBRD
5	88–89	1 [36]	ICF	Holstein^Ca^	HCR1 & TBRD
5	107–108	2 [19, 37]	CI [19]; IFLC & ICF [37]	Holstein^A,I,N^, Jersey^A^	HCR1 & TBRD
5	112–113	1 [37]	AISC	Holstein^N^	TBRD
5	116–117	1 [33, 37]	NRRC [33]; AISC [37]	Holstein^It,N^	HCR1
5	118–119	1 [37]	AISC	Holstein^N^	TBRD
6	23–24	1 [38]	DO & SC	Holstein^U^	HCR1 & TBRD
6	92–93	2 [19, 37]	CI [19]; FTI & ICF [37]	Holstein^A,I,N^, Jersey^A^	HCR1 & TBRD
7	43–44	1 [37]	AISC	Holstein^N^	TBRD
8	20–21	1 [37]	AISC, AISH	Holstein^N^	HCR1 & TBRD
8	91–92	1 [33]	CI	Holstein^It^	TBRD
11	8–9	1 [37]	AISC	Holstein^N^	TBRD
13	45–46	1 [37]	AISC	Holstein^N^	TBRD
14	1–2	3 [19, 37]	CI [19]; AISC, FTI, IFLC, & NRRC [37]; HCR & CCR	Holstein^A,I,N,U^, Jersey^A^	TBRD
14	82–83	2 [33, 37]	AISH [37]; NRRC [33]	Holstein^It,N^	HCR1
16	21–22	1 [34]	P28	Angus crossbreed^U^	HCR1 & TBRD
16	36–37	1 [40]	Reproduction	Brangus^U^, Brahman^A^, Tropical Composite^A^	HCR1 & TBRD
17	3–4	2 [19, 37]	CI [19]; AISH & IFLC [37]	Holstein^A,I,N^, Jersey^A^	HCR1 & TBRD
17	14–15	1 [37]	AISC	Holstein^N^	TBRD
17	72–73	2 [19, 37]	CI [19]; NRRC [37]	Holstein^A,I,N^, Jersey^A^	HCR1 & TBRD
18	61–62	2 [19]	CI [19]; DPR	Holstein^A,I,N^, Jersey^A^	HCR1 & TBRD
18	62–63	3 [19, 35, 37]	CI [19]; FTI & IFLC [37]; P42 [35]	Holstein^A,I,N^, Jersey^A^	HCR1 & TBRD
18	62–63	3 [19, 35, 37]	CI [19]; FTI & IFLC [37]; P42 [35]	Holstein^A,I,N^, Jersey^A^	HCR1 & TBRD
18	47–48	1 [33]	DFS	Holstein^It^	TBRD
20	27–28	1 [37]	AISC	Holstein^N^	TBRD
21	54–55	2 [19, 37]	CI [19]; NRRC [37]	Holstein^A,I,N^, Jersey^A^	HCR1
22	49–50	2 [19, 37]	CI [19]; ICF [37]	Holstein^A,I,N^, Jersey^A^	TBRD
22	49–50	2 [19, 37]	CI [19]; ICF [37]	Holstein^A,I,N^, Jersey^A^	TBRD
23	50–51	1 [38]	CR1	Holstein^Ca,N^	TBRD
24	60–61	1 [37]	AISC	Holstein^N^	TBRD
26	27–28	1 [37]	AISC	Holstein^N^	HCR1 & TBRD
26	34–35	2 [19, 37]	CI [19]^;^ AISC, NRRC, & NRRH [37]	Holstein^A,I,N^, Jersey^A^	HCR1
26	35–36	1 [37]	AISC	Holstein^N^	HCR1
28	8–9	1 [37]	AISC	Holstein^N^	HCR1 & TBRD
29	14–15	1 [37]	AISH	Holstein^N^	TBRD
29	16–17	1 [37]	AISH	Holstein^N^	TBRD
29	50–51	1 [37]	AISC	Holstein^N^	TBRD

^a^ *Bos taurus* chromosome (BTA) location of the locus

^b^ Region associated locus is located in (in Mb) as measured by numbered nucleotides in reference to the UMD 3.1 genome assembly (http://bovinegenome.org/?q=node/61; accessed 15 September 2016)

^c^ Number of previous studies locus previous associated in is listed. The citation number for each study is listed in superscript brackets

^d^ Traits previously associated with loci abbreviated as follows: *AISC* number of inseminations to conception in cows, *AISC* number of inseminations to conception in heifers, *CI* calving interval, *CCR* cow conception rate, *CR1* conception rate to first insemination in cows, *DFS* days to first service, *DO* days open, *DPR* daughter pregnancy rate, *FTI* fertility index, *HCR* heifer conception rate, *ICF* interval (in days) from calving to first insemination, *IFLC* days from first to last insemination in cows, *NRRC* 56 day non return rate in cows, *NRRH* 56 day non return rate in heifers, *P28* pregnancy success at day 28 post embryo transfer, *P42* pregnancy success within first 42 days of mating, *SC* services per conception. If multiple traits are listed from different studies the citation number for study is listed in superscript brackets

^e^ Cattle breeds loci were previously identified are listed with the country or region the population was from indicated in superscript as follows: Australian - A; Canadian - Ca; Chinese - C, Irish - I; Italian - It; Nordic - N; United States - U

^f^ Phenotype of the current study the loci was associated with: conception rate at first AI service - HCR1; number of breedings to conception - TBRD

Using marker-based relationship matrices (EMMAX), the pseudo-heritability was estimated to be 0.64 for HCR1 and 0.76 for TBRD, whereas heritability estimates were somewhat lower using AI-REML (i.e. 0.56 for HCR1 and 0.74 for TBRD). Moreover, both methods produced heritability estimates that were much higher than those previously reported for fertility traits [5, 6, 10, 63–67]. However, it should be noted that the study design utilized here, which included the analysis of genotypes for single-population heifers selected from the tails of the observed phenotypic distributions, results in a sampling bias. This sampling bias can lead to inflated heritability estimates that would not be seen in a large randomly selected population. However, heifer enrollment from the tails of the phenotypic distribution theoretically facilitates the detection of moderate and large-effect loci with fewer samples, thus making it an attractive approach to GWAA.

Relative to the heritability estimates produced herein, these estimates may also increase when environmental effects are minimized, and the accuracy of phenotypic measurement is maximized. In this study the phenotypes were precisely measured and sampled from a single population and location where environmental effects were minimized. Larger studies where heritability estimates were produced by sampling many populations and locations would be expected to have greater environmental effects, greater variations in the measurement of the phenotypes, and much smaller heritability estimates (Table 6) [5, 6, 10, 63–67]. For example, a previous study utilizing a threshold model estimated heritability for HCR at 0.01, based on reproductive data from 362,512 Holstein heifers ranging in age from 11 to 27 months, which were bred by AI between March 2003 and August 2005 [64]. It is difficult to directly compare or reconcile this estimate with the results of the current study (i.e., 3300 heifers that ranged in age from 12 to 14 months and were bred at the same facility over a period of only a few months). In addition, caution must be used when comparing heritability estimates for heifer or cow fertility traits when the phenotypes, management strategies, fertility status of the sires used for breeding, and structure of the populations under investigation are quite heterogeneous (Table 6).

**Table 6:** Heritability estimates for fertility traits in dairy cattle worldwide^1^

Country(s)	Citation^2^	Trait^3^	Breed(s)	*h* ^*2*^
Austria, Germany, and Luxembourg	6	NRH	Holstein, Jersey, Red Dairy Cattle	0.012
Austria, Germany, and Luxembourg	52	FSh	Holstein, Jersey, Red Dairy Cattle	0.014
Canada	6	NRH	Brown Swiss, Guernsey, Holstein	0.015
Czech Republic	6	CR^a^	Holstein	0.039
Denmark, Finland, and Sweden	6	CR^b^	Holstein	0.01
Flanders and Netherlands	6	NRH	All breeds	0.018
France	6	HCO	Brown Swiss, Holstein	0.02
Iran	54	INS^a^	Holstein	0.046
Israel	55	HCR^a^	Holstein	0.0207
Italy	56	INS^b^	Brown Swiss	0.026
Norway	5	NRH	Norwegian Red Cattle	0.01
Poland	6	NRH	Holstein	0.019
Switzerland	6	NRH	Holstein	0.013
United States	6	HCR^b^	Aryshire, Brown Swiss, Guernsey, Holstein, Jersey, Milking Shorthorn	0.01
United States	57	HCR^b^	Holstein	0.04
United States	10	HCR^c^	Brown Swiss, Holstein, Jersey	0.01
United States	Current Study	HCR^a^	Holstein	0.61

^1^Represents a sampling of studies that report heritability estimates for fertility traits

^2^Citations are numbered as they appear in the literature cited

^3^Abbreviations for traits tested: CR^a^ and HCO is conception rate for up to 3 services for maiden heifers, CR^b^ is conception rate for maiden heifers for an undefined number of services, FSh is the interval from first to successful insemination which ranged from 0 to 600 days, INS^a^ is the number of inseminations to conception for up to 9 services in maiden heifers, INS^b^ is the number of inseminations to conception with number of services unreported, HCR^a^ is the number of services to achieve conception for up to 5 services for maiden heifers, HCR^b^ is the number of services to achieve conception for up to 7 services for maiden heifers, HCR^c^ is the number of services to achieve conception and is undefined, and NRH is the nonreturn rate of 56 days in maiden heifers

The fertility phenotypes used for genomic selection are diverse, and a more standardized phenotype for heifers would facilitate comparisons of heritability estimates and loci associated with fertility [54]. As shown in Table 4, many different definitions of fertility traits are currently being used in the dairy industry, however, few include the number of services required to achieve a pregnancy. Non-return rate to 56 days and the interval of time from the first insemination to successful insemination are two examples of heifer fertility measures where the number of services are not considered. Differences in heritability estimates reported in this study and those conducted previously may also partly be due to the differences in the phenotypes measured [63–67].

## Conclusion

Additive and non-additive loci identified in this study have functional relevance in HCR1 and TBRD and make excellent positional as well as functional candidate genes for future investigations. The detected and validated loci provide a mechanism for further evaluation via incorporation into genomic selection programs, and to potentially assist in choosing replacement animals based on conception rate in heifers. These loci also provide a foundation for future studies into the mechanisms that affect pregnancy establishment and maintenance in heifers. Reducing the considerable economic losses associated with poor reproductive performance in dairy cattle requires the simultaneous development of genomic tools to enable the selection of heifers with superior fertility and selection of bulls whose daughters will be more fertile, enabling a longer reproductive and productive life.

## Additional file

Additional file 1: **Table S1.** Loci associated with heifer conception rate at the first AI service (HCR1). **Table S2.** Loci associated with heifer conception rate to repeated AI services (TBRD). **Table S3.** Master regulators associated with heifer conception rate positional candidate genes. **Table S4.** Master regulators associated with top positional candidate genes. (PDF 1184 kb)

## Data Availability

The data used and analyzed in the current study are available for non-commercial use via MTA from the corresponding author.

## Abbreviations

AI: Artificial insemination
AI-REML: Average information restricted maximum likelihood
BTA: *Bos taurus* chromosome
CNV: Copy number variant
EMMAX: Efficient mixed model association eXpedited using a relationship matrix
GBLUP: Genomic best linear unbiased prediction
GWAA: Genome-wide association analysis
HCR: Heifer conception rate
HCR1: Heifer conception rate at first service
QTL: Quantitative trait loci
SNP: Single nucleotide polymorphism
SVS: SNP and variation suite software
TBRD: Number of times bred required for pregnancy
