# Supplementary material for: Validation of 46 loci associated with female fertility traits in cattle
Source: BMC Genomics. 2019 Jul 12;20:576. doi: 10.1186/s12864-019-5935-3 (PMC6624949; doi:10.1186/s12864-019-5935-3)
Supplement: Supplementary file 1 — Table S1. Loci associated with heifer conception rate at the first AI service (HCR1). Table S2. Loci associated with heifer conception rate to repeated AI services (TBRD). Table S3. Master regulators associated with heifer conception rate positional candidate genes. Table S4. Master regulators associated with top positional candidate genes. (PDF 1184 kb) [file 12864_2019_5935_MOESM1_ESM.pdf]

Table S1. Loci associated with heifer conception rate at the first AI service (HCR1).

BTA[1]	ARS 1.2<br>BP Position[3]	UMD 3.1<br>BP Position[2]	SNP ID[4,5]	Model[6]	P-Value[7]
UNK	UNK	BTA 6<br>100119868	*rs41594506*	Additive<br>Dominant	1.68×10^{-10}<br>1.51×10^{-10}
1	4253950	3546098	*rs136767715*	Additive<br>Dominant	8.21×10^{-10}<br>3.14×10^{-19}
1	13869391	13296572	*rs132784449**	Additive<br>Dominant	8.87×10^{-11}<br>2.06×10^{-13}
1	15273288	14740447	*rs109845303*	Recessive	3.31×10^{-11}
1	22024056	21525169	*rs42273186*	Dominant	4.01×10^{-08}
1	24140382	23652573	*rs136178026*	Recessive	3.01×10^{-09}
1	32344466	31910495	*rs42869460**	Additive<br>Dominant	7.47×10^{-09}<br>6.79×10^{-10}
1	32895270	32462420	*rs43222212**	Additive<br>Dominant	3.09×10^{-09}<br>5.34×10^{-09}
1	36708125	36308223	*rs132824212*	Dominant	6.96×10^{-12}
1	60732928	61283063	*rs133972911*	Additive<br>Dominant	2.07×10^{-09}<br>2.94×10^{-10}
1	67838260	68436471	*rs110693447**	Dominant	1.52×10^{-09}
1	69188246	69793194	*rs137088827*	Recessive	7.49×10^{-09}
1	126636424	127635326	*rs134321837*	Additive<br>Dominant	6.80×10^{-09}<br>2.00×10^{-09}
1	146156382	147891529	*rs134566710*	Dominant	3.33×10^{-08}
2	5758799	5691695	*rs110306500*	Additive<br>Dominant	1.48×10^{-11}<br>1.73×10^{-12}
2	19157717	19155572	*rs42267687*	Dominant	6.32×10^{-09}
2	32519977	32622594	*rs133040970*	Dominant	2.93×10^{-08}
2	43012494	43122845	*rs43295963*	Additive<br>Dominant	1.09×10^{-10}<br>8.24×10^{-13}
2	53681112	53783708	*rs135006704*	Additive<br>Dominant	6.48×10^{-09}<br>1.20×10^{-10}
2	56408154	56629380	*rs132792158**	Additive	1.24×10^{-08}
2	70452684	70815583	*rs110836558*	Dominant	2.68×10^{-09}
2	71052083	71417750	*rs134623977*	Additive<br>Dominant	3.43×10^{-10}<br>6.13×10^{-12}
2	71825380	72241749	*rs110643377*	Recessive	4.08×10^{-08}
2	85066770	85462609	*rs109342415*	Additive<br>Dominant	1.07×10^{-08}<br>5.22×10^{-12}
2	97738219	98167527	*rs41641857*	Additive<br>Dominant	6.89×10^{-11}<br>1.28×10^{-13}
2	102709849	103226604	*rs137445560*	Dominant	3.91×10^{-08}
2	110256790	111014944	*rs137494641*	Dominant	3.77×10^{-09}
2	123403424	123947527	*rs110886070**	Dominant	3.35×10^{-08}
2	135499782	136075993	*rs136810779*	Additive<br>Dominant	3.56×10^{-08}<br>1.69×10^{-08}

3	18159642	18198456	*rs134651745*	Dominant	9.22×10^{-09}
3	35668422	35785053	*rs29024428*	Dominant	1.09×10^{-10}
3	37660099	37781561	*rs137663076*	Recessive	1.10×10^{-10}
3	38433068	38557710	*rs109265034*	Additive<br>Dominant	3.97×10^{-10}<br>4.74×10^{-18}
3	45740900	45896368	*rs135441304*	Dominant	1.64×10^{-08}
3	76550807	76976989	*rs42920814**	Additive<br>Dominant	1.03×10^{-09}<br>2.51×10^{-12}
3	80627475	80958341	*rs43353709**	Additive<br>Dominant	3.70×10^{-08}<br>8.97×10^{-09}
3	84895863	85442743	*rs43272773*	Recessive	7.14×10^{-09}
3	90106058	90685311	*rs109308461*	Additive<br>Dominant	2.03×10^{-08}<br>5.50×10^{-09}
3	97504864	98096750	*rs29024665*	Dominant	1.43×10^{-10}
3	114805862	115411407	*rs110343443**	Dominant	2.03×10^{-10}
3	115082809	115692498	*rs109046293**	Dominant	1.31×10^{-08}
3	115629791	116253052	*rs133851967**	Additive<br>Dominant	1.04×10^{-09}<br>1.45×10^{-09}
4	8676114	8500879	*rs136443753*	Dominant	1.44×10^{-08}
4	9354119	9200721	*rs110530879**	Additive<br>Dominant	1.26×10^{-09}<br>1.72×10^{-14}
4	22888285	22831648	*rs109094478**	Dominant	2.18×10^{-09}
4	37056340<br><br>37439721	37235676<br><br>37618902	*rs133417267*<br><br>*rs43388620*	Additive<br>Dominant<br>Additive<br>Recessive	6.93×10^{-21}<br>6.93×10^{-21}<br>2.95×10^{-09}<br>5.18×10^{-10}
4	53811395	54127019	*rs43394990*	Additive<br>Dominant	1.19×10^{-09}<br>3.35×10^{-10}
4	70990701	71442761	*rs110114859*	Dominant	1.29×10^{-10}
4	119088045	119886948	*rs137275654*	Dominant	1.11×10^{-10}
5	652247	645386	*rs133539307*	Dominant	1.47×10^{-10}
5	12363691	12435196	*rs134137475*	Dominant	1.11×10^{-08}
5	27683283	27837406	*rs134147281*	Recessive	8.18×10^{-10}
5	35745274	35925646	*rs135774967*	Additive<br>Recessive	5.93×10^{-12}<br>1.39×10^{-10}
5	36663436	36860611	*rs136252597*	Additive<br>Dominant	1.43×10^{-08}<br>1.43×10^{-08}
5	52254169	52496943	*rs41657468*	Additive<br>Dominant	2.47×10^{-10}<br>3.88×10^{-12}
5	62021071	62362183	*rs109867733*	Additive	3.43×10^{-08}
5	69286140	69623431	*rs108967945*	Additive<br>Dominant	4.74×10^{-10}<br>2.52×10^{-10}
5	87901217	88311948	*rs43439308*	Dominant	1.60×10^{-08}
5	88432158	88850154	*rs109772503*	Dominant	7.64×10^{-10}
5	89543284	89964756	*rs136764099*	Additive<br>Dominant	4.15×10^{-10}<br>4.15×10^{-10}

5	92751411	93180224	*rs134023889*	Additive<br>Recessive	4.17×10^{-08}<br>3.54×10^{-08}
5	93632027	94060598	*rs109606410*	Additive<br>Dominant	4.91×10^{-08}<br>1.08×10^{-08}
5	97645945	98117091	*rs41592957*	Additive<br>Dominant	6.22×10^{-09}<br>5.92×10^{-11}
5	106786883	107278032	*rs109634884*	Additive<br>Dominant	1.05×10^{-09}<br>9.70×10^{-10}
5	106910293	107407965	*rs133201447*	Additive<br>Dominant	2.07×10^{-10}<br>2.38×10^{-13}
5	115195553	115913514	*rs136393747*	Additive<br>Dominant	6.03×10^{-12}<br>2.12×10^{-13}
5	115751204	116478955	*rs110477071*	Additive	2.32×10^{-08}
6	11990040	13154909	*rs43450481*	Dominant	7.83×10^{-12}
6	20288492	21570560	*rs137779599*	Additive<br>Dominant	4.04×10^{-09}<br>7.27×10^{-09}
6	21839136	23165378	*rs43097424*	Recessive	5.89×10^{-10}
6	24944761	26314684	*rs135805690*	Additive<br>Dominant	4.12×10^{-08}<br>1.24×10^{-09}
6	28927865	30352852	*rs108947176*	Additive<br>Dominant	2.00×10^{-09}<br>1.09×10^{-13}
6	29353493	30779206	*rs109717767*	Dominant	3.58×10^{-08}
6	36107083	37533544	*rs110377022*	Dominant	8.04×10^{-10}
6	61819817	63475480	*rs109624509*	Additive	1.98×10^{-09}
6	64697753	66339518	*rs137591215*	Recessive	3.23×10^{-08}
6	79765417	81489249	*rs135378647**	Dominant	3.62×10^{-08}
6	88327574	90075383	*rs41870471*	Dominant	1.15×10^{-08}
6	91512574	93263835	*rs43476570*	Additive<br>Dominant	1.49×10^{-10}<br>2.73×10^{-15}
6	96325620	98099748	*rs110349384*	Additive<br>Dominant	2.43×10^{-08}<br>1.41×10^{-13}
7	10109598	10099512	*rs134195407**	Dominant	2.12×10^{-11}
7	13875817	14998140	*rs133202371*	Additive	7.40×10^{-10}
7	56111544	58055640	*rs133170783*	Additive<br>Dominant	6.56×10^{-10}<br>1.27×10^{-14}
7	83885916	86198271	*rs135291987*	Additive	2.83×10^{-08}
7	92133550	94537487	*rs135142549*	Additive	1.02×10^{-08}
7	109134936	111730542	*rs109635492**	Recessive	2.37×10^{-09}
8	3030778	2898483	*rs134614161*	Additive<br>Dominant	2.50×10^{-08}<br>2.40×10^{-11}
8	13184292	13104883	*rs133345372*	Additive<br>Dominant	4.12×10^{-09}<br>6.99×10^{-12}
8	20500129	20510016	*rs134858319*	Additive<br>Dominant	1.24×10^{-09}<br>2.92×10^{-12}
8	25111465	25123206	*rs110623317*	Dominant	4.65×10^{-14}
8	25649947	25665062	*rs42207644*	Dominant	8.45×10^{-09}

8	45340654	45624366	rs109293388	Dominant	2.45×10^{-08}
8	45456855	45740396	rs110505607	Additive<br>Dominant	1.32×10^{-08}<br>8.57×10^{-09}
8	53769149	54054285	rs135532670	Additive<br>Dominant	2.85×10^{-08}<br>2.22×10^{-11}
8	57193589	57592438	rs109714363	Additive	2.89×10^{-08}
8	82147864	83569006	rs109389362	Dominant	6.47×10^{-09}
8	82885198	84307178	rs43137599	Additive<br>Dominant	5.23×10^{-09}<br>1.56×10^{-11}
8	98652238	100328375	rs134841549	Additive	3.83×10^{-09}
8	106835109	108601170	rs43580717	Additive<br>Dominant	4.26×10^{-11}<br>3.03×10^{-16}
9	5812857	5919500	rs133149639*	Additive<br>Dominant	1.28×10^{-11}<br>5.63×10^{-14}
9	17803245	18022184	rs135175889	Additive<br>Dominant	7.97×10^{-13}<br>1.92×10^{-14}
9	17804516	18023455	rs136675875	Recessive	5.45×10^{-09}
9	37734137	38199983	rs134755337	Recessive	1.01×10^{-09}
9	51946932	52678477	rs42330916	Dominant	9.43×10^{-09}
9	64389223	65306072	rs43602336	Additive<br>Dominant	2.34×10^{-11}<br>5.04×10^{-12}
10	18579855	18557936	rs133730050	Additive<br>Dominant	1.49×10^{-08}<br>1.15×10^{-11}
10	20950434	20784448	rs797745899	Additive<br>Dominant	6.48×10^{-11}<br>3.47×10^{-15}
10	35290328	35397418	rs134282076	Additive<br>Dominant	1.22×10^{-08}<br>1.38×10^{-09}
10	37088125	37205266	rs135421839	Additive<br>Dominant	3.15×10^{-13}<br>6.89×10^{-15}
10	39805634	39894434	rs43623068*	Additive	2.71×10^{-08}
10	40517970	40596210	rs43619838	Additive<br>Dominant	4.69×10^{-08}<br>2.03×10^{-14}
10	55502976	55575425	rs135035943	Dominant	9.46×10^{-10}
10	59695958	59882200	rs110617366*	Additive<br>Dominant	7.12×10^{-18}<br>1.34×10^{-23}
10	65704210	65934611	rs137047345	Dominant	2.50×10^{-08}
10	72003636	72249788	rs133399594	Dominant	2.73×10^{-08}
10	84004823	84382827	rs135037566	Dominant	7.24×10^{-09}
10	96305589	97382623	rs110432233	Dominant	1.30×10^{-09}
10	98022135	99101604	rs109836144	Additive<br>Dominant	4.70×10^{-08}<br>3.82×10^{-11}
11	2159226	2125437	rs133812771	Additive<br>Dominant	1.99×10^{-12}<br>1.34×10^{-20}
11	16856558	16878438	rs109265815	Dominant	2.32×10^{-10}
11	18217194	18238034	rs42859776	Recessive	1.08×10^{-08}
11	23805191	23867860	rs42494516*	Additive<br>Dominant	4.76×10^{-08}<br>4.30×10^{-11}

11	66038610	66015350	*rs136757739*	Dominant	8.38×10^{-09}
11	66697709	66672407	*rs109684663*	Additive<br>Dominant	3.48×10^{-09}<br>1.13×10^{-09}
11	75173290	75222305	*rs109611855**	Additive<br>Dominant	4.13×10^{-10}<br>8.76×10^{-13}
11	86434490	86400864	*rs110541854*	Additive<br>Dominant	4.51×10^{-10}<br>1.48×10^{-15}
11	106385151	105731694	*rs134749626*	Additive<br>Dominant	1.08×10^{-08}<br>4.37×10^{-09}
12	1482941	1421632	*rs110192562*	Dominant	1.71×10^{-08}
12	19794783	19889431	*rs133053197*	Dominant	4.18×10^{-11}
12	22522443	22541000	*rs137091747*	Additive<br>Dominant	1.27×10^{-10}<br>2.81×10^{-12}
12	26581859	26607578	*rs41623530*	Additive	2.64×10^{-08}
12	45305543	45608128	*rs134957168**	Additive<br>Dominant	1.61×10^{-09}<br>1.01×10^{-10}
12	50157779	50503658	*rs134603164*	Dominant	9.81×10^{-11}
12	63170379	62737864	*rs42629844*	Additive<br>Dominant	1.75×10^{-08}<br>2.60×10^{-09}
12	74729747	78603323	*rs109163624**	Dominant	6.32×10^{-09}
12	74894376	78821023	*rs108947709**	Dominant	4.54×10^{-09}
13	20548292	20828712	*rs109545965**	Additive<br>Dominant	2.17×10^{-09}<br>2.56×10^{-13}
13	21464768	21747463	*rs135877047*	Dominant	3.52×10^{-11}
13	25085213	25356593	*rs134588643*	Dominant	2.07×10^{-10}
13	31629599	31920795	*rs109574513*	Additive<br>Dominant	2.47×10^{-09}<br>3.43×10^{-15}
13	76130450	76853627	*rs109101250*	Dominant	3.11×10^{-09}
13	76699693	77423875	*rs137366058*	Dominant	1.79×10^{-08}
14	47975068	50136890	*rs110882087*	Additive<br>Dominant	5.32×10^{-10}<br>7.22×10^{-11}
14	48773447	50934575	*rs132818847**	Dominant	4.97×10^{-09}
14	76531942	78909801	*rs134190366*	Dominant	1.42×10^{-11}
14	80383054	82757796	*rs134134197*	Dominant	4.04×10^{-08}
15	13118554	13386488	*rs136681359*	Recessive	1.74×10^{-08}
15	26169835	26550309	*rs135503279*	Dominant	8.08×10^{-10}
15	46515608	47135215	*rs109396578*	Additive<br>Dominant	3.13×10^{-08}<br>1.19×10^{-08}
15	58102169	58881836	*rs41778290*	Recessive	1.45×10^{-08}
15	72865236	73737971	*rs132761388*	Dominant	4.78×10^{-11}
16	5730798	5581530	*rs43170110**	Recessive	2.32×10^{-08}
16	13023901	13642631	*rs110520766*	Dominant	1.18×10^{-09}
16	15837070	16473798	*rs133881641*	Dominant	2.64×10^{-11}
16	16552852	17192121	*rs136621576*	Additive<br>Dominant	1.01×10^{-09}<br>1.23×10^{-09}

16	21156409	21841998	*rs110460121*	Additive<br>Dominant	1.47×10^{-09}<br>2.78×10^{-11}
16	35699058	36501036	*rs135448881*	Dominant	1.07×10^{-08}
16	51275917	52474552	*rs109265376*	Additive	3.99×10^{-08}
16	56379164	57841028	*rs136790754*	Dominant	3.74×10^{-08}
16	76545440	78465065	*rs42426546*	Recessive	5.30×10^{-09}
16	78779992	80702862	*rs41825612*	Dominant	6.67×10^{-11}
17	3604675	3595026	*rs109750106*	Dominant	2.83×10^{-09}
17	5614491	5596017	*rs132777965*	Additive<br>Dominant	5.74×10^{-11}<br>5.05×10^{-11}
17	12688336	12853755	*rs41835005*	Dominant	1.98×10^{-09}
17	24539182	24864639	*rs110174173*	Recessive	1.03×10^{-09}
17	27878822	28295990	*rs109063837*	Additive<br>Dominant	1.67×10^{-09}<br>1.86×10^{-13}
17	52074637	54321728	*rs136973422*	Dominant	1.99×10^{-11}
17	58628136	60885707	*rs135699541*	Additive<br>Dominant	2.22×10^{-09}<br>2.14×10^{-14}
17	63557403	65811553	*rs41854091*	Dominant	6.67×10^{-13}
17	66491886	68751224	*rs42277546*	Additive<br>Recessive	8.41×10^{-10}<br>4.04×10^{-08}
17	71000696	73020606	*rs109297860**	Additive	1.64×10^{-08}
17	72726732	74741425	*rs135830763**	Dominant	8.83×10^{-09}
17	73036168	75073522	*rs134240868*	Dominant	4.83×10^{-09}
18	21851964	21929721	*rs41872094*	Additive<br>Dominant	8.88×10^{-13}<br>1.02×10^{-18}
18	41653136	41795500	*rs41878442*	Dominant	2.14×10^{-08}
18	62525015	62851490	*rs133474771**	Additive<br>Dominant	7.38×10^{-09}<br>1.11×10^{-09}
19	3088418	3140555	*rs111018952**	Dominant	3.92×10^{-09}
19	7351312	7561956	*rs109543948*	Dominant	9.04×10^{-10}
19	21820203	22388808	*rs109724289*	Dominant	1.94×10^{-09}
19	37394376	38028013	*rs135474456*	Additive<br>Dominant	7.58×10^{-09}<br>5.43×10^{-11}
19	49076932	49695304	*rs133813917*	Dominant	2.34×10^{-10}
19	52826593	53443757	*rs110291598*	Dominant	2.19×10^{-08}
20	7233587	7148044	*rs133098433*	Additive<br>Dominant	1.61×10^{-08}<br>1.44×10^{-12}
20	28002945	28016050	*rs137681615*	Additive<br>Dominant	3.80×10^{-10}<br>1.48×10^{-13}
20	35856280	35874119	*rs109846657*	Additive<br>Dominant	4.98×10^{-11}<br>1.51×10^{-12}
20	50708550	50799104	*rs110257354*	Additive<br>Dominant	2.35×10^{-08}<br>1.06×10^{-10}
20	51754819	51811579	*rs135702037**	Additive<br>Dominant	7.70×10^{-10}<br>8.64×10^{-15}
20	57038642	57113135	*rs41956232*	Additive	1.15×10^{-13}

				Dominant	3.03×10^{-18}
21	49135362	49587929	*rs133394428*	Additive<br>Dominant	4.69×10^{-08}<br>1.00×10^{-11}
21	53895321	54385593	*rs132953389**	Additive<br>Recessive	2.59×10^{-08}<br>5.61×10^{-10}
21	54515752	54994332	*rs136941405*	Additive<br>Dominant	3.14×10^{-11}<br>2.52×10^{-16}
21	68574792	70226760	*rs134541128**	Additive<br>Dominant	7.45×10^{-09}<br>6.41×10^{-14}
22	25751908	25864314	*rs136479407*	Dominant	4.49×10^{-08}
22	34038154	34155564	*rs136054280*	Recessive	2.77×10^{-09}
23	8682465	8627994	*rs43706099**	Additive<br>Dominant	8.24×10^{-09}<br>1.08×10^{-13}
23	17235589	17209231	*rs133988650*	Additive<br>Dominant	2.41×10^{-08}<br>8.49×10^{-13}
23	28191377	27994640	*rs109111192**	Dominant	1.07×10^{-08}
24	9496604	9791008	*rs133693422*	Dominant	1.11×10^{-09}
24	16026309	16334958	*rs135817307*	Additive<br>Dominant	3.38×10^{-09}<br>1.27×10^{-08}
24	50038623	50503943	*rs110770205*	Additive<br>Recessive	1.96×10^{-08}<br>1.74×10^{-13}
25	4151257	4169181	*rs110089590**	Additive<br>Recessive	6.48×10^{-11}<br>6.68×10^{-10}
25	10520959	10582476	*rs135367380*	Dominant	2.82×10^{-08}
25	11861787	11932191	*rs109087355*	Dominant	1.99×10^{-08}
25	15996288	16101786	*rs133945325*	Dominant	2.64×10^{-08}
25	27712255	27970117	*rs110492017*	Dominant	2.05×10^{-08}
26	11615909	11649512	*rs137571018*	Additive<br>Dominant	1.03×10^{-08}<br>3.17×10^{-09}
26	26715627	26951677	*rs132780515*	Additive<br>Dominant	4.98×10^{-08}<br>2.21×10^{-08}
26	28538139	28800534	*rs42434955*	Dominant	2.46×10^{-11}
26	35017936	35310973	*rs136614335*	Dominant	2.54×10^{-08}
26	37715873	38049450	*rs42099948*	Dominant	1.70×10^{-08}
26	38623922	38963804	*rs109628657*	Dominant	5.24×10^{-11}
26	39626818	39969867	*rs109711181*	Dominant	7.44×10^{-09}
26	40043343	40388206	*rs110748061*	Dominant	3.24×10^{-08}
26	40044518	40389381	*rs134644578*	Additive<br>Dominant	4.44×10^{-08}<br>2.26×10^{-10}
27	22307598	21375791	*rs132728892*	Additive<br>Dominant	8.35×10^{-52}<br>8.35×10^{-52}
27	23516317	22594468	*rs43331413*	Additive<br>Dominant	7.15×10^{-12}<br>9.16×10^{-13}
27	41475841	41350583	*rs135995504*	Dominant	2.00×10^{-08}
28	12450076	12543659	*rs109485541*	Dominant	4.91×10^{-09}
28	33889323	34036966	*rs110807315*	Additive	8.47×10^{-12}

				Dominant	5.15×10^{-15}
28	33934171	34132782	*rs137124875*	Recessive	3.65×10^{-08}
29	24027001	24271914	*rs42464845*	Additive<br>Recessive	4.11×10^{-08}<br>3.16×10^{-08}
29	31383123	31927372	*rs132973292*	Dominant	8.41×10^{-09}
29	34312116	34888053	*rs136941415*	Dominant	1.56×10^{-10}
X	111131756	116405891	*rs134545932*	Additive<br>Dominant	1.91×10^{-09}<br>4.64×10^{-10}
X	115494371	121416926	*rs41567540*	Dominant	4.91×10^{-09}
X	127040295	134289022	*rs137331944*	Dominant	1.63×10^{-10}
X	131824609	142115659	*rs134273064**	Additive	1.57×10^{-08}
X	136668779	147800877	*rs136023557*	Additive<br>Dominant	8.06×10^{-11}<br>8.06×10^{-11}

[1]Chromosome location of the locus.
[2]Single nucleotide polymorphism (SNP) location as measured by numbered nucleotides in reference to the ARS 1.2 genome assembly (https://www.animalgenome.org/repository/cattle/UMC_bovine_coordinates/; accessed 19 September 2018).
[3]SNP location as measured by numbered nucleotides in reference to the UMD 3.1 genome assembly (http://bovinegenome.org/?q=node/61; accessed 15 September 2016).
[4]The most significant SNP in the locus associated with heifer conception rate as identified by *rs* number which is a reference number assigned to markers submitted to the National Center for Biotechnology Information SNP database (https://www.ncbi.nlm.nih.gov/projects/SNP/; accessed 9 March 2016).
[5] If SNP have been previously identified to be a part of a copy number variation (CNV) (using UMD 3.1 coordinates) the *rs* number is marked with an “*”.
[6]Genome-wide association model.
[7]Significance (*P*-value) of the most significant SNP associated with heifer conception rate.

Table S2. Loci associated with heifer conception rate to repeated AI services (TBRD).

BTA[1]	ARS 1.2 BP Position[3]	UMD 3.1 BP Position[2]	SNP ID[4,5]	Model[6]	P-Value[7]
UNK	UNK	BTA6 100119868	*rs41594506*	Additive<br>Dominant	6.95×10^{-14}<br>1.92×10^{-14}
UNK	UNK	BTA21 29702780	*rs110995279*	Additive<br>Dominant	3.16×10^{-08}<br>5.52×10^{-09}
1	4253950	3546098	*rs136767715*	Additive<br>Dominant	2.67×10^{-14}<br>1.56×10^{-26}
1	5390376	4654050	*rs42317784**	Recessive	3.21×10^{-08}
1	13869391	13296572	*rs132784449**	Additive<br>Dominant	4.22×10^{-12}<br>1.65×10^{-14}
1	15273288	14740447	*rs109845303*	Recessive	3.55×10^{-14}
1	21885012	21386504	*rs137503737**	Additive<br>Dominant	2.65×10^{-09}<br>4.49×10^{-11}
1	24140382	23652573	*rs136178026*	Recessive	2.89×10^{-10}
1	26449153	25953419	*rs135950336*	Dominant	1.84×10^{-08}
1	32344466	31910495	*rs42869460**	Additive<br>Dominant	4.37×10^{-08}<br>4.23×10^{-09}
1	32895270	32462420	*rs43222212**	Additive<br>Dominant	5.46×10^{-09}<br>2.78×10^{-08}
1	35674365	35264658	*rs134254796*	Dominant	1.80×10^{-08}
1	36708125	36308223	*rs132824212*	Additive<br>Dominant	2.54×10^{-09}<br>1.57×10^{-15}

1	39967296	39584716	*rs133914995*	Additive<br>Dominant	2.67×10^{-08}<br>4.81×10^{-08}
1	43219861	42851166	*rs43734604*	Additive<br>Dominant	3.09×10^{-08}<br>2.03×10^{-11}
1	60732928	61283063	*rs133972911*	Additive<br>Dominant	1.32×10^{-10}<br>8.22×10^{-12}
1	67838260	68436471	*rs110693447**	Additive<br>Dominant	3.12×10^{-09}<br>6.66×10^{-14}
1	69188246	69793194	*rs137088827*	Additive<br>Recessive	1.03×10^{-08}<br>3.57×10^{-17}
1	74094195	74704465	*rs109010777*	Dominant	5.11×10^{-10}
1	80067991	80658583	*rs43249783*	Dominant	4.64×10^{-08}
1	87575778	88168898	*rs42687239*	Additive<br>Dominant<br>Recessive	8.95×10^{-16}<br>8.46×10^{-09}<br>2.85×10^{-12}
1	103622454	104439912	*rs135125978*	Recessive	8.83×10^{-09}
1	*104515879*	UNK	*rs43253279*	Additive<br>Dominant	9.92×10^{-11}<br>1.74×10^{-11}
1	104889259	105711733	*rs135460421*	Additive<br>Dominant	8.62×10^{-09}<br>3.10×10^{-09}
1	126636424	127635326	*rs134321837*	Additive<br>Dominant	4.17×10^{-13}<br>1.24×10^{-13}
1	127553539	128552319	*rs109106945*	Dominant	3.85×10^{-08}
1	130414610	131474130	*rs41986752*	Additive<br>Dominant	2.66×10^{-08}<br>2.73×10^{-08}
1	146156382	147891529	*rs134566710*	Additive<br>Dominant	1.12×10^{-08}<br>4.14×10^{-09}
1	149176285	150881810	*rs134145890*	Dominant	9.08×10^{-10}
1	153973110	155709241	*rs135643439*	Dominant	6.07×10^{-09}
2	693039	619298	*rs133758759*	Additive<br>Dominant	3.48×10^{-08}<br>2.58×10^{-08}
2	5758799	5691695	*rs110306500*	Additive<br>Dominant	1.21×10^{-13}<br>1.96×10^{-15}
2	19157717	19155572	*rs42267687*	Dominant	1.28×10^{-11}
2	32519977	32622594	*rs133040970*	Dominant	9.46×10^{-10}
2	43012494	43122845	*rs43295963*	Additive<br>Dominant	1.29×10^{-11}<br>1.40×10^{-14}
2	53681112	53783708	*rs135006704*	Additive<br>Dominant	4.44×10^{-11}<br>4.41×10^{-12}
2	60562163	60885846	*rs133479876*	Additive	2.85×10^{-09}
2	70452684	70815583	*rs110836558*	Dominant	1.98×10^{-08}
2	71052083	71417750	*rs134623977*	Additive<br>Dominant	1.37×10^{-09}<br>7.84×10^{-13}
2	71825380	72241749	*rs110643377*	Recessive	8.58×10^{-10}
2	85066770	85462609	*rs109342415*	Additive<br>Dominant	3.62×10^{-11}<br>2.90×10^{-15}
2	96352401	96783039	*rs109888113*	Recessive	3.18×10^{-08}

2	97738219	98167527	*rs41641857*	Additive<br>Dominant	1.00×10^{-13}<br>1.93×10^{-18}
2	101319991	101835883	*rs109026677*	Additive<br>Dominant	9.22×10^{-10}<br>3.58×10^{-11}
2	102369444	102886501	*rs110721292*	Additive<br>Recessive	7.90×10^{-09}<br>1.60×10^{-10}
2	102709849	103226604	*rs137445560*	Dominant	1.97×10^{-09}
2	110256790	111014944	*rs137494641*	Additive<br>Dominant	9.38×10^{-11}<br>2.45×10^{-13}
2	112984364	113774680	*rs135273506*	Dominant	2.88×10^{-08}
2	123403424	123947527	*rs110886070**	Dominant	7.26×10^{-09}
2	123641251	124236349	*rs137166038**	Dominant	1.15×10^{-08}
2	126903682	127496020	*rs110838639*	Dominant	2.80×10^{-09}
2	129154800	129751589	*rs136747746**	Dominant	3.00×10^{-11}
2	135499782	136075993	*rs136810779*	Additive<br>Dominant	9.81×10^{-10}<br>6.83×10^{-10}
3	3518128	3547649	*rs109233206*	Additive	1.04×10^{-08}
3	3799465	3837317	*rs134314389*	Dominant	6.55×10^{-09}
3	9380843	9498642	*rs137320425*	Dominant	3.30×10^{-08}
3	15451201	15506128	*rs137471256**	Dominant	4.73×10^{-08}
3	18159642	18198456	*rs134651745*	Dominant	2.24×10^{-10}
3	35668422	35785053	*rs29024428*	Additive<br>Dominant	1.10×10^{-08}<br>5.78×10^{-14}
3	37660099	37781561	*rs137663076*	Additive<br>Recessive	5.61×10^{-09}<br>6.49×10^{-11}
3	38433068	38557710	*rs109265034*	Additive<br>Dominant	7.21×10^{-10}<br>1.94×10^{-18}
3	48845492	49001262	*rs108983995*	Recessive	2.82×10^{-08}
3	64036201	64246703	*rs43342055**	Recessive	1.46×10^{-08}
3	76550807	76976989	*rs42920814**	Additive<br>Dominant	4.25×10^{-11}<br>6.80×10^{-15}
3	80627475	80958341	*rs43353709**	Additive<br>Dominant	1.05×10^{-10}<br>1.43×10^{-11}
3	84669238	85216185	*rs136443501*	Recessive	3.67×10^{-09}
3	84895863	85442743	*rs43272773*	Additive<br>Recessive	1.91×10^{-08}<br>1.58×10^{-11}
3	89840591	90419372	*rs42976322*	Recessive	3.89×10^{-09}
3	90106058	90685311	*rs109308461*	Additive<br>Dominant	2.54×10^{-11}<br>3.16×10^{-12}
3	97504864	98096750	*rs29024665*	Additive<br>Dominant	4.02×10^{-09}<br>3.05×10^{-18}
3	114805862	115411407	*rs110343443**	Dominant	1.97×10^{-09}
3	115082809	115692498	*rs109046293**	Additive<br>Dominant	1.16×10^{-11}<br>1.21×10^{-15}
3	115629791	116253052	*rs133851967**	Additive<br>Dominant	3.64×10^{-11}<br>4.27×10^{-12}

3	120283427	120763623	*rs109717354*	Additive<br>Dominant	2.92×10^{-09}<br>3.51×10^{-10}
4	7814053	7719297	*rs43062157*	Additive<br>Recessive	6.07×10^{-10}<br>3.85×10^{-09}
4	8676114	8500879	*rs136443753*	Dominant	8.46×10^{-09}
4	9354119	9200721	*rs110530879**	Additive<br>Dominant	1.30×10^{-10}<br>8.54×10^{-16}
4	16929574	17923047	*rs109094478*	Additive<br>Dominant	3.22×10^{-08}<br>9.25×10^{-09}
4	22888285	22831648	*rs109672309**	Additive<br>Dominant	9.24×10^{-09}<br>6.72×10^{-13}
4	26010612	25990048	*rs41604485*	Additive<br>Dominant	2.43×10^{-10}<br>1.31×10^{-11}
4	29845133	29947900	*rs43378845*	Dominant	5.39×10^{-09}
4	37056340<br><br>37439721	37235676<br><br>37618902	*rs133417267*<br><br>*rs43388620*	Additive<br>Dominant<br>Recessive	4.23×10^{-21}<br>4.23×10^{-21}<br>2.75×10^{-09}
4	53811395	54127019	*rs43394990*	Additive<br>Dominant	7.37×10^{-13}<br>9.21×10^{-14}
4	70990701	71442761	*rs110114859*	Dominant	1.08×10^{-12}
4	71976781	72430829	*rs135777982*	Recessive	3.98×10^{-10}
4	75427701	76009912	*rs43182769**	Dominant	1.44×10^{-10}
4	91492658	92298886	*rs43407170*	Recessive	1.85×10^{-09}
4	91797929	92603159	*rs29009969*	Recessive	1.17×10^{-08}
4	97484830	98336209	*rs135524891*	Dominant	3.65×10^{-10}
4	116281772	117058812	*rs109741903*	Dominant	1.05×10^{-08}
4	119088045	119886948	*rs137275654*	Additive<br>Dominant	4.22×10^{-08}<br>1.88×10^{-13}
4	119218134	120018401	*rs109409540*	Additive<br>Dominant	6.99×10^{-10}<br>8.24×10^{-11}
5	652247	645386	*rs133539307*	Additive<br>Dominant	7.70×10^{-10}<br>8.07×10^{-15}
5	12363691	12435196	*rs134137475*	Additive<br>Dominant	1.05×10^{-08}<br>2.04×10^{-10}
5	21290477	21357397	*rs137251811*	Dominant	2.38×10^{-09}
5	27683283	27837406	*rs134147281*	Additive<br>Recessive	2.96×10^{-11}<br>6.66×10^{-15}
5	35745274	35925646	*rs135774967*	Additive<br>Recessive	5.18×10^{-11}<br>6.39×10^{-09}
5	37587239	37800654	*rs109295130*	Dominant	4.67×10^{-09}
5	52254169	52496943	*rs41657468*	Additive<br>Dominant	6.85×10^{-16}<br>2.85×10^{-18}
5	62021071	62362183	*rs109867733*	Additive<br>Dominant<br>Recessive	1.22×10^{-16}<br>2.69×10^{-12}<br>2.18×10^{-09}
5	64626266	64968227	*rs133355249*	Dominant	2.41×10^{-08}
5	69286140	69623431	*rs108967945*	Additive	3.62×10^{-15}

				Dominant	3.64×10^{-15}
5	87901217	88311948	*rs43439308*	Additive<br>Dominant	2.50×10^{-09}<br>4.66×10^{-12}
5	88432158	88850154	*rs109772503*	Additive<br>Dominant	1.30×10^{-10}<br>1.53×10^{-16}
5	89543284	89964756	*rs136764099*	Additive<br>Dominant	2.18×10^{-08}<br>2.18×10^{-08}
5	92751411	93180224	*rs134023889*	Recessive	2.44×10^{-10}
5	97645945	98117091	*rs41592957*	Additive<br>Dominant	2.08×10^{-08}<br>3.78×10^{-10}
5	106216381	106708251	*rs133551295*	Recessive	2.16×10^{-08}
5	106786883	107278032	*rs109634884*	Additive<br>Dominant	7.01×10^{-12}<br>1.50×10^{-11}
5	106910293	107407965	*rs133201447*	Additive<br>Dominant	1.06×10^{-12}<br>4.07×10^{-17}
5	107791378	108292341	*rs136136790*	Dominant	1.52×10^{-10}
5	112349683	112869604	*rs109624866*	Dominant	8.51×10^{-09}
5	115195553	115913514	*rs136393747*	Additive<br>Dominant	1.45×10^{-15}<br>8.80×10^{-18}
5	116106908	116834677	*rs133072810*	Additive<br>Dominant	2.55×10^{-10}<br>7.37×10^{-13}
5	117041725	118154242	*rs41593912*	Dominant	1.66×10^{-09}
6	4537028	4501294	*rs135101730*	Dominant	3.30×10^{-08}
6	5785625	6913981	*rs133210289**	Additive	1.81×10^{-08}
6	11385878	12549629	*rs137307365**	Recessive	3.07×10^{-10}
6	11990040	13154909	*rs43450481*	Dominant	4.65×10^{-12}
6	20288492	21570560	*rs137779599*	Additive<br>Dominant	1.55×10^{-12}<br>2.67×10^{-12}
6	21839136	23165378	*rs43097424*	Additive<br>Recessive	9.46×10^{-10}<br>8.25×10^{-13}
6	24944761	26314684	*rs135805690*	Additive<br>Dominant	2.95×10^{-11}<br>2.14×10^{-13}
6	27216433	28637813	*rs109909745*	Dominant	4.22×10^{-08}
6	28927865	30352852	*rs108947176*	Additive<br>Dominant	6.09×10^{-12}<br>5.08×10^{-18}
6	29353493	30779206	*rs109717767*	Dominant	4.50×10^{-08}
6	32319511	33750009	*rs110138532*	Dominant	4.55×10^{-08}
6	33618635	35055841	*rs134948809*	Recessive	1.71×10^{-08}
6	36107083	37533544	*rs110377022*	Additive<br>Dominant	2.34×10^{-08}<br>1.94×10^{-13}
6	42325284	43793054	*rs43465516**	Dominant	8.89×10^{-09}
6	*49162058*	BTA11<br>79425278	*rs42506221*	Dominant	6.33×10^{-09}
6	61819817	63475480	*rs109624509*	Additive	4.71×10^{-09}
6	64697753	66339518	*rs137591215*	Recessive	7.63×10^{-11}
6	71628218	73293138	*rs43467343*	Dominant	2.54×10^{-08}

6	78196637	79865986	*rs43468545*	Additive	4.98×10^{-09}
6	79765417	81489249	*rs135378647**	Additive<br>Dominant	1.05×10^{-11}<br>8.46×10^{-13}
6	88327574	90075383	*rs41870471*	Dominant	4.25×10^{-09}
6	89400297	91146907	*rs42581622*	Additive<br>Dominant	9.39×10^{-11}<br>1.36×10^{-13}
6	91512574	93263835	*rs43476570*	Additive<br>Dominant	2.41×10^{-15}<br>1.38×10^{-21}
6	92698539	94456393	*rs42993521*	Recessive	2.06×10^{-08}
6	96325620	98099748	*rs110349384*	Additive<br>Dominant	8.48×10^{-11}<br>1.09×10^{-16}
6	98103454	99877765	*rs110667921*	Dominant	3.42×10^{-08}
6	107749581	112621102	*rs110068325*	Additive<br>Recessive	1.66×10^{-09}<br>8.26×10^{-09}
7	10109598	10099512	*rs134195407**	Additive<br>Dominant	2.76×10^{-08}<br>4.89×10^{-12}
7	13875817	14998140	*rs133202371*	Additive<br>Recessive	9.24×10^{-11}<br>6.69×10^{-11}
7	20304575	21530928	*rs109558439**	Additive<br>Dominant	9.67×10^{-10}<br>7.87×10^{-15}
7	31508541	32842162	*rs133230493*	Dominant	2.22×10^{-08}
7	42180787	43510537	*rs137054454**	Additive<br>Dominant	5.35×10^{-09}<br>5.92×10^{-11}
7	50956025	52582631	*rs133843787*	Dominant	3.61×10^{-08}
7	52904816	54586502	*rs136035661*	Dominant	3.54×10^{-09}
7	56111544	58055640	*rs133170783*	Additive<br>Dominant	4.58×10^{-13}<br>1.87×10^{-18}
7	59845558	61844595	*rs29021186*	Dominant	3.93×10^{-08}
7	90342277	92696523	*rs109118598*	Dominant	3.67×10^{-08}
7	99814269	102233002	*rs134174053*	Dominant	2.04×10^{-08}
7	109134936	111730542	*rs109635492**	Recessive	3.54×10^{-09}
8	3030778	2898483	*rs134614161*	Additive<br>Dominant	1.45×10^{-09}<br>1.40×10^{-14}
8	13184292	13104883	*rs133345372*	Additive<br>Dominant	2.33×10^{-08}<br>5.71×10^{-13}
8	17854565	17846087	*rs109436465*	Dominant	2.52×10^{-08}
8	18365871	18359485	*rs42807799*	Additive<br>Dominant	7.17×10^{-09}<br>6.40×10^{-11}
8	20500129	20510016	*rs134858319*	Additive<br>Dominant	5.08×10^{-11}<br>2.00×10^{-14}
8	25038304	25048409	*rs42212691*	Dominant	7.42×10^{-09}
8	25111465	25123206	*rs110623317*	Dominant	3.95×10^{-14}
8	25649947	25665062	*rs42207644*	Additive<br>Dominant	3.05×10^{-09}<br>5.79×10^{-13}
8	27285102	27295189	*rs109032832*	Dominant	1.37×10^{-08}
8	36790642	36989371	*rs43550132*	Additive<br>Dominant	3.98×10^{-08}<br>1.98×10^{-08}

8	45340654	45624366	*rs109293388*	Dominant	9.23×10^{-09}
8	45456855	45740396	*rs110505607*	Additive<br>Dominant	8.04×10^{-09}<br>2.00×10^{-09}
8	47177218	47474589	*rs136950125*	Dominant	4.04×10^{-12}
8	53769149	54054285	*rs135532670*	Additive<br>Dominant	4.93×10^{-08}<br>1.96×10^{-11}
8	57193589	57592438	*rs109714363*	Additive<br>Recessive	2.23×10^{-08}<br>2.62×10^{-09}
8	64328957	64791562	*rs42251762*	Dominant	6.16×10^{-09}
8	68728673	69225039	*rs109803892*	Dominant	7.13×10^{-11}
8	82147864	83569006	*rs109389362*	Additive<br>Dominant	9.94×10^{-11}<br>5.15×10^{-13}
8	82885198	84307178	*rs43137599*	Additive<br>Dominant	2.49×10^{-12}<br>5.33×10^{-16}
8	89566392	91095445	*rs136793998*	Recessive	1.50×10^{-11}
8	98652238	100328375	*rs134841549*	Additive<br>Recessive	5.67×10^{-10}<br>2.81×10^{-08}
8	106835109	108601170	*rs43580717*	Additive<br>Dominant	4.69×10^{-13}<br>1.30×10^{-19}
9	5812857	5919500	*rs133149639**	Additive<br>Dominant	7.60×10^{-12}<br>1.04×10^{-14}
9	7666665	7878713	*rs133842228*	Recessive	2.85×10^{-08}
9	7796589	8008206	*rs110533598*	Additive<br>Dominant	4.85×10^{-08}<br>1.13×10^{-10}
9	14005315	14216589	*rs41660114*	Additive<br>Dominant	2.81×10^{-09}<br>4.60×10^{-09}
9	15868930	16069409	*rs134329558*	Additive	3.65×10^{-08}
9	17803245	18022184	*rs135175889*	Additive<br>Dominant	1.19×10^{-10}<br>2.67×10^{-12}
9	17804516	18023455	*rs136675875*	Recessive	2.78×10^{-08}
9	24148731	24506687	*rs42231490*	Dominant	4.78×10^{-08}
9	27571945	27942275	*rs135322296*	Dominant	1.70×10^{-09}
9	31949995	32366076	*rs109684479*	Additive<br>Dominant	9.16×10^{-09}<br>7.35×10^{-10}
9	32654991	33073826	*rs137773242*	Recessive	3.48×10^{-08}
9	37734137	38199983	*rs134755337*	Recessive	4.95×10^{-11}
9	51946932	52678477	*rs42330916*	Additive<br>Dominant	6.75×10^{-10}<br>4.13×10^{-12}
9	63284149	64180220	*rs42756701*	Additive<br>Dominant	2.03×10^{-08}<br>9.72×10^{-12}
9	64389223	65306072	*rs43602336*	Additive<br>Dominant	1.79×10^{-13}<br>8.22×10^{-14}
9	68537996	69513582	*rs109605462*	Dominant	9.83×10^{-10}
9	81844949	82950074	*rs43607227*	Dominant	1.18×10^{-08}
9	93002229	94432227	*rs110860938*	Dominant	3.78×10^{-09}
10	2777481	2721649	*rs109139550*	Recessive	2.59×10^{-08}

10	5945561	5873494	*rs136951673*	Dominant	1.76×10^{-08}
10	9782710	9748629	*rs110638547*	Dominant	3.03×10^{-09}
10	9898755	9864676	*rs134970914*	Additive<br>Dominant	4.66×10^{-10}<br>1.22×10^{-09}
10	18579855	18557936	*rs133730050*	Dominant	9.18×10^{-11}
10	20950434	20784448	*rs797745899*	Additive<br>Dominant	1.82×10^{-15}<br>6.20×10^{-21}
10	35290328	35397418	*rs134282076*	Additive<br>Dominant	2.35×10^{-10}<br>1.17×10^{-11}
10	36309874	36422618	*rs519392213*	Dominant	8.28×10^{-09}
10	37088125	37205266	*rs135421839*	Additive<br>Dominant	1.19×10^{-17}<br>1.71×10^{-19}
10	40344035	40422203	*rs110068388*	Additive	2.71×10^{-10}
10	40517970	40596210	*rs43619838*	Dominant	4.08×10^{-14}
10	55502976	55575425	*rs135035943*	Dominant	2.53×10^{-08}
10	55542744	55615067	*rs135743405*	Additive<br>Dominant	1.52×10^{-09}<br>1.78×10^{-10}
10	59695958	59882200	*rs110617366**	Additive<br>Dominant	1.17×10^{-20}<br>1.57×10^{-27}
10	65704210	65934611	*rs137047345*	Additive<br>Dominant	2.98×10^{-11}<br>1.54×10^{-11}
10	72003636	72249788	*rs133399594*	Dominant	5.69×10^{-09}
10	80769155	81119788	*rs42958443*	Dominant	2.29×10^{-10}
10	84004823	84382827	*rs135037566*	Additive<br>Dominant	5.45×10^{-13}<br>9.76×10^{-17}
10	88298292	89365394	*rs109484520*	Dominant	1.78×10^{-09}
10	89646890	90725565	*rs43650269*	Dominant	4.38×10^{-09}
10	90161552	91261309	*rs42444849*	Dominant	3.70×10^{-10}
10	96305589	97382623	*rs110432233*	Additive<br>Dominant	2.01×10^{-10}<br>2.10×10^{-13}
10	98022135	99101604	*rs109836144*	Additive<br>Dominant	3.23×10^{-11}<br>9.65×10^{-16}
11	2159226	2125437	*rs133812771*	Additive<br>Dominant	2.15×10^{-13}<br>8.61×10^{-23}
11	3394978	3362006	*rs43661827*	Additive<br>Dominant	6.17×10^{-09}<br>2.52×10^{-09}
11	3603226	3571871	*rs109732686*	Dominant	2.08×10^{-09}
11	8208707	8199637	*rs43659348*	Recessive	2.88×10^{-10}
11	14248999	14260840	*rs135753904**	Additive<br>Dominant	3.43×10^{-09}<br>9.58×10^{-10}
11	16856558	16878438	*rs109265815*	Additive<br>Dominant	1.40×10^{-08}<br>6.02×10^{-11}
11	23805191	23867860	*rs42494516**	Additive<br>Dominant	4.06×10^{-11}<br>3.96×10^{-17}
11	24488694	24553769	*rs137596467*	Recessive	7.39×10^{-09}
11	34023541	33857688	*rs108947703**	Additive<br>Dominant	4.99×10^{-10}<br>1.66×10^{-09}

11	54294891	54218975	*rs109845691*	Dominant	2.19×10^{-08}
11	66697709	66672407	*rs109684663*	Additive<br>Dominant	4.69×10^{-09}<br>2.24×10^{-10}
11	75173290	75222305	*rs109611855**	Additive<br>Dominant	1.27×10^{-10}<br>1.88×10^{-13}
11	86434490	86400864	*rs110541854*	Additive<br>Dominant	3.93×10^{-12}<br>1.29×10^{-19}
11	103826584	103878529	*rs132699263**	Dominant	9.72×10^{-09}
11	106385151	105731694	*rs134749626*	Additive<br>Dominant	1.31×10^{-12}<br>5.55×10^{-13}
12	1482941	1421632	*rs110192562*	Additive<br>Dominant	3.23×10^{-08}<br>9.09×10^{-10}
12	15745572	15795775	*rs109236782*	Additive	4.36×10^{-09}
12	19794783	19889431	*rs133053197*	Dominant	6.52×10^{-12}
12	22522443	22541000	*rs137091747*	Additive<br>Dominant	4.04×10^{-12}<br>5.41×10^{-15}
12	26581859	26607578	*rs41623530*	Additive	3.29×10^{-08}
12	41331602	41618712	*rs109582977*	Dominant	1.89×10^{-08}
12	45305543	45608128	*rs134957168**	Additive<br>Dominant	1.95×10^{-11}<br>1.49×10^{-12}
12	46724096	47032066	*rs132902673*	Dominant	1.79×10^{-08}
12	49892078	50238938	*rs136416703*	Recessive	4.40×10^{-08}
12	50157779	50503658	*rs134603164*	Dominant	1.58×10^{-09}
12	54725843	55071355	*rs110859903*	Additive<br>Dominant	3.62×10^{-08}<br>9.15×10^{-09}
12	63170379	62737864	*rs42629844*	Additive<br>Dominant	9.52×10^{-10}<br>3.99×10^{-10}
12	74729747	78603323	*rs109163624**	Additive<br>Dominant	8.86×10^{-11}<br>1.08×10^{-14}
12	74894376	78821023	*rs108947709**	Dominant	1.54×10^{-09}
13	3874215	3765075	*rs42580316*	Additive	2.25×10^{-08}
13	20548292	20828712	*rs109545965**	Additive<br>Dominant	4.79×10^{-11}<br>4.13×10^{-16}
13	21464768	21747463	*rs135877047*	Additive<br>Dominant	6.12×10^{-10}<br>1.22×10^{-13}
13	25085213	25356593	*rs134588643*	Additive<br>Dominant	1.67×10^{-10}<br>3.72×10^{-13}
13	31629599	31920795	*rs109574513*	Additive<br>Dominant	1.70×10^{-13}<br>1.53×10^{-22}
13	42741708	43152577	*rs110687098*	Additive<br>Dominant	1.99×10^{-08}<br>1.38×10^{-08}
13	45091931	45484656	*rs42628484*	Additive<br>Dominant	8.07×10^{-10}<br>5.75×10^{-11}
13	76130450	76853627	*rs109101250*	Additive<br>Dominant	4.29×10^{-08}<br>9.36×10^{-11}
13	76699693	77423875	*rs137366058*	Dominant	8.48×10^{-11}
13	83059172	83523712	*rs43114788*	Additive	5.98×10^{-09}

13	82753235	83832617	*rs109405072*	Recessive	4.01×10^{-08}
UNK	UNK	BTA14<br>593705	*rs109988691**	Additive<br>Dominant	6.86×10^{-10}<br>5.44×10^{-11}
14	676575	1868636	*rs133119726*	Dominant	3.42×10^{-08}
14	18134618	19745134	*rs42663230*	Dominant	3.48×10^{-08}
14	31238198	33253779	*rs109463555*	Dominant	4.53×10^{-08}
14	47975068	50136890	*rs110882087*	Additive<br>Dominant	5.38×10^{-11}<br>2.08×10^{-12}
14	48773447	50934575	*rs132818847**	Dominant	3.01×10^{-11}
14	54380861	56545121	*rs110493329*	Recessive	2.42×10^{-08}
14	55330368	57492008	*rs110257684*	Dominant	1.61×10^{-09}
14	76531942	78909801	*rs134190366*	Additive<br>Dominant	8.16×10^{-10}<br>2.04×10^{-16}
14	79129931	81502666	*rs136688439*	Recessive	4.61×10^{-08}
15	13118554	13386488	*rs136681359*	Recessive	6.13×10^{-09}
15	18922818	19211540	*rs108940684**	Dominant	3.47×10^{-09}
15	23184853	23519871	*rs41750506*	Recessive	3.82×10^{-08}
15	26169835	26550309	*rs135503279*	Dominant	4.41×10^{-13}
15	29019426	29456412	*rs134968275*	Dominant	1.55×10^{-08}
15	33634085	34171356	*rs110805877*	Dominant	2.05×10^{-09}
15	41599198	42161747	*rs136550796*	Dominant	2.91×10^{-08}
15	46515608	47135215	*rs109396578*	Additive<br>Dominant	3.16×10^{-12}<br>7.01×10^{-13}
15	50074380	49602802	*rs135572576**	Additive<br>Dominant	4.07×10^{-09}<br>4.07×10^{-09}
15	53990984	54767938	*rs133256948*	Dominant	8.58×10^{-09}
15	72865236	73737971	*rs132761388*	Dominant	1.60×10^{-09}
15	76155452	77029372	*rs41779939*	Dominant	2.20×10^{-08}
15	81269071	82625640	*rs135646298*	Dominant	5.42×10^{-11}
15	81405698	82762748	*rs134831575*	Dominant	3.85×10^{-08}
15	81732055	83087960	*rs136991739**	Dominant	2.03×10^{-08}
16	762243	561602	*rs133959359*	Recessive	2.81×10^{-08}
16	4272804	4123503	*rs41795035*	Dominant	1.15×10^{-08}
16	5730798	5581530	*rs43170110**	Additive<br>Recessive	7.43×10^{-09}<br>5.40×10^{-09}
16	8394713	8983892	*rs42722094**	Additive<br>Dominant	6.99×10^{-10}<br>2.86×10^{-10}
16	13023901	13642631	*rs110520766*	Additive<br>Dominant	3.18×10^{-08}<br>2.72×10^{-12}
16	13821123	14448472	*rs382568982*	Dominant	8.84×10^{-09}
16	15837070	16473798	*rs133881641*	Dominant	1.88×10^{-12}
16	16552852	17192121	*rs136621576*	Additive<br>Dominant	5.66×10^{-11}<br>5.50×10^{-10}
16	19514414	20201093	*rs133666408*	Additive<br>Dominant	2.93×10^{-09}<br>4.79×10^{-10}

16	21156409	21841998	*rs110460121*	Additive<br>Dominant	5.87×10^{-13}<br>3.83×10^{-16}
16	27674670	28377383	*rs136339981*	Additive<br>Dominant	3.84×10^{-09}<br>7.50×10^{-11}
16	35699058	36501036	*rs135448881*	Additive<br>Dominant	2.24×10^{-08}<br>1.63×10^{-10}
16	35785624	36598644	*rs133215618*	Dominant	9.93×10^{-09}
16	51275917	52474552	*rs109265376*	Additive<br>Dominant	9.66×10^{-11}<br>5.83×10^{-09}
16	56379164	57841028	*rs136790754*	Additive<br>Dominant	1.99×10^{-08}<br>2.12×10^{-09}
16	56683192	58141147	*rs110494034*	Dominant	2.32×10^{-10}
16	66828671	68316257	*rs136391666*	Additive<br>Dominant	2.26×10^{-08}<br>2.73×10^{-08}
16	74989779	76893915	*rs41971371*	Dominant	2.03×10^{-08}
16	75982106	77884389	*rs132824774*	Dominant	8.69×10^{-09}
16	76545440	78465065	*rs42426546*	Additive<br>Recessive	1.18×10^{-09}<br>7.39×10^{-13}
16	78779992	80702862	*rs41825612*	Additive<br>Dominant	1.20×10^{-08}<br>2.31×10^{-14}
17	3604675	3595026	*rs109750106*	Additive<br>Dominant	1.22×10^{-10}<br>1.41×10^{-18}
17	5614491	5596017	*rs132777965*	Additive<br>Dominant	6.26×10^{-17}<br>8.36×10^{-17}
17	5954111	5942207	*rs132686553*	Dominant	1.22×10^{-08}
17	11393665	11543183	*rs110144635**	Dominant	3.81×10^{-10}
17	12688336	12853755	*rs41835005*	Dominant	5.36×10^{-10}
17	13857496	14035805	*rs42423855*	Additive<br>Dominant	2.38×10^{-08}<br>1.12×10^{-09}
17	23212312	23537464	*rs134360207*	Dominant	4.50×10^{-11}
17	24539182	24864639	*rs110174173*	Recessive	2.01×10^{-12}
17	27878822	28295990	*rs109063837*	Additive<br>Dominant	3.79×10^{-14}<br>8.55×10^{-19}
17	44605422	45594042	*rs41845225*	Additive	4.70×10^{-08}
17	51975897	54223575	*rs134740362*	Dominant	2.37×10^{-08}
17	52074637	54321728	*rs136973422*	Dominant	1.06×10^{-14}
17	58628136	60885707	*rs135699541*	Additive<br>Dominant	3.29×10^{-12}<br>1.64×10^{-18}
17	63557403	65811553	*rs41854091*	Additive<br>Dominant	1.08×10^{-13}<br>2.09×10^{-20}
17	66079314	68333768	*rs42427669*	Dominant	1.99×10^{-08}
17	66491886	68751224	*rs42277546*	Additive<br>Dominant<br>Recessive	3.42×10^{-14}<br>6.59×10^{-09}<br>6.87×10^{-10}
17	71000696	73020606	*rs109297860**	Additive	3.42×10^{-08}
17	72726732	74741425	*rs135830763**	Dominant	4.27×10^{-09}

17	73036168	75073522	*rs134240868*	Dominant	1.20×10^{-09}
18	21851964	21929721	*rs41872094*	Additive<br>Dominant	2.15×10^{-19}<br>6.27×10^{-29}
18	36346003	36472456	*rs135121781*	Dominant	1.89×10^{-08}
18	40411349	40549802	*rs41871380*	Additive	9.40×10^{-09}
18	41653136	41795500	*rs41878442*	Dominant	1.89×10^{-09}
18	47455514	47659419	*rs133320300*	Recessive	4.44×10^{-08}
18	47702394	47911084	*rs137310621*	Dominant	5.36×10^{-09}
18	51171887	51506915	*rs110113404*	Dominant	2.19×10^{-09}
18	62525015	62851490	*rs133474771**	Dominant	8.04×10^{-09}
18	65793776	65978584	*rs109748813**	Dominant	9.62×10^{-09}
19	3088418	3140555	*rs111018952**	Additive<br>Dominant	4.05×10^{-11}<br>1.31×10^{-15}
19	7351312	7561956	*rs109543948*	Dominant	8.53×10^{-11}
19	21820203	22388808	*rs109724289*	Dominant	6.61×10^{-10}
19	22830266	23401132	*rs110273778*	Additive<br>Dominant	2.77×10^{-08}<br>1.17×10^{-08}
19	24613963	25226055	*rs29013747*	Additive	1.35×10^{-09}
19	37394376	38028013	*rs135474456*	Dominant	2.98×10^{-09}
19	45692847	46332530	*rs109490677*	Dominant	4.29×10^{-08}
19	47668260	48316618	*rs136268631*	Dominant	4.22×10^{-10}
19	49076932	49695304	*rs133813917*	Additive<br>Dominant	4.28×10^{-09}<br>5.98×10^{-14}
19	50303597	50943903	*rs110015996**	Additive<br>Dominant	3.65×10^{-09}<br>1.17×10^{-10}
19	51234762	51843937	*rs110849586*	Dominant	3.80×10^{-08}
19	52826593	53443757	*rs110291598*	Additive<br>Dominant	2.70×10^{-09}<br>3.67×10^{-14}
19	56473186	57094538	*rs135390224*	Dominant	2.58×10^{-08}
19	59389895	59961830	*rs137305367*	Dominant	6.36×10^{-09}
20	6297268	6213100	*rs42638968**	Dominant	2.42×10^{-08}
20	7233587	7148044	*rs133098433*	Additive<br>Dominant	2.82×10^{-12}<br>1.13×10^{-16}
20	11470424	11400598	*rs42590241*	Recessive	5.15×10^{-09}
20	12685472	12629242	*rs42242736*	Additive<br>Dominant	3.12×10^{-09}<br>5.11×10^{-10}
20	19508245	19511422	*rs110575636*	Dominant	1.41×10^{-08}
20	27079454	27092889	*rs109295451**	Dominant	3.02×10^{-08}
20	28540989	28553271	*rs136316656*	Additive<br>Dominant	3.14×10^{-15}<br>1.26×10^{-19}
20	31034452	31051302	*rs41567173*	Dominant	3.52×10^{-10}
20	35856280	35874119	*rs109846657*	Additive<br>Dominant	9.22×10^{-16}<br>1.26×10^{-18}
20	36625180	36641839	*rs133566169*	Dominant	2.62×10^{-08}
20	48979314	49041839	*rs109224643*	Additive	9.49×10^{-10}

				Dominant	8.45×10^{-11}
20	50708550	50799104	*rs110257354*	Additive<br>Dominant	1.11×10^{-09}<br>9.74×10^{-13}
20	51754819	51811579	*rs135702037**	Additive<br>Dominant	1.27×10^{-08}<br>1.50×10^{-14}
20	57038642	57113135	*rs41956232*	Additive<br>Dominant	3.76×10^{-19}<br>4.81×10^{-26}
20	69389934	69531900	*rs42865571*	Dominant	1.29×10^{-08}
20	69848738	69994109	*rs109895103*	Dominant	4.34×10^{-11}
21	20624804	21047254	*rs108985383*	Additive	1.33×10^{-08}
21	35093858	35481751	*rs109950495*	Dominant	1.47×10^{-08}
21	36935644	37330567	*rs137361228*	Dominant	1.10×10^{-09}
21	49135362	49587929	*rs133394428*	Dominant	1.37×10^{-12}
21	53895321	54385593	*rs132953389**	Additive<br>Recessive	9.68×10^{-10}<br>1.15×10^{-11}
21	54515752	54994332	*rs136941405*	Additive<br>Dominant	4.25×10^{-14}<br>6.27×10^{-21}
21	59084030	59587213	*rs109847984*	Recessive	6.82×10^{-12}
21	68574792	70226760	*rs134541128**	Additive<br>Dominant	4.78×10^{-12}<br>4.89×10^{-20}
22	25751908	25864314	*rs136479407*	Additive<br>Dominant	3.02×10^{-09}<br>1.19×10^{-11}
22	34038154	34155564	*rs136054280*	Recessive	4.44×10^{-09}
22	34308443	34424666	*rs133212659*	Additive	7.66×10^{-10}
22	49439358	50005782	*rs134381227*	Recessive	4.94×10^{-14}
23	8682465	8627994	*rs43706099**	Additive<br>Dominant	4.40×10^{-11}<br>3.37×10^{-16}
23	17235589	17209231	*rs133988650*	Additive<br>Dominant	1.74×10^{-11}<br>1.36×10^{-17}
23	28191377	27994640	*rs109111192**	Dominant	4.65×10^{-10}
23	50434331	50299482	*rs109396135*	Dominant	1.83×10^{-10}
24	9496604	9791008	*rs133693422*	Additive<br>Dominant	6.47×10^{-12}<br>4.00×10^{-14}
24	16026309	16334958	*rs135817307*	Additive<br>Dominant	4.89×10^{-09}<br>2.38×10^{-08}
24	27958402	28294938	*rs135215313*	Additive<br>Dominant	1.02×10^{-10}<br>3.07×10^{-11}
24	50038623	50503943	*rs110770205*	Recessive	3.46×10^{-16}
24	56468941	56978656	*rs133992877*	Dominant	1.79×10^{-08}
24	59529618	60015982	*rs136828522*	Dominant	3.71×10^{-08}
25	777406	776266	*rs109635988**	Dominant	1.65×10^{-09}
25	4151257	4169181	*rs110089590**	Additive<br>Recessive	6.10×10^{-13}<br>1.76×10^{-14}
25	6064677	6110390	*rs109096703*	Dominant	3.65×10^{-09}
25	10520959	10582476	*rs135367380*	Dominant	2.40×10^{-08}
25	11861787	11932191	*rs109087355*	Additive	1.45×10^{-11}

				Dominant	1.65×10^{-14}
25	15996288	16101786	*rs133945325*	Additive<br>Dominant	1.03×10^{-10}<br>5.24×10^{-13}
25	30946650	31330584	*rs109893741*	Dominant	1.01×10^{-10}
25	31752763	32144385	*rs42652733*	Additive<br>Dominant	1.30×10^{-08}<br>4.86×10^{-08}
25	40776015	41324679	*rs135744692**	Additive<br>Dominant	5.95×10^{-09}<br>2.33×10^{-12}
25	41033389	41587832	*rs109804681*	Dominant	9.78×10^{-12}
26	11615909	11649512	*rs137571018*	Additive<br>Dominant	9.86×10^{-10}<br>1.55×10^{-10}
26	26715627	26951677	*rs132780515*	Additive<br>Dominant	2.54×10^{-09}<br>1.44×10^{-09}
26	28538139	28800534	*rs42434955*	Additive<br>Dominant	2.66×10^{-10}<br>2.73×10^{-16}
26	32633583	32911434	*rs109276573*	Dominant	7.81×10^{-09}
26	37715873	38049450	*rs42099948*	Dominant	1.45×10^{-08}
26	38623922	38963804	*rs109628657*	Additive<br>Dominant	6.26×10^{-09}<br>6.00×10^{-14}
26	39706475	40049643	*rs109718141*	Additive<br>Dominant	6.05×10^{-10}<br>2.67×10^{-14}
26	48306565	48655615	*rs135528243*	Recessive	2.96×10^{-09}
27	4172300	3038944	*rs109347167*	Dominant	3.25×10^{-08}
27	13504095	12541110	*rs137329427*	Dominant	1.33×10^{-08}
27	14744513	13786127	*rs42116359*	Dominant	3.14×10^{-08}
27	15478930	14534255	*rs136893095*	Dominant	2.27×10^{-10}
27	22307598	21375791	*rs132728892*	Additive<br>Dominant	9.10×10^{-40}<br>9.10×10^{-40}
27	23516317	22594468	*rs43331413*	Additive<br>Dominant	8.96×10^{-14}<br>6.29×10^{-16}
27	25438697	24573909	*rs41646800*	Additive	2.94×10^{-08}
27	30787648	30476702	*rs137616566*	Additive<br>Dominant	7.24×10^{-10}<br>3.74×10^{-11}
27	33788214	33489613	*rs43727845*	Additive<br>Dominant	2.60×10^{-08}<br>4.31×10^{-12}
27	41475841	41350583	*rs135995504*	Dominant	1.52×10^{-10}
28	2204837	1235275	*rs109198594*	Additive	1.93×10^{-08}
28	5038219	5069177	*rs136255831*	Additive<br>Dominant	2.36×10^{-09}<br>4.24×10^{-10}
28	8174955	8219490	*rs42140351*	Additive<br>Dominant	3.90×10^{-09}<br>8.83×10^{-10}
28	10547512	10597188	*rs135678093*	Dominant	9.41×10^{-10}
28	12450076	12543659	*rs109485541*	Dominant	2.19×10^{-08}
28	20263113	20343549	*rs135148286*	Recessive	4.14×10^{-09}
28	25343046	25500185	*rs42590415**	Recessive	6.16×10^{-09}
28	27482166	27641064	*rs29014532*	Dominant	2.49×10^{-08}

28	32066745	32267783	*rs109304328*	Additive	1.22×10^{-09}
28	33889323	34036966	*rs110807315*	Additive<br>Dominant	2.37×10^{-13}<br>1.06×10^{-17}
28	33934171	34132782	*rs137124875*	Recessive	1.27×10^{-10}
28	38607720	38828404	*rs136201750*	Additive<br>Dominant	2.99×10^{-10}<br>1.55×10^{-12}
29	5223633	5208269	*rs42157512**	Additive	2.82×10^{-08}
29	10014259	10079164	*rs136280135*	Additive<br>Dominant	2.86×10^{-08}<br>7.64×10^{-13}
29	14610685	14697645	*rs43733771*	Additive<br>Dominant	2.01×10^{-08}<br>5.16×10^{-10}
29	16474215	16564960	*rs29014436*	Dominant	3.18×10^{-08}
29	18715811	18813247	*rs110602111*	Additive<br>Dominant	2.13×10^{-09}<br>4.14×10^{-19}
29	24027001	24271914	*rs42464845*	Additive<br>Recessive	5.34×10^{-13}<br>1.72×10^{-12}
29	25227903	25478234	*rs136026492*	Dominant	2.77×10^{-08}
29	27779404	28147351	*rs29018127**	Additive<br>Dominant	3.53×10^{-09}<br>7.98×10^{-11}
29	31383123	31927372	*rs132973292*	Additive<br>Dominant	6.52×10^{-12}<br>6.22×10^{-15}
29	33112745	33666349	*rs42179637*	Recessive	4.17×10^{-09}
29	34312116	34888053	*rs136941415*	Dominant	5.82×10^{-12}
29	47373480	48028082	*rs41257289**	Dominant	9.30×10^{-10}
29	50661370	50755666	*rs110525913*	Additive<br>Dominant	1.96×10^{-08}<br>1.36×10^{-12}
X	13139329	13001585	*rs135502386*	Additive<br>Dominant	6.02×10^{-09}<br>8.34×10^{-10}
X	49570595	50978741	*rs137203836**	Additive<br>Dominant	4.87×10^{-10}<br>5.50×10^{-10}
X	64459233	72028840	*rs134529875**	Additive<br>Dominant	1.26×10^{-08}<br>4.91×10^{-11}
X	80695627	85992720	*rs133469168*	Dominant	3.94×10^{-08}
X	101586329	107043386	*rs29017374*	Dominant	7.65×10^{-09}
X	107443346	112733176	*rs132664611*	Additive<br>Dominant	8.09×10^{-09}<br>1.27×10^{-09}
X	111131756	116405891	*rs134545932*	Additive<br>Dominant	2.55×10^{-12}<br>6.24×10^{-13}
X	115494371	121416926	*rs41567540*	Additive<br>Dominant	4.36×10^{-09}<br>6.44×10^{-09}
X	120942475	128056925	*rs137740227*	Dominant	1.14×10^{-09}
X	127040295	134289022	*rs137331944*	Additive<br>Dominant	5.97×10^{-09}<br>1.30×10^{-13}

[1]Chromosome location of the locus.
[2]Single nucleotide polymorphism (SNP) location as measured by numbered nucleotides in reference to the ARS 1.2 genome assembly (https://www.animalgenome.org/repository/cattle/UMC_bovine_coordinates/; accessed 19 September 2018).

[3]SNP location as measured by numbered nucleotides in reference to the UMD 3.1 genome assembly (http://bovinegenome.org/?q=node/61; accessed 15 September 2016).
[4]The most significant SNP in the locus associated with heifer conception rate as identified by *rs* number which is a reference number assigned to markers submitted to the National Center for Biotechnology Information SNP database (https://www.ncbi.nlm.nih.gov/projects/SNP/; accessed 9 March 2016).
[5] If SNP have been previously identified to be a part of a copy number variation (CNV) (using UMD 3.1 coordinates) the *rs* number is marked with an "*".
[6]Genome-wide association model.
[7]Significance (*P*-value) of the most significant SNP associated with heifer conception rate.

Table S3. Master regulators associated with heifer conception rate positional candidate genes.

Regulator	P-value[1]	Depth[2]	# Genes[3]	List of Regulated Positional Candidate Genes
ABL1	3.20 × 10^{-03}	3	101	*ABCC9, ABHD2, ABLIM1, ABR, ACTN1, ADCY5, ADGRV1, AKAP9, ATP6V0A2, ATXN10, CADM2, CAMK2D, CARD9, CCNE1, CCT6A, CD3E, CDC45, CDH10, CHMP2A, CHMP4A, CHRDL2, CLDN5, COPA, CRACR2B, DDHD1, DDR1, DLG2, DPYD, EDIL3, EIF2B1, EPB41L5, EXOC4, FADD, FOXP2, FSCN2, FZD1, GBX2, GNAQ, GPSM1, HMG20B, HPSE, IGSF9B, IPO4, ITGB5, KRT7, LFNG, LMF1, MAD1L1, MAST4, MED13L, MGA, NAV2, NCOA3, NEDD8, NELL2, NLGN4X, P2RX2, PADI2, PCLO, PIDD1, PIK3CB, PIK3R1, PKDCC, PLIN2, PNPLA2, POU2F2, PSPH, PYGB, RASGEF1B, RBL2, RGS7, ROBO1, SEC23IP, SERPINA1, SGCZ, SHROOM3, SLC16A5, SLC22A23, SOX8, ST8SIA1, STEAP3, SUCLG2, TANC2, TEAD4, THRB, THSD4, TIAL1, TMTC2, TNFRSF4, TNMD, TNN, TNR, TRHR, TRPM3, TRPS1, TWF1, UMPS, UNC5C, UTRN, VAV3, XRN1*
ABL1	6.80 × 10^{-03}	2	61	*ABHD2, ABLIM1, ACTN1, ADGRV1, CAMK2D, CCNE1, CCT6A, CD3E, CDC45, CDH10, CHMP4A, CLDN5, COPA, CRACR2B, DDR1, DLG2, EDIL3, EIF2B1, FADD, FZD1, GBX2, GNAQ, HMG20B, HPSE, ITGB5, KRT7, LFNG, MAD1L1, MAST4, MED13L, NAV2, NCOA3, NEDD8, NELL2, NLGN4X, PADI2, PCLO, PIDD1, PIK3CB, PIK3R1, PLIN2, PNPLA2, POU2F2, PYGB, RBL2, RGS7, ROBO1, SEC23IP, SERPINA1, SHROOM3, ST8SIA1, STEAP3, THRB, THSD4, TNFRSF4, TNN, TRHR, UMPS, UTRN, VAV3, XRN1*
Act1	9.80 × 10^{-03}	3	84	*ABHD2, ABR, ADCY5, ADGRV1, ANKRD17, ARSB, ATP6V0A2, CCNE1, CD3D, CD3G, CDC45, CHMP2A, CHRDL2, CNKSR1, CNRIP1, CRACR2B, DPM3, DPYD, EDA, EFNA1, EIF2B1, EPB41L5, FCAR, GALNT2, GCHFR, GDNF, HAS2, HAS3, HERC3, HID1, HPSE, IGSF9B, IKBKE, IRAK4, ITGB5, LFNG, LGR4, MAD1L1, MAST4, MED13L, mir-383, NAV2, NCOA3, NCR1, NEDD8, OSBPL3, P2RX2, PADI2, PCLO, PDE4D, PHF21A, PIDD1, PIK3CB, PIK3R1, PIP5K1C, PLXDC2, POU2F2, PPFIA4, PSPH, PYGB, RASGEF1B, RGS7, RMDN3, RPLP2, SEC23A, SERPINF2, SGCZ, SHROOM3, SLC16A5, SLC25A4, ST8SIA1, STEAP3, THRB, THSD4, TIAM1, TMED2, TNFRSF18, TNMD, TNN, TNR, TRHR, TRPS1, UMPS, VAV3*

Actin	1.50 × 10^{-03}	3	86	*ACTN1, AKAP9, ANO1, ATP13A5, ATP6V0A2, ATXN10, BEND5, CAMK2D, CARD9, CCNE1, CD3E, CD3G, CDH10, CHMP4A, CHRM3, COPA, CRACR2B, DDR1, DLG2, DOCK10, DPYD, EDIL3, EFNA1, EIF2B1, FCAR, FOXM1, FSCN2, GALNT2, GBX2, GDNF, GNAQ, HERC3, HID1, HMG20B, HPSE, IKBKE, IRAK4, ITGB5, LRP6, MAD1L1, MAST4, MED13L, NCOA3, NCR1, NEDD8, NELL2, NLGN4X, PADI2, PDE4D, PDE5A, PFKP, PIDD1, PIK3CB, PIK3R1, PIP5K1C, PKDCC, PPFIA4, PSPH, PYGB, RBFOX1, RBL2, RGS10, RMDN3, ROBO1, RPLP2, SEC23A, SEC23IP, SEMA3E, SHROOM3, ST8SIA1, STEAP3, STXBP6, TEAD4, TENM3, THBS2, THSD4, TJP2, TMED2, TNFRSF18, TNFRSF4, TRHR, TSSK4, TWF1, UMPS, VAV3, XRN1*
Actin	7.40 × 10^{-03}	2	35	*ACTN1, CAMK2D, CCNE1, CDH10, CHRM3, CRACR2B, DDR1, EDIL3, EFNA1, EIF2B1, FOXM1, FSCN2, GBX2, GDNF, HPSE, IKBKE, ITGB5, LRP6, MAD1L1, MED13L, NEDD8, PADI2, PFKP, PIDD1, RBL2, ROBO1, SHROOM3, STEAP3, TEAD4, THBS2, TJP2, TNFRSF18, TNFRSF4, TRHR, UMPS*
AKR1B1	5.40 × 10^{-03}	3	104	*ABCC9, ABR, ACTN1, ADGRV1, ANKRD17, ANO1, AREG, ARSB, ATP13A5, ATP6V0A2, ATXN10, CADM2, CAMK2D, CARD9, CDC45, CDH10, CHMP2A, CHMP4A, CHRDL2, CHRM3, CLDN5, CNKSR1, CNRIP1, COPA, CRACR2B, DDR1, DLG2, DOCK10, DPYD, EDA, EDIL3, EFNA1, EIF2B1, EPB41L5, EXOC4, FADD, FZD1, GALNT2, GBX2, GCC1, GDNF, GNAQ, GPSM1, GRM8, HAS2, HAS3, HID1, HMCN1, HMG20B, HPSE, IGSF9B, IKBKE, IPO4, ITGB5, LMF1, MAST4, MED13L, MGA, mir-383, NCOA3, NEDD8, NELL2, NLGN4X, OSBPL3, P2RX2, PADI2, PDE4D, PFKP, PIDD1, PIK3R1, PLIN2, PLXDC2, PNPLA2, PSPH, PTCH1, RASGEF1B, RBFOX1, RBL2, RGS10, ROBO1, RPLP2, RSL1D1, SEC23IP, SGCZ, SHROOM3, SLC16A5, ST8SIA1, STEAP3, SUCLG2, TANC2, TENM3, THBS2, THSD4, TIAL1, TJP2, TMTC2, TNFRSF18, TNMD, TNN, TNR, TRPS1, TSSK4, UMPS, XRN1*
Aldose Reductase	2.80 × 10^{-03}	3	86	*ABLIM1, ABR, ADCY5, ADGRV1, ANO1, ARSB, ATP13A5, ATP6V0A2, ATXN10, BEND5, CARD9, CCNE1, CCT6A, CD3D, CD3G, CHMP4A, CHRDL2, CHRM3, CNKSR1, COPA, DLG2, DPM3, DPYD, EDA, EFNA1, EPB41L5, FCAR, GALNT2, GDNF, GNAQ, GPSM1, HAS2, HERC3, HID1, HMG20B, HPSE, IGSF9B, IKBKE,*

				IRAK4, MAD1L1, MAST4, mir-383, MZF1, NAV2, NCR1, NELL2, NLGN4X, P2RX2, PCLO, PDE4D, PDE5A, PIK3CB, PLXDC2, POU2F2, PSPH, PYGB, RASGEF1B, RBFOX1, RBL2, RGS10, RGS7, RMDN3, RPLP2, RSL1D1, SEC23IP, SGCZ, SLC16A5, SLC25A4, ST8SIA1, STXBP6, SUCLG2, TEAD4, TENM3, THRB, THSD4, TIAL1, TJP2, TMTC2, TNMD, TNN, TNR, TRHR, TRPS1, TWF1, XRN1, ZNF280B
APC/APC2	4.40×10^{-03}	3	72	*ABR, ADCY5, ADGRV1, ARSB, ATP13A5, ATP6V0D2, CAMK2D, CCNE1, CCT6A, CD3E, CDC45, CDH10, CHMP2A, CLDN5, CRACR2B, DOCK10, EDIL3, EIF2B1, FADD, FZD1, HAS3, HERC3, HID1, HPSE, LFNG, LGR4, LRP6, MAST4, MED13L, MZF1, NAV2, NCOA3, NEDD8, OSBPL3, P2RX2, PADI2, PCLO, PIDD1, PIK3CB, PIK3R1, PLXDC2, POU2F2, PSPH, PTCH1, PYGB, RASGEF1B, RBFOX1, RGS7, RMDN3, ROBO1, SEC23A, SEMA3E, SERPINF1, SHROOM3, SLC16A5, SLC22A23, SLC25A4, ST8SIA1, STEAP3, TEAD4, THRB, TIAM1, TJP2, TMED2, TNFRSF18, TNFRSF4, TNR, TRPS1, TWF1, UMPS, UTRN, VAV3*
APH1A	4.00×10^{-03}	3	75	*ACTN1, ADGRV1, AREG, ATP6V0D2, ATXN10, CAMK2D, CD3E, CD3G, CDC45, CDH10, CRACR2B, DDR1, DPYD, EDA, EDIL3, EFNA1, EIF2B1, FADD, FCAR, FOXM1, FZD1, GALNT2, GBX2, GCHFR, GDNF, HAS2, HAS3, HERC3, HPSE, IRAK4, ITGB5, LGR4, LRP6, MAD1L1, MAST4, MED13L, MZF1, NAV2, NCOA3, NCR1, NEDD8, NELL2, PADI2, PCLO, PFKP, PHF21A, PIDD1, PIK3CB, PNPLA2, POU2F2, PPFIA4, PTCH1, PTPRN2, PYGB, RBFOX1, RBL2, RGS10, RMDN3, ROBO1, RSL1D1, SEC23A, SEMA3E, SERPINF2, SHROOM3, SLC25A4, STEAP3, SUCLG2, THBS2, TIAM1, TNFRSF18, TNN, TRHR, TSSK4, UMPS, UTRN*
APH1B	3.20×10^{-03}	3	76	*ACTN1, ADGRV1, AREG, ATP6V0D2, ATXN10, CAMK2D, CD3E, CD3G, CDC45, CDH10, CRACR2B, DDR1, DPYD, EDA, EDIL3, EFNA1, EIF2B1, FADD, FCAR, FOXM1, FZD1, GALNT2, GBX2, GCHFR, GDNF, HAS2, HAS3, HERC3, HPSE, IRAK4, ITGB5, LFNG, LGR4, LRP6, MAD1L1, MAST4, MED13L, MZF1, NAV2, NCOA3, NCR1, NEDD8, NELL2, PADI2, PCLO, PFKP, PHF21A, PIDD1, PIK3CB, PNPLA2, POU2F2, PPFIA4, PTCH1, PTPRN2, PYGB, RBFOX1, RBL2, RGS10, RMDN3, ROBO1, RSL1D1, SEC23A,*

				SEMA3E, SERPINF2, SHROOM3, SLC25A4, STEAP3, SUCLG2, THBS2, TIAM1, TNFRSF18, TNN, TRHR, TSSK4, UMPS, UTRN
Arf	7.40×10^{-03}	2	28	*ACTN1, AREG, CAMK2D, CDH10, CRACR2B, DDR1, EDIL3, EFNA1, EIF2B1, FOXM1, GBX2, HAS2, ITGB5, MAD1L1, MED13L, NEDD8, PADI2, PFKP, PIDD1, PIP5K1C, PTCH1, RBL2, ROBO1, SHROOM3, STEAP3, THBS2, TNFRSF18, UMPS*
ARHGAP1	9.30×10^{-03}	3	55	*ABHD2, ABR, ADCY5, ATP13A5, ATP6V0A2, CAMK2D, CD3E, CDC45, CHRDL2, CNKSR1, CNRIP1, CRACR2B, DDR1, DPYD, EDA, EIF2B1, FCAR, FSCN2, GDNF, HID1, HPSE, MAD1L1, MED13L, mir-383, NAV2, NCOA3, NCR1, NEDD8, P2RX2, PADI2, PDE4D, PDE5A, PIDD1, PLXDC2, PSPH, PYGB, RBFOX1, ROBO1, RPLP2, SEC23A, SEMA3E, SGCZ, SHROOM3, SLC16A5, ST8SIA1, STEAP3, TENM3, THSD4, TJP2, TMED2, TNFRSF18, TNN, TNR, UMPS, VAV3*
AXIN1	9.90×10^{-03}	2	38	*ACTN1, ADGRV1, CAMK2D, CDH10, CLDN5, CRACR2B, DDR1, EDIL3, EFNA1, EIF2B1, FADD, FOXM1, FZD1, GBX2, GDNF, HAS3, LFNG, LRP6, MAD1L1, MED13L, NEDD8, PADI2, PCLO, PFKP, PIDD1, PLIN2, RBL2, RGS7, ROBO1, SHROOM3, SLC25A4, STEAP3, THBS2, TIAM1, TNFRSF18, TNN, TRHR, UMPS*
BACH2	7.80×10^{-03}	2	28	*ACTN1, AREG, CAMK2D, CDH10, CRACR2B, DDR1, EDIL3, EFNA1, EIF2B1, FOXM1, GBX2, HAS2, ITGB5, MAD1L1, MED13L, NEDD8, PADI2, PFKP, PIDD1, PTCH1, RBL2, ROBO1, SHROOM3, STEAP3, THBS2, TNFRSF18, TNFRSF4, UMPS*
BAK1	9.90×10^{-03}	3	73	*ABHD2, ABR, ADCY5, ADGRV1, ANKRD17, ARSB, ATP13A5, ATP6V0A2, ATXN10, CCNE1, CD3E, CDC45, CHMP2A, CHRDL2, CNRIP1, CRACR2B, DDR1, DPYD, EDA, EDIL3, EFNA1, EIF2B1, FCAR, GDNF, HAS3, HID1, HPSE, IKBKE, IRAK4, LFNG, MAD1L1, MAST4, MED13L, mir-383, MZF1, NAV2, NCOA3, NEDD8, NELL2, P2RX2, PADI2, PCLO, PDE4D, PHF21A, PIDD1, PIK3CB, PIK3R1, PLXDC2, PSPH, PTPRN2, PYGB, RBFOX1, RGS7, ROBO1, RPLP2, SEMA3E, SGCZ, SHROOM3, SLC16A5, SLC25A4, ST8SIA1, STEAP3, SUCLG2, THRB, THSD4, TIAM1, TNFRSF18, TNFRSF4, TNN, TNR, TRHR, UMPS, VAV3*
BARD1	4.70×10^{-03}	3	85	*ABHD2, ABLIM1, ACTN1, ADGRV1, ARSB, ATP6V0D2, ATXN10, CAMK2D, CD3D, CD3E, CD3G, CDH10, CHMP4A, COPA, CRACR2B, DDR1, DLG2, DOCK10, DPYD, EDIL3, EFNA1, EIF2B1, EPB41L5, FCAR, FOXM1, GALNT2, GBX2, GCHFR, GNAQ, HAS2, IGSF9B,*

				IKBKE, IRAK4, ITGB5, KRT7, LFNG, LGR4, LRP6, MAD1L1, MED13L, MZF1, NCOA3, NCR1, NEDD8, NELL2, NLGN4X, OSBPL3, PADI2, PCLO, PFKP, PHF21A, PIDD1, PIK3CB, PLXDC2, PPFIA4, PTPRN2, PYGB, RASGEF1B, RBFOX1, RGS10, RSL1D1, SEC23A, SEC23IP, SEMA3E, SERPINF2, SHROOM3, SLC25A4, STEAP3, SUCLG2, TANC2, THBS2, THRB, THSD4, TJP2, TMTC2, TNFRSF18, TNFRSF4, TNN, TRHR, TRPS1, TSSK4, UMPS, UTRN, VAV3, XRN1
BID	4.20×10^{-03}	3	75	*ABLIM1, ACTN1, ADGRV1, ANKRD17, ARSB, ATP13A5, ATP6V0D2, ATXN10, CCNE1, CCT6A, CHMP2A, CHMP4A, CHRDL2, CNKSR1, COPA, CRACR2B, DDR1, DLG2, DOCK10, EDA, EDIL3, EIF2B1, FOXM1, GBX2, GCHFR, GNAQ, HAS3, HERC3, HID1, HMG20B, HPSE, MAD1L1, MAST4, MED13L, MZF1, NAV2, NCR1, NEDD8, NLGN4X, OSBPL3, PADI2, PDE5A, PIDD1, PIK3CB, PIK3R1, PIP5K1C, PKDCC, PLXDC2, PPFIA4, PSPH, PTPRN2, PYGB, RGS7, RSL1D1, SEC23A, SEC23IP, SEMA3E, SERPINF2, SHROOM3, ST8SIA1, STEAP3, SUCLG2, THBS2, THRB, TIAL1, TIAM1, TJP2, TMED2, TNMD, TNN, TRHR, UMPS, UTRN, VAV3, XRN1*
BIRC5	9.90×10^{-03}	3	85	*ABCC9, ABHD2, ABLIM1, ANKRD17, ANO1, AREG, ATP13A5, ATXN10, BEND5, CAMK2D, CARD9, CD3D, CD3E, CDH10, CHMP4A, CNKSR1, COPA, CRACR2B, DDR1, DLG2, DOCK10, DPYD, EDA, EDIL3, EFNA1, EIF2B1, FOXM1, GBX2, GCC1, GCHFR, GNAQ, HERC3, HPSE, IGSF9B, IKBKE, IRAK4, KRT7, MAD1L1, MAST4, MED13L, NAV2, NCOA3, NCR1, NEDD8, NELL2, NLGN4X, PADI2, PDE4D, PFKP, PHF21A, PIDD1, PIK3CB, PPFIA4, PSPH, PTCH1, PTPRN2, PYGB, RASGEF1B, RBL2, RMDN3, ROBO1, SEC23IP, SERPINA1, SHROOM3, SLC16A5, SOX8, STEAP3, STXBP6, TEAD4, TENM3, THBS2, THRB, TIAM1, TJP2, TMED2, TNFRSF18, TNMD, TRHR, TRPS1, TSSK4, TWF1, UMPS, VAV3, XRN1, ZNF280B*
BTK	3.20×10^{-03}	3	100	*ABCC9, ABR, ADCY5, AKAP9, ANKRD17, AREG, ARSB, ATP6V0A2, ATXN10, BEND5, CADM2, CAMK2D, CARD9, CD3D, CD3E, CDC45, CDH10, CHMP2A, CHMP4A, CHRDL2, CLDN5, CNKSR1, CNRIP1, COPA, CRACR2B, DDR1, DLG2, DOCK10, DPYD, EDA, EDIL3, EFNA1, EIF2B1, ENPP6, EXOC4, F13B, FADD, FZD1, GALNT2, GBX2, GCC1, GDNF, GPSM1, HAS2, HAS3, HPSE, IGSF9B, IKBKE, ITGB5, KRT7, LMF1, LRP6, MAD1L1, MAST4, MED13L, MGA, NEDD8, NLGN4X, OSBPL3, P2RX2, PADI2, PIDD1, PIK3R1,*

				PIP5K1C, PKDCC, PLIN2, PLXDC2, POU2F2, PSPH, PTCH1, PYGB, RASGEF1B, RBL2, RGS10, ROBO1, RTN1, SEC23IP, SERPINA1, SGCZ, SHROOM3, SLC16A5, SLC25A4, SLC9B2, SOX8, STEAP3, STXBP6, THSD4, TIAL1, TMTC2, TNFRSF18, TNMD, TNN, TNR, TRHR, TRPS1, TSSK4, TWF1, UMPS, UTRN, XRN1
BUB1B	3.90×10^{-03}	2	30	*ACTN1, ADGRV1, AREG, CAMK2D, CCNE1, CDH10, CRACR2B, DDR1, EDIL3, EFNA1, EIF2B1, FOXM1, GBX2, HAS2, ITGB5, MAD1L1, MED13L, NEDD8, PADI2, PFKP, PIDD1, PTCH1, RBL2, ROBO1, SHROOM3, STEAP3, THBS2, TIAM1, TNFRSF18, UMPS*
butyric acid	2.20×10^{-03}	2	62	*ABHD2, ADGRV1, ANKRD17, ATP13A5, CAMK2D, CARD9, CCNE1, CDC45, CLDN5, CRACR2B, DDR1, DLG2, DOCK10, DPP6, DPYD, EDA, EDIL3, EFNA1, EIF2B1, FADD, FZD1, GBX2, GCHFR, HAS3, HPSE, IKBKE, IRAK4, ITGB5, KRT7, MAD1L1, MED13L, NEDD8, OSBPL3, PADI2, PDE4D, PFKP, PHF21A, PIDD1, PIK3R1, PLIN2, PNPLA2, POU2F2, PPFIA4, RBFOX1, RBL2, RGS10, RGS7, ROBO1, RPLP2, RSL1D1, SERPINA1, SERPINF1, SERPINF2, SHROOM3, ST8SIA1, STEAP3, THRB, TIAM1, TNFRSF18, TNN, UMPS, UTRN*
C1D	6.20×10^{-03}	3	58	*ABHD2, ABLIM1, ACTN1, ADGRV1, AREG, ATXN10, CAMK2D, CCT6A, CDH10, CHMP4A, COPA, CRACR2B, DDR1, DLG2, DOCK10, EDIL3, EIF2B1, GALNT2, GBX2, GCHFR, GDNF, GNAQ, HAS2, HPSE, IKBKE, ITGB5, KRT7, LFNG, MAD1L1, MED13L, NCOA3, NCR1, NEDD8, NELL2, NLGN4X, PADI2, PFKP, PIDD1, PNPLA2, RASGEF1B, RBL2, RGS10, ROBO1, SEC23IP, SEMA3E, SERPINF1, SHROOM3, SLC22A23, STEAP3, THRB, TIAL1, TJP2, TSSK4, TWF1, UMPS, UTRN, VAV3, XRN1*
cAMP-dependent protein kinase	7.70×10^{-03}	2	32	*AREG, ATP13A5, CCNE1, CCT6A, CD3D, CDH10, CLDN5, EDIL3, GPSM1, HAS2, IGSF9B, ITGB5, MZF1, OSBPL3, PCLO, PFKP, PHF21A, PIK3CB, PIK3R1, PLIN2, PLXDC2, PNPLA2, RASGEF1B, RBFOX1, ROBO1, SEMA3E, SERPINA1, SERPINF1, TIAL1, TRPS1, UTRN, VAV3*
CARD11	3.00×10^{-03}	3	81	*ABHD2, ABR, ADCY5, ADGRV1, ANKRD17, ARSB, ATP13A5, ATP6V0A2, CCNE1, CD3E, CD3G, CDC45, CHMP2A, CHRDL2, CNKSR1, CNRIP1, CRACR2B, DDR1, DPM3, DPYD, EDA, EFNA1, EIF2B1, EPB41L5, FCAR, FOXM1, GALNT2, GDNF, HAS2, HERC3, HID1, HPSE, IRAK4, LFNG, MAD1L1, MAST4, MED13L, mir-383, NAV2, NCOA3, NCR1, NEDD8, OSBPL3, P2RX2, PADI2, PCLO,*

				PDE4D, PDE5A, PFKP, PHF21A, PIDD1, PIK3CB, PIK3R1, PLXDC2, POU2F2, PPFIA4, PSPH, PYGB, RBFOX1, RGS7, RMDN3, ROBO1, RPLP2, SERPINF2, SGCZ, SHROOM3, SLC16A5, ST8SIA1, STEAP3, THRB, THSD4, TIAL1, TIAM1, TNFRSF18, TNMD, TNN, TNR, TRHR, TRPM3, UMPS, VAV3
CASP10	8.60×10^{-03}	3	66	*ABHD2, ACTN1, ATXN10, CCNE1, CD3G, CDC45, CDH10, CLDN5, CNKSR1, CRACR2B, DDR1, DPM3, EDA, EDIL3, EFNA1, EIF2B1, EPB41L5, FCAR, GALNT2, GCHFR, GDNF, HAS2, HAS3, HERC3, HPSE, IKBKE, IRAK4, LGR4, LRP6, MAD1L1, MAST4, MED13L, MZF1, NAV2, NCOA3, NEDD8, NELL2, PADI2, PCLO, PIDD1, PIK3R1, PLXDC2, POU2F2, PPFIA4, PTPRN2, RBFOX1, RGS7, RMDN3, ROBO1, SEC23A, SEMA3E, SERPINF1, SERPINF2, SHROOM3, SLC25A4, STEAP3, SUCLG2, THBS2, THRB, TIAM1, TNFRSF18, TNMD, TNN, TRHR, UMPS, UTRN*
CASP12	7.70×10^{-03}	3	45	*ACTN1, ATXN10, CDH10, CLDN5, CRACR2B, DDR1, EDIL3, EIF2B1, FADD, FZD1, GBX2, ITGB5, LGR4, MAD1L1, MAST4, MED13L, MZF1, NAV2, NCR1, NEDD8, NELL2, PADI2, PFKP, PIDD1, PLIN2, PLXDC2, PTPRN2, RBFOX1, RGS7, ROBO1, SEC23A, SERPINA1, SERPINF1, SERPINF2, SHROOM3, SLC25A4, STEAP3, SUCLG2, THBS2, TNFRSF18, TNFRSF4, TNMD, TRHR, UMPS, UTRN*
CCL4	6.00×10^{-03}	2	31	*ACTN1, AREG, ARSB, CARD9, CCNE1, CD3D, CDH10, CLDN5, DOCK10, EDIL3, EFNA1, FADD, FOXM1, GDNF, HAS2, IGSF9B, LRP6, OSBPL3, PIK3CB, PIK3R1, PLXDC2, POU2F2, RASGEF1B, ROBO1, SERPINA1, THBS2, TNFRSF4, TNN, TRPS1, UTRN, VAV3*
CCNB1	5.20×10^{-03}	2	31	*ACTN1, AREG, CAMK2D, CCNE1, CCT6A, CDH10, CRACR2B, DDR1, EDIL3, EFNA1, EIF2B1, GBX2, HAS2, HMG20B, ITGB5, LGR4, MAD1L1, MED13L, NEDD8, PADI2, PCLO, PFKP, PIDD1, PTCH1, RBL2, ROBO1, SHROOM3, STEAP3, THBS2, TNFRSF18, UMPS*
CCND3	5.90×10^{-03}	2	22	*AREG, CCNE1, CD3G, FCAR, FOXM1, FZD1, GBX2, HAS2, HERC3, IRAK4, ITGB5, KRT7, NCOA3, PCLO, RBL2, RMDN3, SEMA3E, SERPINA1, SERPINF1, TEAD4, THRB, TIAM1*
CD180	3.40×10^{-03}	3	87	*ABR, ADCY5, ADGRV1, ANKRD17, ANO1, ATP13A5, ATP6V0A2, CAMK2D, CCNE1, CCT6A, CD3G, CHMP4A, CHRDL2, CNKSR1, CNRIP1, COPA, CRACR2B, DDR1, DLG2, DPYD, EDA, EDIL3, EFNA1, EIF2B1, EPB41L5, FCAR, GALNT2, GCHFR, GDNF, GNAQ, HAS2, HAS3, HERC3, HID1, HPSE, IKBKE, IRAK4, LFNG, LGR4,*

				MAD1L1, MAST4, MED13L, mir-383, NCOA3, NCR1, NEDD8, NELL2, NLGN4X, OSBPL3, P2RX2, PADI2, PCLO, PDE4D, PHF21A, PIDD1, PIK3CB, PIK3R1, PLIN2, PLXDC2, POU2F2, PPFIA4, PSPH, PTCH1, PYGB, RBFOX1, RGS7, RMDN3, ROBO1, RPLP2, RSL1D1, SEC23IP, SGCZ, SHROOM3, SLC16A5, SLC25A4, ST8SIA1, STEAP3, THRB, THSD4, TIAM1, TJP2, TNMD, TNN, TNR, TRHR, UMPS, XRN1
CDC14A	7.80×10^{-03}	2	28	*ACTN1, AREG, CAMK2D, CCNE1, CDH10, CRACR2B, DDR1, EDIL3, EFNA1, EIF2B1, FOXM1, GBX2, HAS2, ITGB5, MAD1L1, MED13L, NEDD8, PADI2, PFKP, PIDD1, PTCH1, RBL2, ROBO1, SHROOM3, STEAP3, THBS2, TNFRSF18, UMPS*
CDC14B	8.00×10^{-03}	2	28	*ACTN1, AREG, CAMK2D, CCNE1, CDH10, CRACR2B, DDR1, EDIL3, EFNA1, EIF2B1, FOXM1, GBX2, HAS2, ITGB5, MAD1L1, MED13L, NEDD8, PADI2, PFKP, PIDD1, PTCH1, RBL2, ROBO1, SHROOM3, STEAP3, THBS2, TNFRSF18, UMPS*
CEBPA	7.90×10^{-03}	1	11	*CD3G, FCAR, FZD1, GBX2, HAS2, ITGB5, KRT7, PLIN2, SEMA3E, SERPINF1, THRB*
CERS2	4.40×10^{-03}	3	70	*ABLIM1, ABR, ADCY5, ADGRV1, ATP6V0A2, CAMK2D, CCNE1, CD3E, CDC45, CDH10, CHMP4A, CHRDL2, CNRIP1, COPA, CRACR2B, DDR1, DLG2, DPYD, EDA, EDIL3, EIF2B1, FCAR, GDNF, GNAQ, HAS3, HID1, HPSE, IRAK4, LFNG, LGR4, MAD1L1, MED13L, mir-383, NAV2, NCOA3, NCR1, NEDD8, NELL2, NLGN4X, P2RX2, PADI2, PCLO, PDE4D, PIDD1, PIK3CB, PIK3R1, PLXDC2, PSPH, PTCH1, PYGB, RGS7, ROBO1, RPLP2, SEC23IP, SEMA3E, SGCZ, SHROOM3, SLC16A5, SLC25A4, ST8SIA1, STEAP3, THRB, THSD4, TJP2, TNFRSF4, TNN, TNR, TRHR, UMPS, XRN1*
CGA	7.00 × 10-04	3	54	*ACTN1, AREG, ARSB, ATP13A5, CAMK2D, CCNE1, CCT6A, CD3D, CDH10, CLDN5, CNRIP1, CRACR2B, DDR1, DOCK10, EDIL3, EFNA1, EIF2B1, GBX2, GDNF, HAS3, HMG20B, IGSF9B, IKBKE, IRAK4, LRP6, MAD1L1, MED13L, NCOA3, NEDD8, OSBPL3, PADI2, PCLO, PDE4D, PFKP, PIDD1, PIK3CB, PIK3R1, PLIN2, PLXDC2, PNPLA2, PSPH, RASGEF1B, RBFOX1, RBL2, ROBO1, SERPINF1, SHROOM3, STEAP3, TNFRSF18, TNFRSF4, TNN, TRPS1, UMPS, VAV3*
CMA1	3.70×10^{-03}	3	90	*ABHD2, ABR, ACTL6A, ACTN1, ADCY5, ADGRV1, ANKRD17, AREG, ARSB, ATP13A5, ATP6V0A2, ATXN10, CAMK2D, CCT6A, CD3G,*

				CHRDL2, CHRM3, CLDN5, CRACR2B, DPYD, EDA, EIF2B1, FADD, FCAR, FZD1, GBX2, GCHFR, GDNF, GRM8, HAS2, HID1, HPSE, IGSF9B, IRAK4, ITGB5, KCNJ8, KRT7, LFNG, LGR4, LMF1, MAD1L1, MAST4, MED13L, mir-383, MZF1, NAV2, NCOA3, NCR1, NEDD8, NELL2, P2RX2, PADI2, PCLO, PDE4D, PFKP, PHF21A, PIDD1, PIK3CB, PIK3R1, PLIN2, PLXDC2, PPFIA4, PYGB, RASGEF1B, RBFOX1, RGS10, RGS7, RPLP2, SEC23A, SERPINA1, SERPINF1, SHROOM3, SLC16A5, SLC25A4, SOX8, ST8SIA1, STEAP3, SUCLG2, THBS2, THRB, THSD4, TIAL1, TIAM1, TJP2, TNFRSF4, TNN, TNR, TRPS1, UMPS, VAV3
Creb	6.60×10^{-03}	2	43	*ACTN1, ADGRV1, AREG, ATP13A5, CAMK2D, CCNE1, CD3D, CDH10, CLDN5, CRACR2B, DDR1, EDIL3, EFNA1, EIF2B1, FOXM1, GBX2, IGSF9B, IRAK4, ITGB5, LFNG, MAD1L1, MED13L, NEDD8, OSBPL3, PADI2, PCLO, PFKP, PIDD1, PLIN2, PLXDC2, PSPH, RASGEF1B, RBFOX1, RBL2, ROBO1, SERPINA1, SERPINF1, SHROOM3, STEAP3, THBS2, TNFRSF18, TRPS1, UMPS*
CRTC1	2.70×10^{-03}	3	78	*ABHD2, ABR, ADCY5, ADGRV1, ANKRD17, ATP6V0A2, CAMK2D, CARD9, CCNE1, CD3D, CD3E, CD3G, CDH10, CHRDL2, CNRIP1, CRACR2B, DDR1, DPYD, EDA, EDIL3, EIF2B1, FCAR, FZD1, GDNF, GNAQ, GPSM1, HAS3, HERC3, HID1, HPSE, IKBKE, IRAK4, KRT7, LFNG, LGR4, MAD1L1, MED13L, mir-383, NAV2, NCOA3, NCR1, NEDD8, P2RX2, PADI2, PCLO, PDE4D, PHF21A, PIDD1, PIK3CB, PIP5K1C, PLXDC2, PSPH, PTCH1, PYGB, RGS7, RMDN3, ROBO1, RPLP2, SEC23A, SEMA3E, SGCZ, SHROOM3, SLC16A5, SLC25A4, ST8SIA1, STEAP3, THRB, THSD4, TIAL1, TIAM1, TMED2, TNFRSF18, TNFRSF4, TNN, TNR, TRHR, UMPS, VAV3*
cytokine receptor	3.60×10^{-03}	3	74	*ABR, ADCY5, ADGRV1, ANKRD17, ANO1, ATP13A5, ATP6V0A2, CARD9, CCT6A, CD3E, CD3G, CHMP4A, CHRDL2, CNKSR1, COPA, CRACR2B, DLG2, DPYD, EDA, EFNA1, EIF2B1, ENPP6, EXOC4, F13B, GALNT2, GCHFR, GDNF, GPSM1, HAS2, HERC3, HID1, HMCN1, HMG20B, IGSF9B, IPO4, MAST4, MED13L, MZF1, NAV2, NCR1, NEDD8, NELL2, NLGN4X, P2RX2, PCLO, PDE4D, PDE5A, PIDD1, PIK3CB, PIP5K1C, PITRM1, PSPH, PYGB, RASGEF1B, RBFOX1, RGS10, RMDN3, RPLP2, RTN1, SEC23A, SEC23IP, SGCZ, SHROOM3, SLC16A5, STEAP3, TENM3, THRB, THSD4, TIAL1, TMED2, TNN, TNR, UMPS, XRN1*

DAXX	7.70×10^{-03}	2	42	*ABHD2, ACTN1, AREG, CAMK2D, CCT6A, CD3G, CDH10, CLDN5, CRACR2B, DDR1, EDIL3, EIF2B1, FCAR, FOXM1, FZD1, GBX2, GDNF, HAS2, HPSE, KRT7, MAD1L1, MED13L,* mir-383*, NCR1, NEDD8, PADI2, PFKP, PIDD1, PLIN2, RBL2, ROBO1, SEMA3E, SERPINF1, SGCZ, SHROOM3, STEAP3, THBS2, THRB, TNFRSF18, TNN, TRHR, UMPS*
DHCR24	2.80×10^{-03}	3	91	*ABLIM1, ABR, ACTN1, ADCY5, ADGRV1, ANKRD17, ANO1, AREG, ATP6V0A2, ATXN10, CAMK2D, CCNE1, CD3D, CDH10, CHMP4A, CHRDL2, CLDN5, CNKSR1, CNRIP1, COPA, CRACR2B, DDR1, DLG2, DPM3, DPYD, EDIL3, EFNA1, EIF2B1, FADD, FOXM1, GALNT2, GBX2, GCHFR, GDNF, GNAQ, HAS2, HERC3, HID1, IGSF9B, ITGB5, LFNG, LMF1, LRP6, MAD1L1, MAST4, MED13L, MGA, NAV2, NEDD8, NLGN4X, P2RX2, PADI2, PDE4D, PDE5A, PFKP, PIDD1, PIK3CB, PIP5K1C, PLXDC2, PPFIA4, PSPH, PTCH1, PTPRN2, RASGEF1B, RBL2, RGS10, RGS6, RMDN3, ROBO1, RPLP2, RSL1D1, SEC23IP, SERPINF2, SGCZ, SHROOM3, SLC16A5, SOX8, ST8SIA1, STEAP3, SUCLG2, THBS2, THSD4, TMED2, TMTC2, TNFRSF18, TNN, TNR, TRPS1, TSSK4, UMPS, XRN1*
DHCR24	3.30×10^{-03}	2	34	*ACTN1, AREG, CAMK2D, CCNE1, CDH10, CLDN5, CRACR2B, DDR1, EDIL3, EFNA1, EIF2B1, FADD, FOXM1, GBX2, GDNF, HAS2, ITGB5, LRP6, MAD1L1, MED13L, NEDD8, PADI2, PFKP, PIDD1, PIK3CB, PTCH1, RBL2, ROBO1, SHROOM3, STEAP3, THBS2, TNFRSF18, TNN, UMPS*
DNMT1	4.20×10^{-03}	3	83	*ABHD2, ABLIM1, ADGRV1, ANKRD17, ANO1, AREG, ARSB, ATP13A5, BEND5, CCNE1, CD3D, CD3E, CHMP2A, CHMP4A, CHRDL2, CNKSR1, COPA, DLG2, DOCK10, EDA, EFNA1, EPB41L5, FCAR, GCHFR, GNAQ, HAS2, HAS3, HERC3, HID1, HMG20B, HPSE, IGSF9B, IKBKE, ITGB5, KRT7, LRP6, MAST4,* mir-383*, MZF1, NAV2, NCOA3, NELL2, NLGN4X, OSBPL3, PCLO, PDE5A, PIK3CB, PIK3R1, PIP5K1C, PKDCC, PLXDC2, PPFIA4, PSPH, PTCH1, PYGB, RASGEF1B, RBFOX1, RBL2, RGS10, ROBO1, SEC23A, SEC23IP, SEMA3E, SLC25A4, SLC9B2, SOX8, STXBP6, SUCLG2, TANC2, THBS2, THRB, THSD4, TIAM1, TJP2, TMED2, TNFRSF18, TNFRSF4, TRHR, TRPS1, TWF1, UTRN, VAV3, XRN1*
DNMT1	6.00×10^{-03}	2	29	*ABHD2, ABLIM1, AREG, CCNE1, CHMP4A, COPA, DLG2, EFNA1, GNAQ, HAS2, IKBKE, ITGB5, KRT7, NCOA3, NELL2, NLGN4X,*

				PTCH1, RBL2, RGS10, ROBO1, SEC23IP, SLC25A4, THBS2, THRB, TJP2, TNFRSF18, UTRN, VAV3, XRN1
DR4/5	9.80×10^{-03}	3	72	*ABHD2, ABR, ADCY5, ADGRV1, ANKRD17, ATP6V0A2, CAMK2D, CCNE1, CD3E, CDC45, CDH10, CHRDL2, CNRIP1, CRACR2B, DDR1, EDA, EDIL3, EIF2B1, FCAR, GDNF, HAS3, HID1, HPSE, IKBKE, IRAK4, KRT7, LFNG, LGR4, MAD1L1, MED13L, mir-383, NAV2, NCOA3, NCR1, NEDD8, P2RX2, PADI2, PCLO, PDE4D, PHF21A, PIDD1, PIK3CB, PIK3R1, PIP5K1C, PLXDC2, PSPH, PTCH1, PYGB, RGS7, ROBO1, RPLP2, SEC23A, SEMA3E, SERPINF2, SGCZ, SHROOM3, SLC16A5, SLC25A4, ST8SIA1, STEAP3, THRB, THSD4, TIAM1, TMED2, TNFRSF18, TNFRSF4, TNMD, TNN, TNR, TRHR, UMPS, VAV3*
DUSP10	3.80×10^{-03}	2	33	*ACTN1, AREG, CAMK2D, CCNE1, CDH10, CRACR2B, DDR1, EDIL3, EFNA1, EIF2B1, FOXM1, GBX2, GDNF, HAS2, ITGB5, LRP6, MAD1L1, MED13L, NEDD8, PADI2, PFKP, PIDD1, PLIN2, PTCH1, RBL2, ROBO1, SHROOM3, STEAP3, THBS2, TNFRSF18, TNN, TRHR, UMPS*
DYNLL1	7.30×10^{-03}	3	92	*ABHD2, ABLIM1, ABR, ACTN1, ADCY5, ADGRV1, ANKRD17, ANO1, ARSB, ATP13A5, ATP6V0A2, ATXN10, CCNE1, CCT6A, CD3E, CD3G, CDC45, CHMP4A, CHRDL2, CNKSR1, CNRIP1, COPA, CRACR2B, DDR1, DLG2, DPYD, EFNA1, EIF2B1, EPB41L5, FCAR, GALNT2, GCHFR, GNAQ, HAS2, HAS3, HERC3, HID1, HPSE, IKBKE, IRAK4, KRT7, LFNG, LGR4, MAD1L1, MED13L, mir-383, MZF1, NCOA3, NEDD8, NELL2, NLGN4X, P2RX2, PADI2, PDE4D, PDE5A, PHF21A, PIDD1, PIK3R1, POU2F2, PPFIA4, PSPH, PTCH1, PTPRN2, PYGB, RBFOX1, RBL2, RGS7, RMDN3, ROBO1, SEC23IP, SERPINF2, SGCZ, SHROOM3, SLC16A5, SLC25A4, STEAP3, SUCLG2, TANC2, TENM3, THRB, THSD4, TIAL1, TIAM1, TJP2, TNFRSF18, TNMD, TNN, TNR, UMPS, UTRN, VAV3, XRN1*
DYRK2	8.90×10^{-03}	2	29	*ACTN1, AREG, ATP6V0D2, CAMK2D, CDH10, CRACR2B, DDR1, EDIL3, EFNA1, EIF2B1, FOXM1, GBX2, HAS2, ITGB5, MAD1L1, MED13L, NEDD8, PADI2, PCLO, PFKP, PIDD1, PTCH1, RBL2, ROBO1, SHROOM3, STEAP3, THBS2, TNFRSF18, UMPS*
E2F3	3.00×10^{-04}	3	87	*ABR, ACTN1, ADCY5, ANO1, AREG, ARSB, ATP13A5, ATP6V0A2, ATXN10, BEND5, CAMK2D, CCNE1, CD3D, CD3E, CDC45, CDH10, CHRDL2, CNKSR1, CNRIP1, CRACR2B, DDR1, EDIL3, EFNA1,*

				EIF2B1, FOXM1, GALNT2, GBX2, GCHFR, GDNF, HAS2, HERC3, HID1, IGSF9B, ITGB5, MAD1L1, MED13L, mir-383, MZF1, NAV2, NCOA3, NCR1, NEDD8, NELL2, P2RX2, PADI2, PDE4D, PDE5A, PFKP, PIDD1, PIK3CB, PIK3R1, PLXDC2, PPFIA4, PSPH, PTCH1, PYGB, RASGEF1B, RBFOX1, RBL2, RGS10, RGS7, RMDN3, ROBO1, RPLP2, SEC23A, SGCZ, SHROOM3, SLC16A5, ST8SIA1, STEAP3, STXBP6, SUCLG2, TEAD4, THBS2, THSD4, TMED2, TNFRSF18, TNFRSF4, TNN, TNR, TRHR, TRPS1, TSSK4, TWF1, UMPS, VAV3, ZNF280B
E2F6	8.70×10^{-03}	1	3	*CCNE1, CDC45, SMC1B*
EDAR	8.10×10^{-03}	3	81	*ABHD2, ABR, ADCY5, ADGRV1, ANKRD17, ATP6V0A2, CAMK2D, CCNE1, CD3G, CDC45, CDH10, CHRDL2, CNKSR1, CNRIP1, CRACR2B, DDR1, DPM3, DPYD, EDA, EDIL3, EFNA1, EIF2B1, EPB41L5, FCAR, GALNT2, GCHFR, GDNF, HAS2, HAS3, HERC3, HID1, HPSE, IKBKE, IRAK4, LFNG, LGR4, MAD1L1, MAST4, MED13L, mir-383, NAV2, NCOA3, NCR1, NEDD8, P2RX2, PADI2, PCLO, PDE4D, PHF21A, PIDD1, PIK3CB, PIK3R1, PIP5K1C, PLXDC2, POU2F2, PPFIA4, PSPH, PYGB, RGS7, RMDN3, ROBO1, RPLP2, SEC23A, SERPINF2, SGCZ, SHROOM3, SLC16A5, SLC25A4, ST8SIA1, STEAP3, THRB, THSD4, TIAM1, TMED2, TNFRSF18, TNMD, TNN, TNR, TRHR, UMPS, VAV3*
ELL	7.00×10^{-04}	3	82	*ABCC9, ACTN1, ADGRV1, AREG, ATP13A5, ATP6V0D2, ATXN10, CAMK2D, CARD9, CD3D, CD3E, CD3G, CDC45, CDH10, CRACR2B, DDR1, DOCK10, DPYD, EDA, EDIL3, EFNA1, EIF2B1, FADD, FCAR, FOXM1, GALNT2, GBX2, GCHFR, GDNF, GNAQ, GPSM1, HAS2, HAS3, HERC3, HPSE, IRAK4, ITGB5, LFNG, LGR4, LRP6, MAD1L1, MAST4, MED13L, MZF1, NAV2, NCOA3, NEDD8, NELL2, OSBPL3, PADI2, PCLO, PFKP, PHF21A, PIDD1, PIK3CB, PNPLA2, POU2F2, PPFIA4, PTCH1, PTPRN2, PYGB, RBL2, RGS10, RGS7, RMDN3, ROBO1, RSL1D1, SEC23A, SERPINF2, SHROOM3, SLC25A4, STEAP3, SUCLG2, THBS2, TIAL1, TIAM1, TNFRSF18, TNFRSF4, TNN, TRHR, TSSK4, UMPS*
Ep300/Pcaf	6.60×10^{-03}	3	78	*ABLIM1, ACTN1, ADGRV1, AREG, ATP6V0D2, ATXN10, CAMK2D, CD3E, CD3G, CDC45, CDH10, CHMP4A, CNKSR1, COPA, CRACR2B, DDR1, DLG2, DPYD, EDA, EDIL3, EFNA1, EIF2B1, FADD, FCAR, FOXM1, FZD1, GALNT2, GBX2, GCC1, GNAQ, HAS2, HAS3,*

				HERC3, HPSE, ITGB5, LFNG, LMX1A, LRP6, MAD1L1, MED13L, NCR1, NEDD8, NELL2, NLGN4X, PADI2, PFKP, PHF21A, PIDD1, PIK3CB, PIP5K1C, PNPLA2, POU2F2, PTCH1, PYGB, RBL2, RGS10, RMDN3, ROBO1, RSL1D1, SEC23A, SEC23IP, SEMA3E, SERPINF2, SHROOM3, SLC25A4, STEAP3, THBS2, TIAM1, TJP2, TMED2, TNFRSF18, TNFRSF4, TNN, TSSK4, UMPS, UTRN, VAV3, XRN1
ERCC6	4.00×10^{-03}	3	31	*ACTN1, AREG, ATXN10, CAMK2D, CDH10, CHTF8, CRACR2B, DDR1, EDIL3, EFNA1, EIF2B1, FOXM1, GBX2, HAS2, ITGB5, MAD1L1, MED13L, NEDD8, NELL2, PADI2, PFKP, PIDD1, PTCH1, RBL2, ROBO1, SHROOM3, STEAP3, THBS2, TNFRSF18, TRHR, UMPS*
ERN1	4.20×10^{-03}	3	89	*ABCC9, ABR, ADCY5, ANKRD17, ANO1, ATP13A5, ATP6V0A2, ATXN10, CAMK2D, CCNE1, CD3D, CD3G, CHMP2A, CHMP4A, CHRDL2, CLDN5, CNKSR1, CNRIP1, COPA, CRACR2B, DDR1, DLG2, DPM3, DPYD, EDA, EFNA1, EIF2B1, EPB41L5, FADD, FCAR, GALNT2, GCC1, GCHFR, GDNF, GNAQ, GPSM1, HAS2, HERC3, HID1, IGSF9B, IKBKE, LMF1, MAST4, MED13L, MGA,* mir-383*, MZF1, NCOA3, NEDD8, NELL2, NLGN4X, P2RX2, PADI2, PDE4D, PIDD1, PIK3CB, PLIN2, PLXDC2, POU2F2, PPFIA4, PSPH, PTCH1, RASGEF1B, RBFOX1, RGS7, RMDN3, RPLP2, RSL1D1, SEC23A, SEC23IP, SERPINA1, SGCZ, SHROOM3, SLC16A5, SLC25A4, ST8SIA1, STEAP3, SUCLG2, THRB, THSD4, TIAM1, TJP2, TNMD, TNN, TNR, TRHR, TRPS1, UMPS, XRN1*
FBH1	2.30×10^{-03}	3	88	*ABLIM1, ACTN1, ADGRV1, ANO1, AREG, ARSB, ATP13A5, ATP6V0D2, BEND5, CAMK2D, CCT6A, CD3D, CD3G, CDH10, CHMP4A, COPA, CRACR2B, DDR1, DLG2, DPYD, EDA, EDIL3, EFNA1, EIF2B1, FADD, FCAR, FOXM1, GALNT2, GBX2, GNAQ, HAS2, HAS3, HERC3, HPSE, IGSF9B, IRAK4, ITGB5, LFNG, LGR4, LRP6, MAD1L1, MED13L, NCR1, NEDD8, NELL2, NLGN4X, OSBPL3, PADI2, PCLO, PFKP, PHF21A, PIDD1, PIK3CB, PIK3R1, PLXDC2, PPFIA4, PSPH, PTCH1, PYGB, RASGEF1B, RBFOX1, RBL2, RGS10, RGS7, RMDN3, ROBO1, RSL1D1, SEC23IP, SEMA3E, SERPINF2, SHROOM3, SLC25A4, STEAP3, STXBP6, THBS2, TIAL1, TIAM1, TJP2, TNFRSF18, TNFRSF4, TNN, TRHR, TRPS1, TSSK4, TWF1, UMPS, VAV3, XRN1*

Filamin	9.80 × 10^{-03}	3	80	*ABHD2, ABR, ADCY5, ADGRV1, ANKRD17, ARSB, ATP6V0A2, CCNE1, CD3G, CDC45, CDH10, CHRDL2, CNKSR1, CNRIP1, CRACR2B, DDR1, DPM3, DPYD, EDA, EDIL3, EFNA1, EIF2B1, EPB41L5, FCAR, GALNT2, GCHFR, GDNF, HAS2, HAS3, HERC3, HID1, HPSE, IKBKE, IRAK4, LFNG, MAD1L1, MAST4, MED13L, mir-383, NAV2, NCOA3, NCR1, NEDD8, OSBPL3, P2RX2, PADI2, PCLO, PDE4D, PHF21A, PIDD1, PIK3CB, PIK3R1, PIP5K1C, PLXDC2, POU2F2, PPFIA4, PSPH, PYGB, RMDN3, ROBO1, RPLP2, SEC23A, SERPINF2, SGCZ, SHROOM3, SLC16A5, SLC25A4, ST8SIA1, STEAP3, THRB, THSD4, TIAM1, TMED2, TNFRSF18, TNMD, TNN, TNR, TRHR, UMPS, VAV3*
FLNB	4.70 × 10^{-03}	3	59	*ABCC9, ABLIM1, ABR, ADCY5, ADGRV1, AREG, ARSB, ATP6V0A2, CCNE1, CCT6A, CD3E, CDC45, CHMP4A, CHRDL2, CNRIP1, COPA, DLG2, DPYD, EDA, FCAR, GCHFR, GDNF, GNAQ, HID1, HMG20B, HPSE, IRAK4, LFNG, LGR4, mir-383, MZF1, NAV2, NCOA3, NCR1, NELL2, NLGN4X, OSBPL3, P2RX2, PCLO, PDE4D, PIK3CB, PIK3R1, PLXDC2, PPFIA4, PSPH, PYGB, RGS7, RPLP2, SEC23IP, SGCZ, SLC16A5, SLC25A4, TENM3, THRB, TJP2, TNFRSF4, TNN, TNR, XRN1*
FOSB	5.30 × 10^{-03}	3	63	*ABCC9, ABHD2, ADGRV1, ARSB, ATP6V0D2, CADM2, CAMK2D, CARD9, CCT6A, CD3D, CD3G, CDC45, CDH10, CLDN5, CRACR2B, DDR1, DOCK10, EDIL3, EIF2B1, FADD, FOXP2, FZD1, GBX2, GNAQ, GPSM1, HAS3, HERC3, HMG20B, IRAK4, KRT7, LFNG, LGR4, MAD1L1, MED13L, NCOA3, NCR1, NEDD8, OSBPL3, PADI2, PCLO, PDE5A, PIDD1, PLIN2, PNPLA2, POU2F2, PSPH, PTCH1, PYGB, RGS7, RMDN3, ROBO1, SEC23A, SEMA3E, SHROOM3, ST8SIA1, STEAP3, THSD4, TIAL1, TIAM1, TNFRSF4, UMPS, UNC5C, UTRN*
FZD5	5.90 × 10^{-03}	3	73	*ABHD2, ABR, ADCY5, ADGRV1, ANKRD17, ATP6V0A2, ATXN10, CAMK2D, CCNE1, CD3E, CDH10, CHRDL2, CNRIP1, CRACR2B, DDR1, DPYD, EDA, EDIL3, EIF2B1, FCAR, GDNF, HAS3, HID1, HPSE, IKBKE, IRAK4, KRT7, LFNG, LGR4, MAD1L1, MAST4, MED13L, mir-383, MZF1, NCOA3, NCR1, NEDD8, NELL2, P2RX2, PADI2, PCLO, PDE4D, PHF21A, PIDD1, PIK3CB, PIP5K1C, PLXDC2, PSPH, PTCH1, PTPRN2, PYGB, RBFOX1, ROBO1, RPLP2, SEMA3E, SGCZ, SHROOM3, SLC16A5, SLC25A4, ST8SIA1, STEAP3,*

				SUCLG2, THRB, THSD4, TIAM1, TMED2, TNFRSF18, TNFRSF4, TNN, TNR, TRHR, UMPS, VAV3
GABP	8.50×10^{-03}	2	3	*FCAR, HPSE, UTRN*
GABPB1	3.70×10^{-03}	2	3	*FCAR, RBL2, UTRN*
GCKR	1.80×10^{-03}	3	78	*ABCC9, ABLIM1, ABR, ACTN1, ADCY5, ADGRV1, ANKRD17, ANO1, ARSB, ATP6V0A2, ATXN10, CAMK2D, CCT6A, CD3D, CD3E, CD3G, CDC45, CDH10, CHRDL2, CHRM3, CNRIP1, CRACR2B, DDR1, EDIL3, EIF2B1, EPB41L5, GCHFR, GDNF, HID1, IGSF9B, LFNG, LGR4, MAD1L1, MAST4, MED13L, mir-383, MZF1, NCOA3, NCR1, NELL2, OSBPL3, P2RX2, PADI2, PDE4D, PDE5A, PFKP, PHF21A, PIDD1, PIP5K1C, PPFIA4, PSPH, PTCH1, RASGEF1B, RGS7, ROBO1, RPLP2, RSL1D1, SEC23A, SERPINF2, SGCZ, SHROOM3, SLC16A5, SLC25A4, ST8SIA1, STEAP3, TEAD4, THSD4, TIAL1, TIAM1, TMED2, TNFRSF18, TNFRSF4, TNN, TNR, TRHR, TRPS1, UMPS, VAV3*
GNB5	2.20×10^{-03}	1	2	*RGS6, RGS7*
GNRH2	4.90×10^{-03}	2	31	*ABHD2, ABLIM1, ACTN1, CCNE1, CHMP4A, COPA, DLG2, EFNA1, FOXM1, GNAQ, HAS3, IKBKE, KRT7, NCOA3, NELL2, NLGN4X, PFKP, PHF21A, PTCH1, RBL2, ROBO1, RPLP2, SEC23IP, SERPINA1, THBS2, THRB, TJP2, TNFRSF18, UTRN, VAV3, XRN1*
GSTM5	7.30×10^{-03}	3	63	*ABHD2, ABLIM1, AREG, ARSB, CAMK2D, CCNE1, CDC45, CDH10, CHMP4A, CLDN5, COPA, CRACR2B, DLG2, DOCK10, EDIL3, EIF2B1, FADD, FZD1, GBX2, GDNF, GNAQ, HAS2, HAS3, HPSE, ITGB5, KRT7, LGR4, LRP6, MAD1L1, MED13L, NCOA3, NEDD8, NELL2, NLGN4X, OSBPL3, PADI2, PCLO, PDE5A, PHF21A, PIDD1, PIK3R1, PIP5K1C, PLIN2, POU2F2, PTCH1, RBL2, RGS7, ROBO1, SEC23A, SEC23IP, SHROOM3, SLC25A4, STEAP3, THBS2, THRB, TIAM1, TJP2, TMED2, TNFRSF4, TNN, UMPS, VAV3, XRN1*
H2AFX	6.60×10^{-03}	3	87	*ABLIM1, ACTN1, ADGRV1, ANO1, AREG, ARSB, ATP13A5, ATP6V0D2, ATXN10, BEND5, CAMK2D, CCT6A, CD3D, CD3G, CDH10, CHMP4A, COPA, CRACR2B, DDR1, DLG2, DPYD, EDA, EDIL3, EFNA1, EIF2B1, FADD, FCAR, FOXM1, GALNT2, GBX2, GNAQ, HAS2, HAS3, HERC3, HPSE, IGSF9B, IRAK4, ITGB5, LFNG, LGR4, LRP6, MAD1L1, MED13L, NEDD8, NELL2, NLGN4X, OSBPL3, PADI2, PCLO, PFKP, PHF21A, PIDD1, PIK3CB, PIK3R1, PLXDC2, PPFIA4, PSPH, PTCH1, PYGB, RASGEF1B, RBFOX1, RBL2, RGS10,*

				RGS7, RMDN3, ROBO1, RSL1D1, SEC23IP, SEMA3E, SERPINF2, SHROOM3, SLC25A4, STEAP3, STXBP6, THBS2, TIAL1, TIAM1, TJP2, TNFRSF18, TNFRSF4, TNN, TRHR, TRPS1, TWF1, UMPS, VAV3, XRN1
Hat	2.90×10^{-03}	3	73	*ABHD2, ACTN1, CARD9, CCNE1, CD3D, CD3G, CDC45, CDH10, CHMP2A, CLDN5, CNKSR1, CRACR2B, DDR1, DOCK10, DPM3, EDA, EDIL3, EFNA1, EIF2B1, EPB41L5, FCAR, FZD1, GALNT2, GBX2, GCHFR, GDNF, GNAQ, GPSM1, HAS2, HERC3, HPSE, IKBKE, ITGB5, KRT7, LGR4, LMX1A, LRP6, MAD1L1, MAST4, MED13L, NAV2, NCOA3, NCR1, NEDD8, PADI2, PIDD1, PIK3R1, PLIN2, PLXDC2, PNPLA2, POU2F2, PPFIA4, PTCH1, PYGB, RBL2, RMDN3, ROBO1, SEMA3E, SERPINF1, SERPINF2, SHROOM3, SLC25A4, ST8SIA1, STEAP3, THBS2, THRB, TIAL1, TIAM1, TNFRSF18, TNMD, TRHR, UMPS, UTRN*
Hspa1b	7.80×10^{-03}	3	84	*ABLIM1, ABR, ACTN1, ADCY5, ADGRV1, ANKRD17, ANO1, AREG, ARSB, ATP13A5, ATP6V0A2, ATXN10, CCNE1, CCT6A, CD3D, CD3E, CDC45, CHMP4A, CHRDL2, CNKSR1, CNRIP1, COPA, CRACR2B, DLG2, DPYD, EDA, EIF2B1, FCAR, GDNF, GNAQ, HAS3, HERC3, HID1, HPSE, IGSF9B, IRAK4, LFNG, LGR4, MAD1L1, MAST4, MED13L, mir-383, MZF1, NAV2, NCOA3, NEDD8, NLGN4X, P2RX2, PADI2, PCLO, PDE4D, PDE5A, PHF21A, PIDD1, PIK3CB, PIK3R1, PLXDC2, PSPH, PTPRN2, PYGB, RASGEF1B, RBFOX1, RGS7, RMDN3, SEC23IP, SGCZ, SHROOM3, SLC16A5, SLC25A4, ST8SIA1, STEAP3, SUCLG2, THRB, THSD4, TIAL1, TIAM1, TJP2, TNFRSF4, TNN, TNR, TRHR, TRPS1, UMPS, XRN1*
HUWE1	7.70×10^{-03}	3	84	*ABLIM1, ACTN1, ADGRV1, ANKRD17, AREG, ARSB, ATP13A5, ATP6V0D2, CAMK2D, CCNE1, CCT6A, CD3D, CD3E, CD3G, CDH10, CHMP4A, COPA, CRACR2B, DDR1, DLG2, DOCK10, DPYD, EDIL3, EFNA1, EIF2B1, FCAR, FOXM1, FZD1, GALNT2, GCHFR, GDNF, GNAQ, HAS2, HMG20B, IGSF9B, IRAK4, KRT7, LFNG, LRP6, MAD1L1, MED13L, MZF1, NCR1, NEDD8, NELL2, NLGN4X, PADI2, PCLO, PDE5A, PFKP, PHF21A, PIDD1, PIK3CB, PIK3R1, PLXDC2, PNPLA2, PPFIA4, PSPH, PYGB, RASGEF1B, RBFOX1, RBL2, RGS10, RGS7, ROBO1, RSL1D1, SEC23A, SEC23IP, SEMA3E, SERPINA1, SERPINF2, SHROOM3, SLC16A5, STEAP3, SUCLG2,*

				TEAD4, TIAM1, TJP2, TNFRSF18, TRPS1, TSSK4, UMPS, VAV3, XRN1
IC261	2.50 × 10^{-03}	3	75	*ABCC9, ACTN1, AREG, ATP6V0D2, CADM2, CAMK2D, CCT6A, CD3D, CD3E, CD3G, CDH10, CRACR2B, DDR1, DPYD, EDA, EDIL3, EFNA1, EIF2B1, FCAR, FOXM1, FOXP2, GALNT2, GBX2, GCHFR, HAS2, HERC3, HMG20B, HPSE, IGSF9B, IRAK4, ITGB5, LGR4, MAD1L1, MED13L, MZF1, NCOA3, NCR1, NEDD8, PADI2, PCLO, PDE4D, PDE5A, PFKP, PHF21A, PIDD1, PIK3CB, PLXDC2, PNPLA2, PPFIA4, PSPH, PTCH1, RASGEF1B, RBL2, RGS10, RMDN3, ROBO1, RSL1D1, SEC23A, SEMA3E, SERPINF2, SHROOM3, SLC25A4, ST8SIA1, STEAP3, SUCLG2, THBS2, THSD4, TIAM1, TNFRSF18, TNN, TRHR, TRPS1, TSSK4, UMPS, UNC5C*
IC261	9.90 × 10^{-03}	2	28	*ACTN1, AREG, CAMK2D, CDH10, CRACR2B, DDR1, EDIL3, EFNA1, EIF2B1, FOXM1, GBX2, HAS2, ITGB5, MAD1L1, MED13L, NEDD8, PADI2, PCLO, PFKP, PIDD1, PTCH1, RBL2, ROBO1, SHROOM3, STEAP3, THBS2, TNFRSF18, UMPS*
IL13RA2	7.90 × 10^{-03}	3	87	*ABCC9, ABLIM1, ABR, ADCY5, ANKRD17, ANO1, AREG, ARSB, ATP13A5, CCNE1, CCT6A, CD3D, CD3E, CD3G, CDC45, CHMP4A, CNRIP1, COPA, CRACR2B, DDHD1, DLG2, DOCK10, EDA, EIF2B1, FCAR, FOXM1, FZD1, GCHFR, GNAQ, GRM8, HAS2, HERC3, HID1, HMG20B, HPSE, IGSF9B, IKBKE, IPO4, IRAK4, LGR4, MAST4, MED13L, mir-383, NAV2, NCOA3, NCR1, NEDD8, NELL2, NLGN4X, P2RX2, PADI2, PCLO, PDE5A, PFKP, PIDD1, PIK3CB, PIP5K1C, PITRM1, PLXDC2, PNPLA2, POU2F2, PPFIA4, RASGEF1B, RBFOX1, RBL2, RGS7, RMDN3, SEC23A, SEC23IP, SERPINA1, SERPINF1, SERPINF2, SHROOM3, SLC16A5, STEAP3, TANC2, THRB, TJP2, TMED2, TMTC2, TNFRSF4, TNMD, TNR, TRHR, TRPS1, UMPS, XRN1*
IL1RAP	8.80 × 10^{-03}	3	80	*ABHD2, ABR, ADCY5, ADGRV1, ANKRD17, ARSB, ATP6V0A2, CAMK2D, CCNE1, CD3G, CDH10, CHRDL2, CNKSR1, CNRIP1, CRACR2B, DDR1, DPM3, DPYD, EDA, EDIL3, EFNA1, EIF2B1, EPB41L5, FCAR, GALNT2, GCHFR, GDNF, HAS2, HAS3, HERC3, HID1, IKBKE, IRAK4, LFNG, LGR4, MAST4, MED13L, mir-383, MZF1, NAV2, NCOA3, NCR1, NEDD8, OSBPL3, P2RX2, PADI2, PCLO, PDE4D, PHF21A, PIDD1, PIP5K1C, PLXDC2, POU2F2, PPFIA4, PSPH, PYGB, RGS7, RMDN3, ROBO1, RPLP2, SEC23A, SERPINF2,*

				SGCZ, SHROOM3, SLC16A5, SLC25A4, ST8SIA1, STEAP3, SUCLG2, THRB, THSD4, TIAM1, TMED2, TNFRSF18, TNMD, TNN, TNR, TRHR, UMPS, VAV3
imidazoquinolines	6.10×10^{-03}	3	82	*ABHD2, ABR, ADCY5, ADGRV1, ANKRD17, ATP6V0A2, CAMK2D, CCNE1, CD3G, CDC45, CDH10, CHRDL2, CNKSR1, CNRIP1, CRACR2B, DDR1, DENND4C, DPM3, DPYD, EDA, EDIL3, EFNA1, EIF2B1, EPB41L5, FCAR, GALNT2, GCHFR, GDNF, HAS2, HAS3, HERC3, HID1, HPSE, IKBKE, IRAK4, LFNG, LGR4, MAD1L1, MAST4, MED13L, mir-383, NAV2, NCOA3, NCR1, NEDD8, P2RX2, PADI2, PCLO, PDE4D, PHF21A, PIDD1, PIK3CB, PIK3R1, PIP5K1C, PLXDC2, POU2F2, PPFIA4, PSPH, PYGB, RGS7, RMDN3, ROBO1, RPLP2, SEC23A, SERPINF2, SGCZ, SHROOM3, SLC16A5, SLC25A4, ST8SIA1, STEAP3, THRB, THSD4, TIAM1, TMED2, TNFRSF18, TNMD, TNN, TNR, TRHR, UMPS, VAV3*
ING1	8.90×10^{-03}	3	74	*ACTN1, ADGRV1, AREG, ATP6V0D2, CAMK2D, CD3E, CD3G, CDC45, CDH10, CRACR2B, DDR1, DPYD, EDA, EDIL3, EFNA1, EIF2B1, FADD, FCAR, FOXM1, FZD1, GALNT2, GBX2, GCHFR, GDNF, HAS2, HAS3, HERC3, HPSE, IRAK4, ITGB5, LFNG, LGR4, LRP6, MAD1L1, MAST4, MED13L, MZF1, NAV2, NCOA3, NCR1, NEDD8, PADI2, PCLO, PFKP, PHF21A, PIDD1, PIK3CB, PNPLA2, POU2F2, PPFIA4, PTCH1, PTPRN2, PYGB, RBFOX1, RBL2, RGS10, RMDN3, ROBO1, RSL1D1, SEC23A, SEMA3E, SERPINF2, SHROOM3, SLC25A4, STEAP3, SUCLG2, THBS2, TIAM1, TNFRSF18, TNN, TRHR, TSSK4, UMPS, UTRN*
Jmy-p300	6.70×10^{-03}	3	78	*ABLIM1, ACTN1, ADGRV1, AREG, ATP6V0D2, ATXN10, CAMK2D, CD3E, CD3G, CDC45, CDH10, CHMP4A, CNKSR1, COPA, CRACR2B, DDR1, DLG2, DPYD, EDA, EDIL3, EFNA1, EIF2B1, FADD, FCAR, FOXM1, FZD1, GALNT2, GBX2, GCC1, GNAQ, HAS2, HAS3, HERC3, HPSE, ITGB5, LFNG, LRP6, MAD1L1, MED13L, NCR1, NEDD8, NELL2, NLGN4X, PADI2, PFKP, PHF21A, PIDD1, PIK3CB, PIP5K1C, PNPLA2, POU2F2, PTCH1, PYGB, RBL2, RGS10, RMDN3, ROBO1, RSL1D1, SEC23A, SEC23IP, SEMA3E, SERPINF2, SHROOM3, SLC25A4, STEAP3, THBS2, TIAM1, TJP2, TMED2, TNFRSF18, TNFRSF4, TNN, TRHR, TSSK4, UMPS, UTRN, VAV3, XRN1*

KAT5	2.60×10^{-03}	3	93	*ABCC9, ACTN1, ADGRV1, ANKRD17, AREG, ARSB, ATP13A5, ATP6V0A2, BEND5, CAMK2D, CCNE1, CD3D, CD3G, CDC45, CDH10, CHMP4A, COPA, CRACR2B, DDR1, DLG2, DOCK10, DPM3, DPYD, EDIL3, EFNA1, EIF2B1, EPB41L5, FOXM1, GALNT2, GNAQ, HAS2, HMG20B, IGSF9B, IKBKE, IRAK4, KRT7, LFNG, LRP6, MAD1L1, MAST4, MED13L, MZF1, NAV2, NCR1, NEDD8, NELL2, NLGN4X, OSBPL3, PADI2, PCLO, PDE4D, PFKP, PHF21A, PIDD1, PIK3CB, PIP5K1C, PLIN2, PLXDC2, PTCH1, PYGB, RASGEF1B, RBFOX1, RBL2, RGS10, RGS7, ROBO1, RPLP2, SEC23A, SEC23IP, SEMA3E, SERPINA1, SHROOM3, SLC16A5, ST8SIA1, STEAP3, STXBP6, SUCLG2, TEAD4, THSD4, TIAL1, TIAM1, TJP2, TMED2, TNFRSF18, TNFRSF4, TRHR, TRPM3, TRPS1, TSSK4, TWF1, UMPS, VAV3, XRN1*
KCNA3	2.20×10^{-03}	3	59	*ABHD2, ACTN1, ADGRV1, AREG, ATP13A5, ATP6V0D2, CAMK2D, CCNE1, CD3D, CDC45, CDH10, CLDN5, CRACR2B, DDR1, EDA, EDIL3, EFNA1, EIF2B1, FZD1, GBX2, GDNF, HAS2, HPSE, IGSF9B, IKBKE, IRAK4, ITGB5, LFNG, LGR4, LRP6, MAD1L1, MED13L, NEDD8, OSBPL3, PADI2, PCLO, PFKP, PIDD1, PIP5K1C, PLXDC2, PSPH, RASGEF1B, RBFOX1, RBL2, ROBO1, SEC23A, SEMA3E, SERPINF1, SHROOM3, STEAP3, THBS2, THRB, TMED2, TNFRSF18, TNFRSF4, TNN, TRPS1, UMPS, UTRN*
KLC1	8.80×10^{-03}	3	70	*ABHD2, ABR, ADCY5, ANKRD17, ATP13A5, ATP6V0A2, CAMK2D, CCNE1, CD3E, CDH10, CHRDL2, CNRIP1, CRACR2B, DDR1, DPYD, EDA, EDIL3, EIF2B1, FCAR, FZD1, GDNF, HAS3, HID1, HPSE, IKBKE, IRAK4, KRT7, LGR4, MAD1L1, MED13L, mir-383, NAV2, NCOA3, NCR1, NEDD8, OSBPL3, P2RX2, PADI2, PCLO, PDE4D, PHF21A, PIDD1, PIK3CB, PIP5K1C, PLXDC2, PSPH, PYGB, RBFOX1, RGS7, ROBO1, RPLP2, SEC23A, SEMA3E, SGCZ, SHROOM3, SLC16A5, SLC25A4, ST8SIA1, STEAP3, THRB, THSD4, TIAM1, TMED2, TNFRSF18, TNFRSF4, TNN, TNR, TRHR, UMPS, VAV3*
KLRD1	4.10×10^{-03}	1	2	*CD3E, NCR1*
KRT18	1.00×10^{-03}	3	79	*ABLIM1, ABR, ADCY5, ARSB, ATP13A5, ATP6V0A2, CAMK2D, CCNE1, CCT6A, CD3E, CDC45, CHMP2A, CHMP4A, CNKSR1, CNRIP1, COPA, CRACR2B, DDR1, DLG2, DPYD, EDIL3, EIF2B1, FCAR, FOXM1, GCHFR, GDNF, GNAQ, HERC3, HID1, HMG20B,*

				IRAK4, KRT7, MAD1L1, MED13L, mir-383, MZF1, NAV2, NCR1, NEDD8, NLGN4X, OSBPL3, P2RX2, PADI2, PDE4D, PDE5A, PHF21A, PIDD1, PIK3R1, PIP5K1C, PKDCC, PLXDC2, PPFIA4, PSPH, RBFOX1, RGS7, RPLP2, SEC23A, SEC23IP, SEMA3E, SERPINF2, SGCZ, SHROOM3, SLC16A5, STEAP3, SUCLG2, THBS2, THSD4, TIAL1, TIAM1, TJP2, TMED2, TNMD, TNN, TNR, TRHR, UMPS, UTRN, VAV3, XRN1
KRT8	3.20×10^{-03}	3	75	*ABHD2, ABR, ADCY5, ADGRV1, ANKRD17, ATP6V0A2, ATXN10, CCNE1, CD3E, CDH10, CHRDL2, CNRIP1, CRACR2B, DDR1, DPYD, EDA, EDIL3, EIF2B1, FCAR, GDNF, HAS3, HID1, HPSE, IKBKE, IRAK4, KRT7, LFNG, LGR4, MAD1L1, MAST4, MED13L, mir-383, MZF1, NAV2, NCOA3, NEDD8, NELL2, P2RX2, PADI2, PCLO, PDE4D, PHF21A, PIDD1, PIK3CB, PIP5K1C, PLXDC2, PSPH, PTPRN2, PYGB, RBFOX1, RGS7, ROBO1, RPLP2, SEC23A, SEMA3E, SERPINF2, SGCZ, SHROOM3, SLC16A5, SLC25A4, ST8SIA1, STEAP3, SUCLG2, THRB, THSD4, TIAM1, TMED2, TNFRSF18, TNFRSF4, TNMD, TNN, TNR, TRHR, UMPS, VAV3*
KSR2	2.40×10^{-03}	3	93	*ABLIM1, ABR, ACTN1, ADCY5, ADGRV1, ANKRD17, ANO1, AREG, ARSB, ATP13A5, ATP6V0A2, CAMK2D, CCNE1, CCT6A, CD3D, CD3G, CDC45, CHMP4A, CHRDL2, CNKSR1, CNRIP1, COPA, CRACR2B, DDR1, DLG2, DPM3, DPYD, EDA, EFNA1, EIF2B1, EPB41L5, FCAR, GALNT2, GCHFR, GDNF, GNAQ, HAS2, HAS3, HERC3, HID1, HPSE, IGSF9B, IKBKE, IRAK4, LFNG, LGR4, MAD1L1, MAST4, MED13L, mir-383, NAV2, NCOA3, NCR1, NEDD8, NELL2, NLGN4X, P2RX2, PADI2, PCLO, PDE4D, PDE5A, PHF21A, PIDD1, PIK3CB, PLXDC2, POU2F2, PPFIA4, PSPH, PYGB, RASGEF1B, RBFOX1, RGS7, RMDN3, SEC23IP, SERPINF2, SGCZ, SHROOM3, SLC16A5, SLC25A4, ST8SIA1, STEAP3, THRB, THSD4, TIAL1, TIAM1, TJP2, TNMD, TNN, TNR, TRHR, TRPS1, UMPS, XRN1*
LCN2	5.00×10^{-03}	2	38	*ACTL6A, ACTN1, CAMK2D, CCNE1, CDH10, CLDN5, CNRIP1, CRACR2B, DDR1, DOCK10, EDIL3, EFNA1, EIF2B1, FOXM1, FZD1, GBX2, IKBKE, ITGB5, KRT7, MAD1L1, MED13L, NCOA3, NEDD8, PADI2, PFKP, PIDD1, PNPLA2, POU2F2, PTCH1, RBL2, ROBO1, SERPINA1, SERPINF1, SHROOM3, STEAP3, TNFRSF18, UMPS, UTRN*

LGALS7/LGALS7B	1.80 × 10^{-03}	3	73	*ABHD2, ABLIM1, ABR, ADCY5, ADGRV1, ARSB, ATP6V0A2, ATXN10, CCNE1, CD3D, CD3E, CDH10, CHMP4A, CHRDL2, CNRIP1, COPA, CRACR2B, DDR1, DLG2, EDA, EDIL3, EIF2B1, FCAR, GDNF, GNAQ, HID1, HPSE, IGSF9B, IRAK4, LFNG, MAD1L1, MAST4, MED13L, mir-383, MZF1, NAV2, NELL2, NLGN4X, OSBPL3, P2RX2, PADI2, PDE4D, PIDD1, PIK3CB, PIK3R1, PIP5K1C, PSPH, PTPRN2, RASGEF1B, RBFOX1, RGS10, RPLP2, SEC23A, SEC23IP, SEMA3E, SGCZ, SHROOM3, SLC16A5, SLC25A4, ST8SIA1, STEAP3, SUCLG2, TENM3, THSD4, TJP2, TMED2, TNFRSF18, TNN, TNR, TRPS1, UMPS, VAV3, XRN1*
Lh	3.80 × 10^{-03}	1	6	*ACTN1, AREG, HAS2, ITGB5, PDE4D, THBS2*
LPAR5	5.90 × 10^{-03}	3	45	*ACTN1, ADGRV1, AREG, ATP13A5, CAMK2D, CCNE1, CD3D, CDH10, CLDN5, CRACR2B, DDR1, EDIL3, EFNA1, EIF2B1, FOXM1, GBX2, GNAQ, IGSF9B, IRAK4, ITGB5, LFNG, MAD1L1, MED13L, NEDD8, OSBPL3, PADI2, PCLO, PFKP, PIDD1, PLIN2, PLXDC2, POU2F2, PSPH, RASGEF1B, RBFOX1, RBL2, ROBO1, SERPINA1, SERPINF1, SHROOM3, STEAP3, THBS2, TNFRSF18, TRPS1, UMPS*
L-serine	6.30 × 10^{-03}	1	2	*CCNE1, PSPH*
mannan	6.80 × 10^{-03}	3	84	*ABLIM1, ABR, ACTN1, ADCY5, ADGRV1, ANKRD17, AREG, ATP13A5, ATP6V0A2, CAMK2D, CCNE1, CCT6A, CD3D, CD3G, CHMP4A, CHRDL2, CNKSR1, CNRIP1, COPA, CRACR2B, DDHD1, DDR1, DLG2, DPM3, DPYD, EFNA1, EIF2B1, EPB41L5, FCAR, GALNT2, GCC1, GCHFR, GNAQ, HAS2, HAS3, HERC3, HID1, HMCN1, IGSF9B, IKBKE, IRAK4, LFNG, MAD1L1, MED13L, mir-383, NCOA3, NCR1, NEDD8, NELL2, NLGN4X, P2RX2, PADI2, PCLO, PDE4D, PDE5A, PHF21A, PIDD1, PIK3CB, PLXDC2, POU2F2, PPFIA4, PSPH, RASGEF1B, RBFOX1, RGS7, RMDN3, SEC23IP, SERPINF2, SGCZ, SHROOM3, SLC16A5, ST8SIA1, STEAP3, SUCLG2, THSD4, TIAL1, TIAM1, TJP2, TNMD, TNN, TNR, TRPS1, UMPS, XRN1*
MAP2K3	6.60 × 10^{-03}	2	47	*ACTN1, AREG, ATP6V0D2, CAMK2D, CCNE1, CDH10, CLDN5, CRACR2B, DDR1, DPYD, EDIL3, EFNA1, EIF2B1, FADD, FOXM1, GBX2, GDNF, HAS2, HPSE, IKBKE, ITGB5, LRP6, MAD1L1, MED13L, MGA, NEDD8, PADI2, PCLO, PIDD1, PIP5K1C, PLIN2, PNPLA2, POU2F2, PTCH1, RBL2, RGS7, ROBO1, SEC23A, SERPINA1,*

				SHROOM3, STEAP3, TIAM1, TMED2, TNFRSF18, TNN, TRHR, UMPS
MAP3K3	3.80×10^{-03}	3	94	*ABCC9, ABR, ACTN1, ADCY5, ADGRV1, ANO1, AREG, ARSB, ATP6V0A2, ATXN10, CAMK2D, CARD9, CD3E, CD3G, CDC45, CHMP2A, CHMP4A, CHRDL2, CLDN5, CNKSR1, CNRIP1, COPA, CRACR2B, DDR1, DLG2, DPYD, EDA, EFNA1, EIF2B1, EPB41L5, FADD, FOXM1, FSCN2, FZD1, GALNT2, GCC1, GCHFR, GDNF, GNAQ, GPSM1, HAS2, HAS3, HERC3, HID1, HPSE, IKBKE, ITGB5, LFNG, LMF1, LRP6, MED13L, MGA, NCOA3, NCR1, NEDD8, NELL2, NLGN4X, OSBPL3, P2RX2, PADI2, PDE4D, PDE5A, PIDD1, PIK3R1, PLIN2, POU2F2, PPFIA4, PSPH, PTCH1, PTPRN2, PYGB, RGS7, RMDN3, RSL1D1, SEC23IP, SERPINA1, SGCZ, SHROOM3, SLC16A5, SLC22A23, SLC25A4, SOX8, STEAP3, TANC2, THBS2, THRB, TMTC2, TNFRSF4, TNN, TNR, TRHR, TWF1, UMPS, XRN1*
MAP3K5	4.80×10^{-03}	2	44	*ACTN1, AREG, ARSB, CAMK2D, CCNE1, CDH10, CLDN5, CRACR2B, DDR1, DOCK10, EDIL3, EFNA1, EIF2B1, FADD, FOXM1, GBX2, GDNF, HAS2, ITGB5, LRP6, MAD1L1, MED13L, NEDD8, OSBPL3, PADI2, PDE5A, PFKP, PIDD1, PIP5K1C, PLIN2, POU2F2, PTCH1, RBL2, RGS7, ROBO1, SEC23A, SHROOM3, STEAP3, THBS2, TMED2, TNFRSF18, TNN, TRHR, UMPS*
mir-132	4.40×10^{-03}	1	3	*FOXP2, IRAK4, PTCH1*
miR-29b-3p	4.10×10^{-03}	3	70	*ABHD2, ACTN1, ADGRV1, ARSB, ATP13A5, ATP6V0D2, CAMK2D, CD3D, CD3G, CRACR2B, DDR1, DOCK10, DPM3, DPYD, EDA, EDIL3, EFNA1, EIF2B1, EPB41L5, FADD, FCAR, GALNT2, GCHFR, GDNF, GNAQ, HAS2, HERC3, IGSF9B, IKBKE, LFNG, LMF1, LRP6, MAD1L1, MAST4, MED13L, MZF1, NAV2, NCOA3, NCR1, NELL2, OSBPL3, PADI2, PDE5A, PIDD1, PIK3CB, PIK3R1, PIP5K1C, PNPLA2, POU2F2, PPFIA4, RASGEF1B, RBFOX1, RGS7, RMDN3, ROBO1, SEC23A, SEMA3E, SERPINF1, SERPINF2, SHROOM3, SLC25A4, STEAP3, SUCLG2, TIAM1, TJP2, TNFRSF18, TNMD, TNN, TRPS1, UMPS*
mir-630	7.70×10^{-03}	3	79	*ACTN1, ADGRV1, ANO1, AREG, ARSB, ATP13A5, ATP6V0D2, BEND5, CAMK2D, CCT6A, CD3D, CD3G, CDH10, CRACR2B, DDR1, DPYD, EDA, EDIL3, EFNA1, EIF2B1, FADD, FCAR, FOXM1, GALNT2, GBX2, HAS2, HAS3, HERC3, HPSE, IGSF9B, IRAK4, ITGB5, LFNG, LGR4, LRP6, MAD1L1, MED13L, NCOA3, NCR1, NEDD8, OSBPL3,*

				PADI2, PCLO, PFKP, PHF21A, PIDD1, PIK3CB, PIK3R1, PLXDC2, PPFIA4, PSPH, PTCH1, PYGB, RASGEF1B, RBFOX1, RBL2, RGS10, RGS7, RMDN3, ROBO1, RSL1D1, SEMA3E, SERPINF2, SHROOM3, SLC25A4, STEAP3, STXBP6, THBS2, TIAL1, TIAM1, TNFRSF18, TNFRSF4, TNN, TRHR, TRPS1, TSSK4, TWF1, UMPS, VAV3
mir-8	6.30×10^{-03}	3	86	*ABLIM1, ACTN1, ANKRD17, AREG, ARSB, ATP13A5, ATP6V0D2, CAMK2D, CCT6A, CD3E, CD3G, CDH10, CHMP2A, CHMP4A, CHRDL2, CNKSR1, COPA, CRACR2B, DDR1, DLG2, DOCK10, DPYD, EDA, EDIL3, EFNA1, EIF2B1, FCAR, FOXM1, GALNT2, GBX2, GEMIN2, GNAQ, HAS2, HAS3, HERC3, HID1, HMG20B, HPSE, IRAK4, ITGB5, LMX1A, LRP6, MAD1L1, MED13L, MGA, MZF1, NCR1, NEDD8, NLGN4X, OSBPL3, PADI2, PCLO, PDE5A, PFKP, PHF21A, PIDD1, PIK3R1, PIP5K1C, PKDCC, PTCH1, RBFOX1, RBL2, RGS10, RGS7, ROBO1, RSL1D1, SEC23A, SEC23IP, SERPINF2, SHROOM3, ST8SIA1, STEAP3, SUCLG2, TIAL1, TIAM1, TJP2, TMED2, TNFRSF18, TNFRSF4, TNN, TRHR, TSSK4, UMPS, UTRN, VAV3, XRN1*
MKK1/4	6.00×10^{-03}	3	71	*ABLIM1, ABR, ADCY5, ADGRV1, ARSB, ATP13A5, ATP6V0A2, CAMK2D, CCNE1, CD3E, CHMP4A, CHRDL2, CNRIP1, COPA, CRACR2B, DDR1, DLG2, DPYD, EDA, EDIL3, EIF2B1, FCAR, GDNF, GNAQ, HID1, HMG20B, HPSE, IRAK4, LFNG, MAD1L1, MED13L, mir-383, NAV2, NCOA3, NCR1, NEDD8, NELL2, NLGN4X, P2RX2, PADI2, PCLO, PDE4D, PIDD1, PIK3CB, PIK3R1, PLXDC2, PSPH, PTCH1, PYGB, RBFOX1, RGS7, ROBO1, RPLP2, SEC23IP, SEMA3E, SGCZ, SHROOM3, SLC16A5, SLC25A4, ST8SIA1, STEAP3, THRB, THSD4, TIAL1, TJP2, TNFRSF4, TNN, TNR, TRHR, UMPS, XRN1*
MLH1	8.40×10^{-03}	2	29	*ACTN1, AREG, CAMK2D, CCNE1, CDH10, CRACR2B, DDR1, EDIL3, EFNA1, EIF2B1, FOXM1, GBX2, HAS2, ITGB5, LRP6, MAD1L1, MED13L, NEDD8, PADI2, PFKP, PIDD1, PTCH1, ROBO1, SHROOM3, STEAP3, THBS2, TNFRSF18, TNN, UMPS*
NBR1	5.50×10^{-03}	2	31	*ACTN1, AREG, CAMK2D, CD3D, CD3E, CDH10, CRACR2B, DDR1, EDIL3, EFNA1, EIF2B1, FOXM1, GBX2, HAS2, ITGB5, MAD1L1, MED13L, NEDD8, PADI2, PFKP, PIDD1, PTCH1, RBL2, ROBO1, SHROOM3, STEAP3, THBS2, TJP2, TNFRSF18, UMPS, UTRN*
NCOA6	7.00×10^{-03}	2	25	*ABHD2, ABLIM1, AREG, CHMP4A, CLDN5, COPA, DLG2, EFNA1, FOXM1, GNAQ, IKBKE, KRT7, NCOA3, NELL2, NLGN4X, PFKP,*

				PTCH1, RBL2, ROBO1, SEC23IP, THRB, TJP2, TNFRSF18, VAV3, XRN1
Ncoa6	7.40×10^{-03}	3	64	*ACTN1, ADGRV1, AREG, ATP13A5, ATP6V0D2, CAMK2D, CARD9, CD3D, CDC45, CLDN5, CRACR2B, DDR1, DOCK10, EDIL3, EIF2B1, FADD, FCAR, FOXM1, FZD1, GBX2, GNAQ, GPSM1, HAS3, HPSE, IKBKE, IRAK4, LFNG, LGR4, LRP6, MAD1L1, MED13L, NCR1, NEDD8, OSBPL3, PADI2, PCLO, PFKP, PHF21A, PIDD1, PIK3R1, PIP5K1C, PLIN2, PNPLA2, POU2F2, RBFOX1, RBL2, RGS10, ROBO1, SEC23A, SEMA3E, SERPINA1, SERPINF1, SHROOM3, SLC25A4, STEAP3, THRB, THSD4, TIAL1, TIAM1, TJP2, TMED2, TMTC2, UMPS, UTRN*
NCOA7	6.90×10^{-03}	2	24	*ABHD2, ABLIM1, AREG, CHMP4A, COPA, DLG2, EFNA1, FOXM1, GNAQ, IKBKE, KRT7, NCOA3, NELL2, NLGN4X, PTCH1, RBL2, ROBO1, SEC23IP, SERPINF1, THRB, TJP2, TNFRSF18, VAV3, XRN1*
NFkB1-CRel	6.80×10^{-03}	3	48	*ACTN1, AREG, ARSB, ATP13A5, ATP6V0D2, CAMK2D, CARD9, CD3D, CD3E, CD3G, CDC45, CLDN5, CRACR2B, DDR1, EDIL3, EFNA1, EIF2B1, FADD, GBX2, GNAQ, GPSM1, HAS2, HPSE, IKBKE, ITGB5, MAD1L1, MED13L, NCR1, NEDD8, PADI2, PFKP, PIDD1, PIK3R1, POU2F2, PTCH1, RBFOX1, ROBO1, SEMA3E, SERPINA1, SHROOM3, SLC25A4, STEAP3, THBS2, TIAL1, TJP2, TNFRSF18, TNFRSF4, UMPS*
nitrolinoleate	3.30×10^{-03}	3	64	*ACTN1, ADGRV1, ATP13A5, ATP6V0D2, CAMK2D, CARD9, CD3D, CDC45, CLDN5, CRACR2B, DDR1, DOCK10, EDIL3, EIF2B1, FADD, FCAR, FOXM1, FZD1, GBX2, GNAQ, GPSM1, HAS3, HPSE, IKBKE, IRAK4, LFNG, LGR4, LRP6, MAD1L1, MED13L, NCR1, NEDD8, OSBPL3, PADI2, PCLO, PFKP, PHF21A, PIDD1, PIK3R1, PIP5K1C, PLIN2, PNPLA2, POU2F2, RBFOX1, RBL2, RGS10, ROBO1, SEC23A, SEMA3E, SERPINA1, SERPINF1, SHROOM3, SLC25A4, STEAP3, THRB, THSD4, TIAL1, TIAM1, TJP2, TMED2, TMTC2, TNN, UMPS, UTRN*
NPY	3.70×10^{-03}	3	92	*ABCC9, ABR, ACTN1, ADCY5, ADGRV1, ANKRD17, ANO1, AREG, ARSB, ATP6V0A2, ATXN10, CADM2, CARD9, CCNE1, CD3D, CD3G, CHMP2A, CHMP4A, CHRDL2, CNKSR1, CNRIP1, COPA, CRACR2B, DDR1, DLG2, DPM3, DPYD, EDA, EFNA1, EIF2B1, FZD1, GCC1, GCHFR, GNAQ, GPAT3, GPSM1, GRM8, HAS2, HAS3, HERC3, HID1, HMG20B, HPSE, IGSF9B, IRAK4, LFNG, LMF1, LRP6,*

				MED13L, mir-383, MZF1, NCOA3, NCR1, NEDD8, NELL2, NLGN4X, OSBPL3, P2RX2, PADI2, PCLO, PDE5A, PIDD1, PLXDC2, POU2F2, PPFIA4, PSPH, PTCH1, PYGB, RASGEF1B, RGS7, RMDN3, RPLP2, RSL1D1, SEC23IP, SERPINF2, SHROOM3, SLC16A5, SOX8, ST8SIA1, STEAP3, TANC2, THRB, THSD4, TIAL1, TMTC2, TNFRSF4, TNN, TNR, TRHR, TRPS1, UMPS, XRN1
Nr1h	6.70×10^{-03}	3	70	*ABHD2, ACTN1, ADGRV1, CAMK2D, CARD9, CCNE1, CD3D, CD3G, CDC45, CDH10, CHMP2A, CNKSR1, CRACR2B, DDR1, DOCK10, DPM3, EDA, EFNA1, EIF2B1, EPB41L5, FADD, FCAR, GALNT2, GCHFR, GDNF, GNAQ, GPSM1, HAS2, HERC3, HPSE, IKBKE, IRAK4, LFNG, LRP6, MAD1L1, MAST4, MED13L, mir-383, NAV2, NCOA3, NCR1, NEDD8, PADI2, PDE5A, PIDD1, PIK3R1, PLXDC2, PNPLA2, POU2F2, PPFIA4, PTCH1, RGS10, RMDN3, ROBO1, SERPINA1, SERPINF2, SGCZ, SHROOM3, SLC25A4, STEAP3, THRB, TIAL1, TIAM1, TMTC2, TNFRSF18, TNMD, TNN, TRHR, UMPS, UTRN*
NR2F2	1.80×10^{-03}	2	36	*ACTN1, ANKRD17, AREG, CAMK2D, CD3G, CDH10, CRACR2B, DDR1, EDIL3, EFNA1, EIF2B1, FADD, FCAR, FOXM1, FZD1, ITGB5, KRT7, MAD1L1, MED13L, NEDD8, PADI2, PFKP, PIDD1, PLIN2, PTCH1, RBL2, ROBO1, SEMA3E, SERPINA1, SERPINF1, SHROOM3, STEAP3, THBS2, THRB, TNFRSF18, UMPS*
NUMB/NUMBL	2.60×10^{-03}	3	37	*ACTN1, AREG, CAMK2D, CCNE1, CDC45, CDH10, CRACR2B, DDR1, EDIL3, EFNA1, EIF2B1, GBX2, HAS2, HAS3, HPSE, IKBKE, ITGB5, LFNG, LRP6, MAD1L1, MED13L, NEDD8, PADI2, PFKP, PIDD1, PIK3R1, POU2F2, RBL2, ROBO1, SERPINF1, SHROOM3, SLC25A4, STEAP3, TNFRSF18, TNN, TRHR, UMPS*
PASK	3.70×10^{-03}	3	76	*ABCC9, ABLIM1, ANKRD17, ANO1, AREG, ARSB, ATP13A5, ATP6V0D2, CAMK2D, CARD9, CD3D, CD3E, CD3G, CDH10, CHMP2A, CHMP4A, COPA, CRACR2B, DDR1, DLG2, DOCK10, EDIL3, EIF2B1, GCHFR, HAS2, HAS3, HERC3, IGSF9B, LGR4, MAD1L1, MAST4, MED13L, MZF1, NAV2, NCR1, NEDD8, NELL2, NLGN4X, OSBPL3, P2RX2, PADI2, PCLO, PDE4D, PDE5A, PIDD1, PIK3CB, PIK3R1, PIP5K1C, PLXDC2, PPFIA4, PSPH, PYGB, RASGEF1B, RBFOX1, RMDN3, ROBO1, RSL1D1, SEC23A, SEC23IP, SEMA3E, SERPINF2, SHROOM3, SLC25A4, ST8SIA1, STEAP3,*

				SUCLG2, THRB, THSD4, TIAL1, TMED2, TMTC2, TNFRSF4, TRPS1, UMPS, VAV3, XRN1
PAX3	5.20×10^{-03}	2	30	*ACTN1, AREG, CAMK2D, CCNE1, CDH10, CRACR2B, DDR1, EDIL3, EFNA1, EIF2B1, FOXM1, GBX2, GDNF, HAS2, ITGB5, MAD1L1, MED13L, NEDD8, PADI2, PFKP, PIDD1, PTCH1, RBL2, ROBO1, SHROOM3, STEAP3, THBS2, TNFRSF18, TNN, UMPS*
PAXIP1	1.60×10^{-03}	2	34	*ACTN1, AREG, CAMK2D, CD3G, CDH10, CRACR2B, DDR1, EDIL3, EFNA1, EIF2B1, FCAR, FOXM1, FZD1, GBX2, HAS2, KRT7, MAD1L1, MED13L, NEDD8, PADI2, PFKP, PIDD1, PLIN2, PTCH1, RBL2, ROBO1, SEMA3E, SERPINF1, SHROOM3, STEAP3, THBS2, THRB, TNFRSF18, UMPS*
PDCD5	7.90×10^{-03}	3	67	*ABHD2, ACTN1, ATXN10, CCNE1, CD3G, CDC45, CDH10, CLDN5, CNKSR1, CRACR2B, DDR1, DOCK10, DPM3, EDA, EDIL3, EFNA1, EIF2B1, EPB41L5, FADD, FCAR, GALNT2, GCHFR, GDNF, HAS2, HERC3, HPSE, IKBKE, IRAK4, LGR4, LRP6, MAD1L1, MAST4, MED13L, MZF1, NAV2, NCOA3, NEDD8, NELL2, PADI2, PCLO, PIDD1, PIK3R1, PLXDC2, POU2F2, PPFIA4, PTPRN2, RBFOX1, RGS10, RGS7, RMDN3, ROBO1, SEC23A, SEMA3E, SERPINF2, SHROOM3, SLC25A4, STEAP3, SUCLG2, THBS2, THRB, TIAM1, TNFRSF18, TNMD, TNN, TRHR, UMPS, UTRN*
PDCD6	1.60×10^{-03}	3	69	*ABHD2, ABR, ADCY5, ADGRV1, ANKRD17, ATP6V0A2, ATXN10, CAMK2D, CD3E, CDC45, CDH10, CHRDL2, CNRIP1, CRACR2B, DPYD, EDA, EDIL3, EIF2B1, FCAR, FOXM1, FZD1, GDNF, HAS3, HERC3, HID1, IRAK4, ITGB5, KRT7, LFNG, LGR4, MAD1L1, MED13L, mir-383, NAV2, NCOA3, NCR1, NEDD8, NELL2, P2RX2, PADI2, PCLO, PDE4D, PHF21A, PIDD1, PIK3CB, PIP5K1C, PLXDC2, PSPH, PYGB, RBL2, RMDN3, ROBO1, RPLP2, SEC23A, SGCZ, SHROOM3, SLC16A5, SLC25A4, ST8SIA1, STEAP3, THRB, THSD4, TMED2, TNFRSF18, TNFRSF4, TNN, TNR, UMPS, VAV3*
PELI2	3.50×10^{-03}	3	88	*ABLIM1, ABR, ACTN1, ADCY5, ADGRV1, ANKRD17, ANO1, AREG, ATP13A5, ATP6V0A2, CAMK2D, CCNE1, CCT6A, CD3D, CD3G, CDC45, CHMP4A, CHRDL2, CNKSR1, CNRIP1, COPA, CRACR2B, DDR1, DLG2, DPM3, DPYD, EDA, EFNA1, EIF2B1, EPB41L5, FCAR, GALNT2, GCHFR, GDNF, GNAQ, HAS2, HAS3, HERC3, HID1, IGSF9B, IKBKE, LFNG, MAD1L1, MAST4, MED13L, mir-383, NAV2, NCOA3, NCR1, NEDD8, NELL2, NLGN4X, P2RX2, PADI2, PCLO,*

				PDE4D, PDE5A, PHF21A, PIDD1, PIK3CB, PLXDC2, POU2F2, PPFIA4, PSPH, PYGB, RASGEF1B, RBFOX1, RGS7, RMDN3, SEC23IP, SERPINF2, SGCZ, SHROOM3, SLC16A5, SLC25A4, ST8SIA1, STEAP3, THRB, THSD4, TIAL1, TIAM1, TJP2, TNMD, TNN, TNR, TRPS1, UMPS, XRN1
PFKFB3	8.40×10^{-03}	3	77	*ABLIM1, ABR, ADCY5, ARSB, ATP13A5, ATP6V0A2, CAMK2D, CCNE1, CCT6A, CD3E, CDC45, CHMP2A, CHMP4A, CNKSR1, CNRIP1, COPA, CRACR2B, DDR1, DLG2, DPYD, EDIL3, EIF2B1, EPB41L5, FCAR, FOXM1, FZD1, GCHFR, GDNF, GNAQ, HERC3, HID1, HMG20B, IKBKE, IRAK4, MAD1L1, MED13L, mir-383, MZF1, NCR1, NEDD8, NLGN4X, OSBPL3, P2RX2, PADI2, PDE4D, PDE5A, PHF21A, PIDD1, PIK3R1, PIP5K1C, PKDCC, PLXDC2, PPFIA4, PSPH, RBFOX1, RGS7, RPLP2, SEC23A, SEC23IP, SEMA3E, SGCZ, SHROOM3, SLC16A5, STEAP3, SUCLG2, THBS2, THSD4, TIAL1, TIAM1, TJP2, TMED2, TNN, TNR, UMPS, UTRN, VAV3, XRN1*
PHF1	6.50×10^{-03}	2	30	*ACTN1, AREG, CAMK2D, CDH10, CRACR2B, DDR1, EDIL3, EFNA1, EIF2B1, FOXM1, GBX2, HAS2, ITGB5, MAD1L1, MED13L, MZF1, NEDD8, PADI2, PFKP, PIDD1, PIP5K1C, PTCH1, RBL2, ROBO1, SERPINA1, SHROOM3, STEAP3, THBS2, TNFRSF18, UMPS*
phosphatidylcholine	3.80×10^{-03}	3	80	*ABLIM1, ABR, ACTN1, ADCY5, ADGRV1, ANKRD17, ANO1, AREG, ARSB, ATP6V0A2, CAMK2D, CARD9, CCNE1, CCT6A, CD3D, CDC45, CHMP4A, CHRDL2, CNKSR1, CNRIP1, COPA, CRACR2B, DDR1, DLG2, DPYD, EDA, EIF2B1, FCAR, GDNF, GNAQ, GPSM1, HAS3, HERC3, HID1, HPSE, IGSF9B, IRAK4, LFNG, LGR4, MAD1L1, MED13L, mir-383, NAV2, NCOA3, NCR1, NEDD8, NELL2, NLGN4X, P2RX2, PADI2, PCLO, PDE4D, PDE5A, PHF21A, PIDD1, PIK3R1, PLXDC2, PSPH, PTCH1, PYGB, RASGEF1B, RGS7, RMDN3, SEC23IP, SGCZ, SHROOM3, SLC16A5, ST8SIA1, STEAP3, THRB, THSD4, TIAL1, TIAM1, TJP2, TNFRSF4, TNN, TNR, TRHR, UMPS, XRN1*
PIBF1	6.30×10^{-03}	3	48	*ABLIM1, ACTN1, ANO1, ATP13A5, CAMK2D, CARD9, CCNE1, CD3E, CD3G, CHMP2A, CNRIP1, CRACR2B, DDHD1, DDR1, DOCK10, DPM3, EDIL3, EIF2B1, EXOC4, GBX2, GNAQ, GPSM1, HAS3, IPO4, IRAK4, LFNG, MAD1L1, MED13L, NCOA3, NCR1, NEDD8, PADI2, PDE4D, PFKP, PIDD1, PNPLA2, POU2F2, RBFOX1, ROBO1,*

				SERPINF1, SHROOM3, SLC25A4, STEAP3, TIAL1, TJP2, TMTC2, TNFRSF18, UMPS
PIN1	1.90×10^{-03}	2	57	*ABHD2, ABLIM1, ACTN1, AREG, CAMK2D, CCT6A, CDH10, CHMP4A, CNKSR1, COPA, CRACR2B, DDR1, DLG2, EDIL3, EIF2B1, FZD1, GBX2, GDNF, GNAQ, HAS2, HAS3, HMG20B, ITGB5, LFNG, LGR4, MAD1L1, MED13L, mir-383, NCOA3, NEDD8, NELL2, NLGN4X, PADI2, PCLO, PDE5A, PFKP, PIDD1, PIK3CB, POU2F2, PTCH1, RBL2, ROBO1, RTN1, SEC23IP, SERPINF1, SGCZ, SHROOM3, SLC25A4, STEAP3, THBS2, THRB, TIAM1, TJP2, TNFRSF4, UMPS, VAV3, XRN1*
PLA2G2A	2.40×10^{-03}	3	93	*ABLIM1, ABR, ACTN1, ADCY5, ADGRV1, ANKRD17, ANO1, AREG, ARSB, ATP13A5, ATP6V0A2, CAMK2D, CCNE1, CCT6A, CD3D, CD3G, CDC45, CHMP4A, CHRDL2, CNKSR1, CNRIP1, COPA, CRACR2B, DDR1, DLG2, DPM3, DPYD, EDA, EFNA1, EIF2B1, EPB41L5, FCAR, GALNT2, GCHFR, GDNF, GNAQ, HAS2, HAS3, HERC3, HID1, HPSE, IGSF9B, IKBKE, IRAK4, LFNG, LGR4, MAD1L1, MAST4, MED13L, mir-383, NAV2, NCOA3, NCR1, NEDD8, NELL2, NLGN4X, P2RX2, PADI2, PCLO, PDE4D, PDE5A, PHF21A, PIDD1, PIK3CB, PLXDC2, POU2F2, PPFIA4, PSPH, PYGB, RASGEF1B, RBFOX1, RGS7, RMDN3, SEC23IP, SERPINF2, SGCZ, SHROOM3, SLC16A5, SLC25A4, ST8SIA1, STEAP3, THRB, THSD4, TIAL1, TIAM1, TJP2, TNMD, TNN, TNR, TRHR, TRPS1, UMPS, XRN1*
PLAGL1	6.30×10^{-03}	2	31	*ABHD2, ACTN1, AREG, CAMK2D, CDH10, CRACR2B, DDR1, EDA, EDIL3, EFNA1, EIF2B1, GBX2, HAS2, HPSE, ITGB5, MAD1L1, MED13L, NEDD8, PADI2, PFKP, PIDD1, PLIN2, PTCH1, RBL2, ROBO1, SHROOM3, STEAP3, THBS2, THRB, TNFRSF18, UMPS*
PLC	3.80×10^{-03}	3	96	*ABCC9, ABR, ACTN1, ADCY5, AKAP9, ANKRD17, ANO1, AREG, ARSB, ATP6V0A2, ATXN10, CADM2, CARD9, CCNE1, CD3D, CD3G, CHMP2A, CHMP4A, CHRDL2, CHRM3, CNKSR1, COPA, CRACR2B, DLG2, DPYD, EDA, EFNA1, EIF2B1, ENPP6, EPB41L5, F13B, FCAR, FOXP2, FZD1, GALNT2, GCC1, GCHFR, GDNF, GNAQ, HAS2, HAS3, HERC3, HID1, HMG20B, IGSF9B, IKBKE, IRAK4, MAD1L1, MAST4, MED13L, MGA, mir-383, NCR1, NEDD8, NELL2, NLGN4X, OSBPL3, P2RX2, PADI2, PCLO, PDE5A, PIDD1, PITRM1, PLIN2, PLXDC2, POU2F2, PPFIA4, PSPH, PTPRN2, PYGB, RASGEF1B, RGS10, RGS7, RMDN3, SEC23IP, SERPINF2, SGCZ, SHROOM3, SLC25A4,*

				SLC9B2, SOX8, ST8SIA1, STEAP3, TANC2, TENM3, THRB, THSD4, TIAL1, TJP2, TNMD, TNN, TNR, TRPS1, UMPS, UNC5C, XRN1
PNPLA8	9.20×10^{-03}	3	76	*ABHD2, ABR, ADCY5, ADGRV1, ANKRD17, ATP13A5, ATP6V0A2, CAMK2D, CCNE1, CCT6A, CD3E, CDH10, CHRDL2, CNRIP1, CRACR2B, DDR1, DPM3, DPYD, EDA, EDIL3, EIF2B1, FCAR, GDNF, HAS3, HID1, HPSE, IKBKE, IRAK4, KRT7, LFNG, LGR4, LMF1, MAD1L1, MAST4, MED13L, mir-383, NAV2, NCOA3, NCR1, NEDD8, OSBPL3, P2RX2, PADI2, PCLO, PDE4D, PHF21A, PIDD1, PIK3CB, PIP5K1C, PLXDC2, PSPH, PTCH1, PYGB, RBFOX1, RGS7, ROBO1, RPLP2, SEC23A, SEMA3E, SGCZ, SHROOM3, SLC16A5, SLC25A4, ST8SIA1, STEAP3, THRB, THSD4, TIAM1, TMED2, TNFRSF18, TNFRSF4, TNN, TNR, TRHR, UMPS, VAV3*
poly(ADP-ribose)	6.90×10^{-03}	3	30	*ABHD2, ABLIM1, AREG, CCNE1, CHMP4A, COPA, DLG2, DOCK10, EFNA1, GNAQ, HAS2, IKBKE, ITGB5, KRT7, NCOA3, NELL2, NLGN4X, PTCH1, RBL2, RGS10, ROBO1, SEC23IP, SLC25A4, THBS2, THRB, TJP2, TNFRSF18, UTRN, VAV3, XRN1*
PPP6C	9.60×10^{-03}	3	63	*ABHD2, ABLIM1, ADGRV1, AREG, ARSB, CAMK2D, CCNE1, CCT6A, CD3D, CD3E, CDC45, CHMP2A, CHMP4A, CHRDL2, CLDN5, COPA, CRACR2B, DLG2, EIF2B1, FADD, FZD1, GNAQ, HAS3, HMG20B, HPSE, IGSF9B, ITGB5, LFNG, LGR4, LRP6, MAD1L1, MED13L, NCOA3, NEDD8, NELL2, NLGN4X, OSBPL3, PADI2, PFKP, PIDD1, PIK3R1, PLIN2, PLXDC2, RASGEF1B, RBL2, RGS7, ROBO1, SEC23IP, SERPINF1, SHROOM3, SLC25A4, STEAP3, THRB, TJP2, TNFRSF18, TNFRSF4, TNN, TRHR, TRPS1, UMPS, UTRN, VAV3, XRN1*
pregnenolone sulfate	7.20×10^{-03}	3	31	*ACTN1, AREG, ARSB, ATXN10, CAMK2D, CCNE1, CD3D, CDH10, CLDN5, DOCK10, EDIL3, EFNA1, FADD, FZD1, HAS2, HAS3, IGSF9B, LRP6, MAD1L1, NELL2, OSBPL3, PCLO, PDE5A, PIK3R1, PLIN2, PLXDC2, POU2F2, RASGEF1B, ROBO1, TNN, TRPS1*
PRKAR2A	2.30×10^{-03}	3	57	*ACTN1, ADGRV1, AKAP9, AREG, ATP13A5, ATP6V0D2, CAMK2D, CD3D, CDC45, CDH10, CRACR2B, DDR1, EDIL3, EIF2B1, FADD, FZD1, GALNT2, GBX2, GDNF, HPSE, IGSF9B, IRAK4, LFNG, LRP6, MAD1L1, MED13L, NEDD8, OSBPL3, PADI2, PCLO, PDE4D, PDE5A, PIDD1, PIK3R1, PLXDC2, PNPLA2, POU2F2, PSPH, RASGEF1B, RBFOX1, RBL2, RGS10, ROBO1, SERPINA1, SERPINF1, SEZ6L,*

				SHROOM3, SLC25A4, SOX8, STEAP3, THBS2, TIAM1, TMTC2, TNFRSF18, TNFRSF4, TRPS1, UMPS
PRKD2	9.60 × 10^{-03}	3	84	*ABR, ADCY5, ADGRV1, AREG, ATP13A5, ATXN10, CARD9, CCT6A, CD3D, CDC45, CHMP2A, CHRDL2, CLDN5, CNKSR1, CNRIP1, CRACR2B, DDR1, DOCK10, DPYD, EDIL3, EFNA1, EIF2B1, EPB41L5, FADD, GALNT2, GCC1, GCHFR, GDNF, GNAQ, HAS2, HERC3, HID1, IGSF9B, IKBKE, IRAK4, ITGB5, LGR4, MAST4, MED13L, mir-383, NAV2, NCOA3, NCR1, NEDD8, NELL2, OSBPL3, P2RX2, PADI2, PCLO, PDE5A, PIDD1, PIK3CB, PIP5K1C, PLXDC2, PNPLA2, POU2F2, PPFIA4, PSPH, PYGB, RASGEF1B, RBFOX1, RGS7, RMDN3, ROBO1, SEC23A, SERPINA1, SERPINF2, SHROOM3, SLC16A5, ST8SIA1, STEAP3, THSD4, TIAM1, TJP2, TMED2, TNFRSF18, TNMD, TNN, TNR, TRHR, TRPS1, UMPS, UTRN, VAV3*
protein phosphatase	9.90 × 10^{-03}	2	27	*ABHD2, ABLIM1, AREG, CCNE1, CHMP4A, COPA, DLG2, EFNA1, FOXM1, GNAQ, IKBKE, ITGB5, KRT7, NCOA3, NELL2, NLGN4X, PCLO, PDE4D, PTCH1, RBL2, ROBO1, SEC23IP, THRB, TJP2, TNFRSF18, VAV3, XRN1*
PSENEN	4.10 × 10^{-03}	3	75	*ACTN1, ADGRV1, AREG, ATP6V0D2, ATXN10, CAMK2D, CD3E, CD3G, CDC45, CDH10, CRACR2B, DDR1, DPYD, EDA, EDIL3, EFNA1, EIF2B1, FADD, FCAR, FOXM1, FZD1, GALNT2, GBX2, GCHFR, GDNF, HAS2, HAS3, HERC3, HPSE, IRAK4, ITGB5, LGR4, LRP6, MAD1L1, MAST4, MED13L, MZF1, NAV2, NCOA3, NCR1, NEDD8, NELL2, PADI2, PCLO, PFKP, PHF21A, PIDD1, PIK3CB, PNPLA2, POU2F2, PPFIA4, PTCH1, PTPRN2, PYGB, RBFOX1, RBL2, RGS10, RMDN3, ROBO1, RSL1D1, SEC23A, SEMA3E, SERPINF2, SHROOM3, SLC25A4, STEAP3, SUCLG2, THBS2, TIAM1, TNFRSF18, TNN, TRHR, TSSK4, UMPS, UTRN*
PTGES	2.30 × 10^{-03}	3	69	*ACTN1, ADGRV1, ATP13A5, ATP6V0D2, ATXN10, CAMK2D, CARD9, CD3D, CDC45, CLDN5, CRACR2B, DDR1, DOCK10, EDIL3, EIF2B1, FADD, FCAR, FOXM1, FZD1, GBX2, GNAQ, GPSM1, HAS3, HPSE, IKBKE, IRAK4, LFNG, LGR4, LRP6, MAD1L1, MAST4, MED13L, MZF1, NAV2, NEDD8, NELL2, OSBPL3, PADI2, PCLO, PFKP, PHF21A, PIDD1, PIK3R1, PIP5K1C, PLIN2, PNPLA2, POU2F2, PTPRN2, RBL2, RGS10, RGS7, ROBO1, SEC23A, SEMA3E,*

				SERPINA1, SERPINF1, SHROOM3, SLC25A4, STEAP3, SUCLG2, THRB, THSD4, TIAL1, TIAM1, TJP2, TMED2, TMTC2, UMPS, UTRN
PYHIN1	9.20×10^{-03}	2	28	*ACTN1, AREG, CAMK2D, CCNE1, CDH10, CRACR2B, DDR1, EDIL3, EFNA1, EIF2B1, FOXM1, GBX2, HAS2, ITGB5, MAD1L1, MED13L, NEDD8, PADI2, PFKP, PIDD1, PTCH1, RBL2, ROBO1, SHROOM3, STEAP3, THBS2, TNFRSF18, UMPS*
RANBP2	6.10×10^{-03}	3	68	*ABHD2, ABR, ADCY5, ADGRV1, ANKRD17, ARSB, ATP6V0A2, CAMK2D, CCNE1, CD3E, CDH10, CHRDL2, CNRIP1, CRACR2B, DDR1, DPYD, EDA, EDIL3, EIF2B1, FCAR, FZD1, GDNF, HAS3, HID1, HPSE, IKBKE, IRAK4, KRT7, LFNG, LGR4, MAD1L1, MED13L, mir-383, NAV2, NCOA3, NCR1, NEDD8, OSBPL3, P2RX2, PADI2, PCLO, PDE4D, PHF21A, PIDD1, PIK3CB, PLXDC2, PSPH, PYGB, RGS7, ROBO1, RPLP2, SEMA3E, SGCZ, SHROOM3, SLC16A5, SLC25A4, ST8SIA1, STEAP3, THRB, THSD4, TIAM1, TNFRSF18, TNFRSF4, TNN, TNR, TRHR, UMPS, VAV3*
RCHY1	7.00×10^{-04}	3	86	*ABLIM1, ACTN1, ADGRV1, AREG, ARSB, ATP13A5, ATP6V0D2, CAMK2D, CCT6A, CD3D, CD3E, CD3G, CDH10, CHMP4A, CNKSR1, COPA, CRACR2B, DDR1, DLG2, DPYD, EDA, EDIL3, EFNA1, EIF2B1, FADD, FCAR, FOXM1, GALNT2, GBX2, GCHFR, GNAQ, HAS2, HAS3, HMG20B, HPSE, IGSF9B, IRAK4, ITGB5, LFNG, LGR4, LRP6, MAD1L1, MED13L, NCR1, NEDD8, NELL2, NLGN4X, PADI2, PCLO, PDE4D, PDE5A, PFKP, PHF21A, PIDD1, PIK3CB, PLXDC2, PNPLA2, POU2F2, PPFIA4, PTCH1, PYGB, RASGEF1B, RBFOX1, RBL2, RGS10, RGS7, ROBO1, RSL1D1, SEC23IP, SEMA3E, SERPINA1, SERPINF1, SERPINF2, SHROOM3, SLC25A4, STEAP3, TEAD4, TENM3, TJP2, TNFRSF18, TRHR, TRPS1, TSSK4, UMPS, VAV3, XRN1*
RECQL5	7.60×10^{-03}	3	30	*ACTN1, AREG, ATXN10, CAMK2D, CDH10, CRACR2B, DDR1, EDIL3, EFNA1, EIF2B1, FOXM1, GBX2, HAS2, ITGB5, MAD1L1, MED13L, NEDD8, NELL2, PADI2, PFKP, PIDD1, PTCH1, RBL2, ROBO1, SHROOM3, STEAP3, THBS2, TNFRSF18, TRHR, UMPS*
RNF2	5.60×10^{-03}	2	29	*ACTN1, AREG, CAMK2D, CDH10, CRACR2B, DDR1, EDIL3, EFNA1, EIF2B1, FOXM1, GBX2, HAS2, ITGB5, MAD1L1, MED13L, NEDD8, PADI2, PFKP, PIDD1, PTCH1, RBL2, ROBO1, SERPINA1, SHROOM3, SMC1B, STEAP3, THBS2, TNFRSF18, UMPS*

Rp-8-Br-cGMPS	5.40 × 10^{-03}	3	48	*ACTN1, ADGRV1, AREG, ATP13A5, CAMK2D, CD3D, CDH10, CLDN5, CRACR2B, DDR1, DOCK10, EDIL3, EFNA1, EIF2B1, FOXM1, FZD1, GBX2, IGSF9B, IKBKE, IRAK4, ITGB5, LFNG, MAD1L1, MED13L, NEDD8, OSBPL3, PADI2, PCLO, PDE5A, PFKP, PIDD1, PLIN2, PLXDC2, POU2F2, PSPH, RASGEF1B, RBFOX1, RBL2, ROBO1, SERPINA1, SERPINF1, SHROOM3, STEAP3, THBS2, TNFRSF18, TNN, TRPS1, UMPS*
RPS3	9.20 × 10^{-03}	3	81	*ABHD2, ABR, ADCY5, ADGRV1, ANKRD17, ATP6V0A2, CAMK2D, CCNE1, CD3G, CDC45, CDH10, CHRDL2, CNKSR1, CNRIP1, CRACR2B, DDR1, DPM3, DPYD, EDA, EDIL3, EFNA1, EIF2B1, EPB41L5, FCAR, GALNT2, GCHFR, GDNF, HAS2, HAS3, HERC3, HID1, HPSE, IKBKE, IRAK4, LFNG, LGR4, MAD1L1, MAST4, MED13L,* mir-383, *NAV2, NCOA3, NCR1, NEDD8, P2RX2, PADI2, PCLO, PDE4D, PHF21A, PIDD1, PIK3CB, PIK3R1, PIP5K1C, PLXDC2, POU2F2, PPFIA4, PSPH, PYGB, RGS7, RMDN3, ROBO1, RPLP2, SEC23A, SERPINF2, SGCZ, SHROOM3, SLC16A5, SLC25A4, ST8SIA1, STEAP3, THRB, THSD4, TIAM1, TMED2, TNFRSF18, TNMD, TNN, TNR, TRHR, UMPS, VAV3*
RPS6KB1	8.30 × 10^{-03}	2	44	*ACTN1, AREG, ATP13A5, ATP6V0A2, ATP6V0D2, CAMK2D, CCNE1, CD3D, CDC45, CRACR2B, DDR1, EFNA1, EIF2B1, GBX2, HAS2, HAS3, IGSF9B, IKBKE, ITGB5, MAD1L1, MED13L, NEDD8, OSBPL3, PADI2, PCLO, PIDD1, PIK3CB, PLXDC2, POU2F2, PTCH1, PYGB, RASGEF1B, RBFOX1, RBL2, RPLP2, SERPINA1, SHROOM3, SLC25A4, STEAP3, THBS2, TNFRSF18, TRPS1, UMPS, UTRN*
RUNX3	7.10 × 10^{-03}	2	35	*ACTN1, CAMK2D, CD3D, CD3E, CDH10, CLDN5, CRACR2B, DDR1, EDIL3, EFNA1, EIF2B1, FOXM1, GBX2, GCHFR, GNAQ, HAS2, ITGB5, MAD1L1, MED13L, NEDD8, PADI2, PFKP, PIDD1, PPFIA4, PTCH1, RBL2, ROBO1, RTN1, SERPINA1, SHROOM3, STEAP3, THBS2, TJP2, TNFRSF18, UMPS*
S100A6	4.50 × 10^{-03}	2	30	*ACTN1, AREG, CAMK2D, CCNE1, CDH10, CRACR2B, DDR1, EDIL3, EFNA1, EIF2B1, FOXM1, GBX2, GDNF, HAS2, ITGB5, MAD1L1, MED13L, NEDD8, PADI2, PFKP, PIDD1, PTCH1, RBL2, ROBO1, SHROOM3, STEAP3, THBS2, TNFRSF18, TNN, UMPS*
S100B	7.70 × 10^{-03}	3	81	*ABCC9, ABLIM1, ABR, ACTN1, ADCY5, ANO1, ARSB, ATP13A5, ATP6V0A2, ATXN10, CAMK2D, CCNE1, CD3G, CDH10, CHMP4A, CHRDL2, CLDN5, CNKSR1, CNRIP1, COPA, CRACR2B, DDHD1,*

				DDR1, DLG2, DPM3, EDIL3, EIF2B1, EPB41L5, FADD, FCAR, FSCN2, GBX2, GNAQ, GPSM1, HMCN1, HMG20B, IKBKE, IRAK4, ITGB5, LMF1, MAD1L1, MED13L, MGA, mir-383, NCR1, NEDD8, NLGN4X, OSBPL3, P2RX2, PADI2, PDE4D, PFKP, PIDD1, PKDCC, POU2F2, PTCH1, RBFOX1, RBL2, RGS10, RGS7, ROBO1, RPLP2, RSL1D1, SEC23IP, SERPINA1, SERPINF2, SGCZ, SHROOM3, SLC16A5, STEAP3, TENM3, THBS2, TMTC2, TNFRSF18, TNMD, TNN, TNR, TSSK4, UMPS, UTRN, XRN1
S-adenosylhomocysteine	1.10×10^{-03}	3	39	*ABHD2, ABLIM1, AREG, CCNE1, CD3D, CD3E, CHMP4A, CLDN5, COPA, DLG2, EFNA1, FZD1, GDNF, GNAQ, HAS2, IKBKE, ITGB5, KRT7, LRP6, NCOA3, NELL2, NLGN4X, PFKP, PTCH1, RBL2, RGS10, ROBO1, SEC23IP, SERPINF1, SLC25A4, ST8SIA1, THBS2, THRB, THSD4, TJP2, TNFRSF18, UTRN, VAV3, XRN1*
SFRP2	6.10×10^{-03}	3	71	*ABHD2, ABR, ADCY5, ADGRV1, ANKRD17, ATP6V0A2, ATXN10, CAMK2D, CCNE1, CD3E, CDH10, CHRDL2, CNRIP1, CRACR2B, DDR1, DPYD, EDA, EDIL3, EIF2B1, FCAR, GDNF, HAS3, HID1, HPSE, IKBKE, IRAK4, KRT7, LFNG, LGR4, MAD1L1, MED13L, mir-383, NAV2, NCOA3, NCR1, NEDD8, NELL2, P2RX2, PADI2, PCLO, PDE4D, PHF21A, PIDD1, PIK3CB, PIP5K1C, PLXDC2, PSPH, PTCH1, PYGB, RGS7, ROBO1, RPLP2, SEC23A, SEMA3E, SGCZ, SHROOM3, SLC16A5, SLC25A4, ST8SIA1, STEAP3, THRB, THSD4, TIAM1, TMED2, TNFRSF18, TNFRSF4, TNN, TNR, TRHR, UMPS, VAV3*
SIAH1	4.80×10^{-03}	3	72	*ABLIM1, ABR, ADCY5, ADGRV1, ARSB, ATP6V0A2, CD3E, CD3G, CHMP4A, CHRDL2, CNKSR1, CNRIP1, COPA, DLG2, DPYD, EDA, EDIL3, ENPP6, F13B, FCAR, GCHFR, GDNF, GNAQ, HAS2, HERC3, HID1, HPSE, LFNG, MAD1L1, mir-383, MZF1, NAV2, NCR1, NELL2, NLGN4X, OSBPL3, P2RX2, PDE4D, PIK3CB, PLXDC2, PPFIA4, PSPH, PTCH1, RASGEF1B, RGS7, RMDN3, RPLP2, SEC23A, SEC23IP, SEMA3E, SERPINF1, SERPINF2, SEZ6L, SGCZ, SLC16A5, SLC22A23, SLC25A4, TANC2, TEAD4, THRB, THSD4, TIAM1, TJP2, TMED2, TMTC2, TNFRSF4, TNN, TNR, TRHR, TRPS1, TWF1, XRN1*
SIAH2	8.70×10^{-03}	3	88	*ABCC9, ABR, ACTN1, ADCY5, ADGRV1, ANKRD17, AREG, ARSB, ATP13A5, ATP6V0A2, ATXN10, CAMK2D, CCNE1, CD3D, CD3G, CDH10, CHMP2A, CHMP4A, CHRDL2, CNRIP1, COPA, CRACR2B, DDR1, DLG2, DPYD, EDA, EDIL3, EFNA1, EIF2B1, FOXM1, GALNT2,*

				GBX2, GCHFR, GNAQ, HAS2, HAS3, HERC3, HID1, HPSE, IGSF9B, IKBKE, IRAK4, ITGB5, LFNG, MAD1L1, MED13L, mir-383, NEDD8, NLGN4X, P2RX2, PADI2, PCLO, PDE4D, PFKP, PIDD1, PIK3CB, PIK3R1, PIP5K1C, PPFIA4, PSPH, RASGEF1B, RBFOX1, RBL2, RGS10, RMDN3, ROBO1, RPLP2, SEC23IP, SEMA3E, SGCZ, SHROOM3, SLC16A5, ST8SIA1, STEAP3, THBS2, THSD4, TJP2, TMED2, TNFRSF18, TNFRSF4, TNMD, TNN, TNR, TRPS1, TSSK4, UBE2M, UMPS, XRN1
SIGIRR	8.20×10^{-03}	3	94	*ABLIM1, ABR, ADCY5, ADGRV1, ANKRD17, ATP13A5, ATP6V0A2, CCNE1, CCT6A, CD3G, CDC45, CHMP4A, CHRDL2, CNKSR1, CNRIP1, COPA, CRACR2B, DDHD1, DDR1, DLG2, DPM3, DPYD, EDA, EDIL3, EFNA1, EIF2B1, EPB41L5, FCAR, GALNT2, GCC1, GCHFR, GDNF, GNAQ, HAS2, HERC3, HID1, HMCN1, HMG20B, HPSE, IKBKE, IRAK4, LFNG, LGR4, MAST4, MED13L, mir-383, MZF1, NAV2, NCOA3, NCR1, NEDD8, NELL2, NLGN4X, P2RX2, PADI2, PCLO, PDE4D, PDE5A, PFKP, PHF21A, PIDD1, PIK3CB, PIK3R1, PIP5K1C, PLXDC2, POU2F2, PPFIA4, PSPH, PYGB, RBFOX1, RGS7, RMDN3, ROBO1, RPLP2, SEC23IP, SERPINA1, SERPINF2, SGCZ, SHROOM3, SLC16A5, ST8SIA1, STEAP3, SUCLG2, THRB, THSD4, TIAM1, TJP2, TMED2, TNMD, TNN, TNR, TRHR, UMPS, XRN1*
SKI	9.50×10^{-03}	2	29	*ACTN1, AREG, CAMK2D, CCNE1, CDH10, CRACR2B, DDR1, EDIL3, EFNA1, EIF2B1, FOXM1, GBX2, HAS2, ITGB5, MAD1L1, MED13L, NEDD8, PADI2, PFKP, PIDD1, PNPLA2, PTCH1, RBL2, ROBO1, SHROOM3, STEAP3, THBS2, TNFRSF18, UMPS*
SLK	5.80×10^{-03}	3	84	*ABR, ADCY5, ADGRV1, ANKRD17, ANO1, ATP13A5, ATP6V0A2, ATXN10, CAMK2D, CARD9, CCT6A, CD3D, CD3E, CD3G, CHMP4A, CHRDL2, CNRIP1, COPA, CRACR2B, DDR1, DLG2, DPM3, DPYD, EDA, EFNA1, EIF2B1, FSCN2, GALNT2, GCHFR, GDNF, GNAQ, GPAT3, GPSM1, HAS2, HERC3, HID1, IGSF9B, LFNG, LMF1, LRP6, MED13L, MZF1, NCOA3, NCR1, NEDD8, NELL2, NLGN4X, P2RX2, PADI2, PHF21A, PIDD1, PIK3CB, PIK3R1, PLXDC2, PPFIA4, PSPH, PTCH1, PYGB, RASGEF1B, RBFOX1, RMDN3, RPLP2, RSL1D1, SEC23IP, SERPINF2, SGCZ, SHROOM3, SLC16A5, SOX8, ST8SIA1, STEAP3, SUCLG2, TENM3, THRB, THSD4, TIAL1, TIAM1, TMTC2, TNN, TNR, TRHR, TRPS1, UMPS, XRN1*

SMAD1	8.30×10^{-03}	2	30	*ACTN1, AREG, CAMK2D, CCNE1, CDH10, CLDN5, CRACR2B, DDR1, EDIL3, EFNA1, EIF2B1, FADD, FOXM1, GBX2, HAS2, ITGB5, MAD1L1, MED13L, NEDD8, PADI2, PFKP, PIDD1, PTCH1, RBL2, ROBO1, SHROOM3, STEAP3, THBS2, TNFRSF18, UMPS*
Smad2/3-Smad4	8.60×10^{-03}	2	13	*AREG, CCNE1, CCT6A, HAS2, HMG20B, ITGB5, LGR4, LRP6, PIK3CB, PIK3R1, POU2F2, SLC25A4, SOX8*
SMARCA2	9.30×10^{-03}	3	67	*ABHD2, ABLIM1, ADGRV1, ANO1, AREG, CAMK2D, CCNE1, CD3D, CDC45, CDH10, CHMP4A, COPA, CRACR2B, DDR1, DLG2, EDA, EDIL3, EFNA1, EIF2B1, EPB41L5, FADD, FOXM1, GBX2, GNAQ, HPSE, IGSF9B, IKBKE, KRT7, LFNG, LRP6, MAD1L1, MAST4, MED13L, NAV2, NCOA3, NEDD8, NELL2, NLGN4X, PADI2, PDE5A, PIDD1, PIK3CB, PLXDC2, PTCH1, RASGEF1B, RBL2, ROBO1, RSL1D1, SEC23IP, SERPINA1, SERPINF1, SERPINF2, SHROOM3, STEAP3, TANC2, THBS2, THRB, THSD4, TJP2, TNFRSF18, TNFRSF4, TNN, TRPS1, UMPS, UTRN, VAV3, XRN1*
SMARCA4	7.20×10^{-03}	3	89	*ABHD2, ABLIM1, ACTN1, ANO1, AREG, ATP13A5, ATXN10, BEND5, CAMK2D, CCNE1, CCT6A, CD3D, CD3E, CDC45, CDH10, CHMP4A, CNKSR1, COPA, CRACR2B, DDR1, DLG2, DPYD, EDIL3, EIF2B1, EPB41L5, GALNT2, GBX2, GCHFR, GNAQ, HAS2, HERC3, HMG20B, IGSF9B, IKBKE, IRAK4, ITGB5, KRT7, MAD1L1, MAST4, MED13L, MZF1, NAV2, NCOA3, NCR1, NEDD8, NELL2, NLGN4X, PADI2, PDE4D, PDE5A, PFKP, PIDD1, PIK3R1, PIP5K1C, PLIN2, PLXDC2, PPFIA4, PTCH1, PYGB, RASGEF1B, RBFOX1, RBL2, RGS10, RGS7, RMDN3, ROBO1, RSL1D1, SEC23A, SEC23IP, SERPINF2, SHROOM3, SLC25A4, STEAP3, STXBP6, SUCLG2, TANC2, TEAD4, THBS2, THRB, THSD4, TJP2, TNFRSF4, TRHR, TSSK4, TWF1, UMPS, VAV3, XRN1, ZNF280B*
SMN1/SMN2	7.80×10^{-03}	3	28	*ACTN1, AREG, CAMK2D, CDH10, CRACR2B, DDR1, EDIL3, EFNA1, EIF2B1, FOXM1, GBX2, GEMIN2, HAS2, ITGB5, MAD1L1, MED13L, NEDD8, PADI2, PFKP, PIDD1, PTCH1, RBL2, ROBO1, SHROOM3, STEAP3, THBS2, TNFRSF18, UMPS*
SMYD3	9.00×10^{-03}	2	24	*ABHD2, ABLIM1, AREG, CHMP4A, COPA, DLG2, EFNA1, FOXM1, GNAQ, IKBKE, KRT7, NCOA3, NELL2, NLGN4X, PIK3CB, PTCH1, RBL2, ROBO1, SEC23IP, THRB, TJP2, TNFRSF18, VAV3, XRN1*
Sox	7.80×10^{-03}	3	58	*ABCC9, ABHD2, ABLIM1, ACTN1, ANKRD17, ATP6V0D2, CAMK2D, CDC45, CDH10, CHMP4A, CNKSR1, CNRIP1, COPA, CRACR2B,*

				DDR1, DLG2, EDIL3, EFNA1, EIF2B1, FZD1, GNAQ, IRAK4, LRP6, MAD1L1, MED13L, mir-383, NCOA3, NEDD8, NELL2, NLGN4X, PADI2, PCLO, PFKP, PIDD1, PIK3R1, PIP5K1C, PITRM1, PLIN2, SEC23A, SEC23IP, SEMA3E, SERPINA1, SGCZ, SHROOM3, SLC25A4, SOX8, ST8SIA1, STEAP3, THRB, TIAM1, TJP2, TMED2, TNFRSF18, TRHR, UMPS, UTRN, VAV3, XRN1
SOX2-OCT4	4.30×10^{-03}	3	34	*ACTN1, AREG, CAMK2D, CCNE1, CDH10, CRACR2B, DDR1, EDIL3, EFNA1, EIF2B1, FADD, FOXM1, GBX2, HAS2, HPSE, ITGB5, KRT7, MAD1L1, MED13L, NEDD8, PADI2, PFKP, PIDD1, PIK3R1, PTCH1, RBL2, ROBO1, SERPINA1, SERPINF1, SHROOM3, STEAP3, TNFRSF18, TRPS1, UMPS*
SP100	6.40×10^{-03}	2	28	*ACTN1, AREG, CAMK2D, CCNE1, CDH10, CRACR2B, DDR1, EDIL3, EFNA1, EIF2B1, FOXM1, GBX2, HAS2, ITGB5, MAD1L1, MED13L, NEDD8, PADI2, PFKP, PIDD1, PTCH1, RBL2, ROBO1, SHROOM3, STEAP3, THBS2, TNFRSF18, UMPS*
SPATA2	3.70×10^{-03}	3	70	*ABLIM1, ABR, ADCY5, ADGRV1, ATP6V0A2, CAMK2D, CCNE1, CD3E, CDC45, CDH10, CHMP4A, CHRDL2, CNRIP1, COPA, CRACR2B, DDR1, DLG2, DPYD, EDA, EDIL3, EIF2B1, FCAR, GDNF, GNAQ, HAS3, HID1, HPSE, IRAK4, LFNG, LGR4, MAD1L1, MED13L, mir-383, NAV2, NCOA3, NCR1, NEDD8, NELL2, NLGN4X, P2RX2, PADI2, PCLO, PDE4D, PIDD1, PIK3CB, PIK3R1, PLXDC2, PSPH, PTCH1, PYGB, RGS7, ROBO1, RPLP2, SEC23IP, SEMA3E, SGCZ, SHROOM3, SLC16A5, SLC25A4, ST8SIA1, STEAP3, THRB, THSD4, TJP2, TNFRSF4, TNN, TNR, TRHR, UMPS, XRN1*
SPN	6.60×10^{-03}	2	29	*ACTN1, AREG, CAMK2D, CD3E, CDH10, CLDN5, CRACR2B, DDR1, EDIL3, EFNA1, EIF2B1, FOXM1, GBX2, HAS2, ITGB5, MAD1L1, MED13L, NEDD8, PADI2, PFKP, PIDD1, PTCH1, RBL2, ROBO1, SHROOM3, STEAP3, THBS2, TNFRSF18, UMPS*
SPSB1	2.90×10^{-03}	3	78	*ABLIM1, ACTN1, ADGRV1, ANO1, ARSB, ATP13A5, ATP6V0D2, CAMK2D, CCNE1, CCT6A, CD3D, CD3E, CDC45, CHMP4A, CHRM3, CNKSR1, CNRIP1, COPA, CRACR2B, DDR1, DLG2, DOCK10, DPM3, DPYD, EIF2B1, FADD, FCAR, GDNF, GEMIN2, GNAQ, HAS2, HAS3, HERC3, IGSF9B, IRAK4, ITGB5, KCNJ8, LFNG, LRP6, MAD1L1, MED13L, mir-383, NAV2, NEDD8, NELL2, NLGN4X, PADI2, PCLO, PDE5A, PIDD1, PIK3CB, PIK3R1, PIP5K1C, PLXDC2, RASGEF1B, RBFOX1, RBL2, RGS7, RMDN3, RPLP2, SEC23A, SEC23IP,*

				SHROOM3, SLC25A4, SLC9B2, SOX8, ST8SIA1, STEAP3, TEAD4, TIAL1, TJP2, TMED2, TNN, TRHR, TRPS1, UMPS, VAV3, XRN1
SRSF2	6.50×10^{-03}	2	28	*ABCC9, ACTN1, AREG, CAMK2D, CDH10, CRACR2B, DDR1, EDIL3, EFNA1, EIF2B1, FOXM1, GBX2, HAS2, ITGB5, MAD1L1, MED13L, NEDD8, PADI2, PFKP, PIDD1, PTCH1, RBL2, ROBO1, SHROOM3, STEAP3, THBS2, TNFRSF18, UMPS*
SRSF3	3.70×10^{-03}	1	2	*CCNE1, FOXM1*
SUV39H1	4.70×10^{-03}	2	29	*ACTN1, AREG, CAMK2D, CCNE1, CDC45, CDH10, CRACR2B, DDR1, EDIL3, EFNA1, EIF2B1, FOXM1, GBX2, HAS2, ITGB5, MAD1L1, MED13L, NEDD8, PADI2, PFKP, PIDD1, PTCH1, RBL2, ROBO1, SHROOM3, STEAP3, THBS2, TNFRSF18, UMPS*
TACC3	8.70×10^{-03}	3	77	*ACTN1, ADGRV1, ANO1, AREG, ARSB, ATP13A5, ATP6V0D2, BEND5, CAMK2D, CCT6A, CD3D, CD3G, CDH10, CRACR2B, DDR1, DPYD, EDA, EDIL3, EFNA1, EIF2B1, FADD, FCAR, FOXM1, GALNT2, GBX2, HAS2, HAS3, HERC3, HPSE, IGSF9B, IRAK4, ITGB5, LFNG, LGR4, LRP6, MAD1L1, MED13L, NCOA3, NCR1, NEDD8, OSBPL3, PADI2, PCLO, PFKP, PHF21A, PIDD1, PIK3CB, PIK3R1, PLXDC2, PPFIA4, PSPH, PTCH1, PYGB, RASGEF1B, RBFOX1, RBL2, RGS10, RGS7, RMDN3, ROBO1, SEMA3E, SHROOM3, SLC25A4, STEAP3, STXBP6, THBS2, TIAL1, TIAM1, TNFRSF18, TNFRSF4, TNN, TRHR, TRPS1, TSSK4, TWF1, UMPS, VAV3*
TADA3	7.50×10^{-03}	2	28	*ACTN1, AREG, CAMK2D, CCNE1, CDH10, CRACR2B, DDR1, EDIL3, EFNA1, EIF2B1, FOXM1, GBX2, HAS2, ITGB5, MAD1L1, MED13L, NEDD8, PADI2, PFKP, PIDD1, PTCH1, RBL2, ROBO1, SHROOM3, STEAP3, THBS2, TNFRSF18, UMPS*
TAF1	2.40×10^{-03}	3	86	*ABLIM1, ACTN1, ADGRV1, AREG, ARSB, ATP13A5, ATP6V0D2, CAMK2D, CD3D, CD3E, CD3G, CDH10, CHMP4A, COPA, CRACR2B, DDR1, DLG2, DPYD, EDA, EDIL3, EFNA1, EIF2B1, FADD, FCAR, FOXM1, FZD1, GALNT2, GBX2, GCHFR, GNAQ, HAS2, HAS3, HERC3, HPSE, IGSF9B, IRAK4, ITGB5, LFNG, LGR4, LRP6, MAD1L1, MED13L, NCOA3, NCR1, NEDD8, NELL2, NLGN4X, PADI2, PCLO, PDE4D, PDE5A, PFKP, PHF21A, PIDD1, PIK3CB, PLXDC2, PNPLA2, POU2F2, PPFIA4, PTCH1, PYGB, RASGEF1B, RBFOX1, RBL2, RGS10, RGS7, RMDN3, ROBO1, RSL1D1, SEC23IP, SEMA3E, SERPINF2, SHROOM3, SLC25A4, STEAP3, THBS2, TIAM1, TJP2, TNFRSF18, TNN, TRHR, TRPS1, TSSK4, UMPS, VAV3, XRN1*

TCF7	4.10×10^{-03}	2	17	*CD3D, CD3E, CD3G, FCAR, FZD1, GALNT2, GBX2, GPAT3, HAS2, ITGB5, KRT7, PLIN2, SEMA3E, SERPINF1, SLC16A5, THRB, TJP2*
TFIIH	2.30×10^{-03}	3	70	*ABHD2, ABLIM1, ADGRV1, ANKRD17, AREG, ARSB, ATP13A5, ATP6V0D2, CARD9, CCNE1, CD3D, CDC45, CHMP4A, COPA, DLG2, DOCK10, EDA, EFNA1, EPB41L5, FCAR, FOXM1, GNAQ, GPSM1, HERC3, HPSE, IGSF9B, IKBKE, IRAK4, KRT7, LFNG, LGR4, MAST4, mir-383, NCOA3, NCR1, NELL2, NLGN4X, PCLO, PDE5A, PHF21A, PIK3CB, PIK3R1, PIP5K1C, PLIN2, PLXDC2, PNPLA2, PTCH1, RASGEF1B, RBFOX1, RBL2, RGS10, RMDN3, ROBO1, SEC23A, SEC23IP, SERPINA1, SERPINF1, SGCZ, SLC25A4, TANC2, THRB, TIAL1, TJP2, TMED2, TMTC2, TNFRSF18, TRHR, TRPS1, VAV3, XRN1*
TFIIH	5.50×10^{-03}	2	29	*ABHD2, ABLIM1, AREG, CCNE1, CHMP4A, COPA, DLG2, EFNA1, FOXM1, GNAQ, IKBKE, IRAK4, KRT7, NCOA3, NELL2, NLGN4X, PCLO, PLIN2, PTCH1, RBL2, ROBO1, SEC23IP, SERPINA1, SERPINF1, THRB, TJP2, TNFRSF18, VAV3, XRN1*
thiamine	2.90×10^{-03}	3	54	*ACTN1, AREG, ARSB, ATXN10, CAMK2D, CDC45, CDH10, CLDN5, CNRIP1, CRACR2B, DDR1, DOCK10, EDIL3, EFNA1, EIF2B1, FADD, FZD1, GBX2, GDNF, HAS2, HAS3, HPSE, IKBKE, ITGB5, MAST4, MED13L, MZF1, NAV2, NCOA3, NCR1, NEDD8, NELL2, OSBPL3, PADI2, PCLO, PFKP, PIDD1, PIK3R1, PLIN2, PNPLA2, PTCH1, PTPRN2, RBFOX1, RBL2, RGS7, ROBO1, SEC23A, SHROOM3, STEAP3, SUCLG2, TNFRSF18, TNN, UMPS, UTRN*
THPO	7.50×10^{-03}	3	94	*ABCC9, ABR, ACTN1, AKAP9, AREG, ARSB, ATP13A5, ATP6V0A2, ATP6V0D2, ATXN10, CADM2, CCNE1, CD3E, CD3G, CDH10, CHMP2A, CHMP4A, CHRDL2, CLDN5, CNKSR1, CNRIP1, COPA, DDHD1, DDR1, DLG2, DOCK10, DPYD, EDA, EFNA1, EPB41L5, EXOC4, FADD, FCAR, FSCN2, FZD1, GALNT2, GCC1, GCHFR, GDNF, GNAQ, GPSM1, GRM8, HAS2, HAS3, IGSF9B, IKBKE, IPO4, ITGB5, KRT7, LGR4, LMF1, MAD1L1, MAST4, MGA, mir-383, NCOA3, NLGN4X, P2RX2, PDE4D, PDE5A, PKDCC, PLIN2, PLXDC2, PNPLA2, PPFIA4, PTCH1, RASGEF1B, RBFOX1, RBL2, RPLP2, RSL1D1, SEC23IP, SERPINA1, SERPINF1, SLC16A5, SLC22A23, TANC2, TEAD4, TENM3, THBS2, THRB, TIAL1, TIAM1, TJP2, TMED2, TNFRSF4, TNMD, TNR, TRHR, TRPS1, TWF1, UMPS, UTRN, XRN1*

TMED10	4.30×10^{-03}	3	75	*ABHD2, ABR, ADCY5, ADGRV1, ANKRD17, ARSB, ATP13A5, ATP6V0A2, CAMK2D, CCNE1, CCT6A, CD3E, CHRDL2, CNRIP1, CRACR2B, DDR1, DPYD, EDA, EDIL3, EIF2B1, FCAR, GDNF, HAS3, HID1, HMG20B, HPSE, IKBKE, IRAK4, KRT7, LFNG, MAD1L1, MED13L, mir-383, MZF1, NAV2, NCOA3, NCR1, NEDD8, P2RX2, PADI2, PCLO, PDE4D, PDE5A, PHF21A, PIDD1, PIK3CB, PIP5K1C, PLXDC2, PSPH, PTCH1, PYGB, RBFOX1, RGS7, ROBO1, RPLP2, SEMA3E, SGCZ, SHROOM3, SLC16A5, SLC25A4, ST8SIA1, STEAP3, SUCLG2, THRB, THSD4, TIAM1, TJP2, TMED2, TNFRSF18, TNFRSF4, TNN, TNR, TRHR, UMPS, VAV3*
TNFRSF10D	5.40×10^{-03}	3	87	*ABR, ADCY5, ADGRV1, ANKRD17, ANO1, ATP13A5, ATP6V0A2, CAMK2D, CCNE1, CCT6A, CD3G, CHMP4A, CHRDL2, CNKSR1, CNRIP1, COPA, CRACR2B, DDR1, DLG2, DPM3, DPYD, EDA, EDIL3, EFNA1, EIF2B1, EPB41L5, FCAR, GALNT2, GCHFR, GDNF, GNAQ, HAS2, HAS3, HERC3, HID1, HPSE, IKBKE, IRAK4, LFNG, LGR4, MAD1L1, MAST4, MED13L, mir-383, NCOA3, NCR1, NEDD8, NELL2, NLGN4X, OSBPL3, P2RX2, PADI2, PCLO, PDE4D, PHF21A, PIDD1, PIK3CB, PIK3R1, PLXDC2, POU2F2, PPFIA4, PSPH, PTCH1, PYGB, RBFOX1, RGS7, RMDN3, ROBO1, RPLP2, RSL1D1, SEC23IP, SGCZ, SHROOM3, SLC16A5, SLC25A4, ST8SIA1, STEAP3, THRB, THSD4, TIAM1, TJP2, TNMD, TNN, TNR, TRHR, UMPS, XRN1*
TNFRSF14	5.80×10^{-03}	3	83	*ABHD2, ABR, ADCY5, ANKRD17, AREG, ARSB, ATP13A5, ATP6V0A2, CAMK2D, CARD9, CCNE1, CD3D, CD3G, CHRDL2, CNKSR1, CNRIP1, CRACR2B, DDR1, DPM3, DPYD, EDA, EDIL3, EFNA1, EIF2B1, FCAR, FOXM1, GALNT2, GCHFR, GDNF, GNAQ, GPSM1, HAS2, HERC3, HID1, IKBKE, IRAK4, LGR4, MAST4, MED13L, mir-383, NAV2, NCOA3, NCR1, NEDD8, P2RX2, PADI2, PCLO, PDE4D, PHF21A, PIDD1, PIK3CB, PIP5K1C, PLXDC2, POU2F2, PPFIA4, PSPH, PYGB, RBFOX1, RGS7, RMDN3, ROBO1, RPLP2, SEC23A, SERPINA1, SERPINF2, SGCZ, SHROOM3, SLC16A5, SLC25A4, ST8SIA1, STEAP3, THRB, THSD4, TIAL1, TJP2, TMED2, TNFRSF18, TNMD, TNN, TNR, TRHR, UMPS, VAV3*
TNFRSF21	5.70×10^{-03}	3	74	*ABHD2, ABR, ADCY5, ADGRV1, ANKRD17, ATP6V0A2, ATXN10, CCNE1, CD3E, CDH10, CHRDL2, CNRIP1, CRACR2B, DDR1, DPYD, EDA, EDIL3, EIF2B1, FCAR, FZD1, GDNF, HAS3, HID1, HPSE, IKBKE, IRAK4, KRT7, LFNG, LGR4, MAD1L1, MAST4, MED13L, mir-*

				383, MZF1, NAV2, NCOA3, NEDD8, NELL2, P2RX2, PADI2, PCLO, PDE4D, PHF21A, PIDD1, PIK3CB, PIP5K1C, PLXDC2, PSPH, PTPRN2, PYGB, RBFOX1, RGS7, ROBO1, RPLP2, SEC23A, SEMA3E, SGCZ, SHROOM3, SLC16A5, SLC25A4, ST8SIA1, STEAP3, SUCLG2, THRB, THSD4, TIAM1, TMED2, TNFRSF18, TNFRSF4, TNN, TNR, TRHR, UMPS, VAV3
TNFSF18	8.60×10^{-03}	3	82	*ABR, ADCY5, ADGRV1, ANKRD17, ARSB, ATP13A5, ATP6V0A2, CAMK2D, CARD9, CCNE1, CD3D, CD3G, CHRDL2, CNKSR1, CNRIP1, CRACR2B, DDR1, DPYD, EDA, EDIL3, EFNA1, EIF2B1, EPB41L5, FCAR, GALNT2, GCHFR, GDNF, GNAQ, GPSM1, HAS2, HAS3, HERC3, HID1, IKBKE, IRAK4, LFNG, LGR4, MAD1L1, MAST4, MED13L, mir-383, NCOA3, NCR1, NEDD8, P2RX2, PADI2, PCLO, PDE4D, PHF21A, PIDD1, PIK3CB, PIP5K1C, PLXDC2, POU2F2, PPFIA4, PSPH, PYGB, RBFOX1, RGS7, RMDN3, ROBO1, RPLP2, SEC23A, SERPINF2, SGCZ, SHROOM3, SLC16A5, SLC25A4, ST8SIA1, STEAP3, THRB, THSD4, TIAL1, TIAM1, TJP2, TMED2, TNFRSF18, TNMD, TNN, TNR, UMPS, VAV3*
TNIK	3.50×10^{-03}	3	84	*ABR, ADCY5, ADGRV1, ANKRD17, ANO1, ATP13A5, ATP6V0A2, CAMK2D, CCNE1, CCT6A, CD3E, CDC45, CHMP4A, CHRDL2, CNRIP1, COPA, CRACR2B, DDR1, DLG2, DOCK10, DPM3, DPYD, EDA, EDIL3, EIF2B1, FCAR, GCHFR, GDNF, GNAQ, HID1, HPSE, IRAK4, LFNG, LGR4, MAD1L1, MAST4, MED13L, mir-383, NCOA3, NCR1, NEDD8, NELL2, NLGN4X, OSBPL3, P2RX2, PADI2, PCLO, PDE4D, PHF21A, PIDD1, PIK3CB, PLIN2, PLXDC2, PPFIA4, PSPH, PTCH1, PYGB, RASGEF1B, RBFOX1, RGS7, ROBO1, RPLP2, RSL1D1, SEC23IP, SEMA3E, SERPINF2, SGCZ, SHROOM3, SLC16A5, SLC22A23, SLC25A4, ST8SIA1, STEAP3, THRB, THSD4, TIAM1, TJP2, TNFRSF4, TNN, TNR, TRHR, TWF1, UMPS, XRN1*
TTK	7.60×10^{-03}	2	28	*ACTN1, AREG, CAMK2D, CCNE1, CDH10, CRACR2B, DDR1, EDIL3, EFNA1, EIF2B1, FOXM1, GBX2, HAS2, ITGB5, MAD1L1, MED13L, NEDD8, PADI2, PFKP, PIDD1, PSPH, PTCH1, ROBO1, SHROOM3, STEAP3, THBS2, TNFRSF18, UMPS*
UBA3	9.30×10^{-03}	3	90	*ABHD2, ABLIM1, ABR, ADCY5, ADGRV1, ANKRD17, AREG, ATP13A5, ATP6V0A2, CCNE1, CCT6A, CD3D, CD3G, CHMP4A, CHRDL2, CNRIP1, COPA, CRACR2B, DLG2, DPM3, DPYD, EIF2B1, FCAR, GALNT2, GCHFR, GDNF, GNAQ, HAS2, HAS3, HERC3, HID1,*

				HMG20B, IGSF9B, KRT7, LFNG, LGR4, MAST4, MED13L, mir-383, NAV2, NCOA3, NCR1, NEDD8, NELL2, NLGN4X, P2RX2, PADI2, PDE5A, PFKP, PHF21A, PIDD1, PIK3CB, PIP5K1C, PLXDC2, POU2F2, PPFIA4, PSPH, PTCH1, PYGB, RASGEF1B, RBFOX1, RBL2, RGS7, RMDN3, ROBO1, RPLP2, RSL1D1, SEC23A, SEC23IP, SERPINA1, SGCZ, SHROOM3, SLC16A5, SLC25A4, ST8SIA1, STEAP3, TANC2, TEAD4, TENM3, THRB, TIAM1, TJP2, TMED2, TNFRSF18, TNMD, TNN, TNR, TRPS1, VAV3, XRN1
UBE2E1	2.30×10^{-03}	3	55	*ABHD2, ABLIM1, ACTN1, ATP6V0D2, CAMK2D, CCNE1, CCT6A, CDC45, CDH10, CHMP4A, COPA, CRACR2B, DDR1, DLG2, EDIL3, EIF2B1, FADD, FZD1, GBX2, GNAQ, HMG20B, HPSE, KRT7, LGR4, LRP6, MAD1L1, MED13L, MZF1, NCOA3, NEDD8, NELL2, NLGN4X, PADI2, PFKP, PHF21A, PIDD1, PIK3R1, PLIN2, PNPLA2, RBL2, ROBO1, SEC23A, SEC23IP, SERPINF1, SHROOM3, SOX8, STEAP3, SUCLG2, THRB, TJP2, TRHR, UMPS, UTRN, VAV3, XRN1*
UBE2M	5.40×10^{-03}	3	74	*ABHD2, ABLIM1, AREG, ATP13A5, ATP6V0D2, CAMK2D, CCT6A, CD3D, CD3E, CDC45, CHMP4A, CNKSR1, COPA, CRACR2B, DDR1, DLG2, EDA, EFNA1, EIF2B1, FADD, FOXM1, GBX2, GNAQ, HAS3, HERC3, HMG20B, IGSF9B, ITGB5, KRT7, LGR4, LRP6, MAST4, MED13L, NAV2, NCOA3, NEDD8, NELL2, NLGN4X, PADI2, PDE5A, PFKP, PIDD1, PIK3CB, PIK3R1, PLXDC2, POU2F2, PTCH1, RASGEF1B, RBFOX1, RBL2, RMDN3, ROBO1, RSL1D1, SEC23IP, SEMA3E, SERPINA1, SERPINF2, SHROOM3, SLC25A4, STEAP3, TANC2, TEAD4, TENM3, THRB, THSD4, TIAM1, TJP2, TNFRSF18, TNFRSF4, TNN, TRPS1, UTRN, VAV3, XRN1*
UBE2M	8.10×10^{-03}	2	27	*ABHD2, ABLIM1, AREG, CCT6A, CHMP4A, COPA, DLG2, EFNA1, FOXM1, GNAQ, HMG20B, KRT7, LGR4, NCOA3, NELL2, NLGN4X, POU2F2, PTCH1, RBL2, ROBO1, SEC23IP, SLC25A4, THRB, TJP2, TNFRSF18, VAV3, XRN1*
UBE2O	2.80×10^{-03}	3	34	*ACTN1, AREG, CAMK2D, CCNE1, CDH10, CRACR2B, DDR1, EDIL3, EFNA1, EIF2B1, FOXM1, GBX2, HAS2, ITGB5, LRP6, MAD1L1, MED13L, NEDD8, PADI2, PFKP, PIDD1, PIK3CB, PTCH1, RBL2, ROBO1, SERPINA1, SHROOM3, SLC25A4, SMC1B, STEAP3, THBS2, TNFRSF18, TNN, UMPS*
UBE4B	1.70×10^{-03}	2	32	*ACTN1, AREG, CAMK2D, CCNE1, CDH10, CRACR2B, DDR1, EDIL3, EFNA1, EIF2B1, FOXM1, GBX2, HAS2, HAS3, IKBKE, ITGB5, KRT7,*

				MAD1L1, MED13L, NEDD8, PADI2, PFKP, PIDD1, PTCH1, RBL2, ROBO1, SERPINF1, SHROOM3, STEAP3, THBS2, TNFRSF18, UMPS
ubiquinone 9	8.50×10^{-03}	3	81	*ABHD2, ABR, ADCY5, ADGRV1, ANKRD17, ATP6V0A2, CAMK2D, CCNE1, CD3G, CDC45, CDH10, CHRDL2, CNKSR1, CNRIP1, CRACR2B, DDR1, DPM3, DPYD, EDA, EDIL3, EFNA1, EIF2B1, EPB41L5, FCAR, GALNT2, GCHFR, GDNF, HAS2, HAS3, HERC3, HID1, HPSE, IKBKE, IRAK4, LFNG, LGR4, MAD1L1, MAST4, MED13L, mir-383, NAV2, NCOA3, NCR1, NEDD8, P2RX2, PADI2, PCLO, PDE4D, PHF21A, PIDD1, PIK3CB, PIK3R1, PIP5K1C, PLXDC2, POU2F2, PPFIA4, PSPH, PYGB, RGS7, RMDN3, ROBO1, RPLP2, SEC23A, SERPINF2, SGCZ, SHROOM3, SLC16A5, SLC25A4, ST8SIA1, STEAP3, THRB, THSD4, TIAM1, TMED2, TNFRSF18, TNMD, TNN, TNR, TRHR, UMPS, VAV3*
USP9X	7.10×10^{-03}	1	2	*GEMIN2, GPSM1*
VLDL	1.50×10^{-03}	3	78	*ABLIM1, ABR, ADCY5, ARSB, ATP13A5, ATP6V0A2, CAMK2D, CCNE1, CCT6A, CD3E, CDC45, CHMP2A, CHMP4A, CNKSR1, CNRIP1, COPA, CRACR2B, DDR1, DLG2, DPYD, EDIL3, EIF2B1, FCAR, FOXM1, FZD1, GCHFR, GDNF, GNAQ, HERC3, HID1, HMG20B, IRAK4, MAD1L1, MED13L, mir-383, MZF1, NAV2, NCR1, NEDD8, NLGN4X, OSBPL3, P2RX2, PADI2, PDE4D, PDE5A, PHF21A, PIDD1, PIK3R1, PIP5K1C, PKDCC, PLIN2, PLXDC2, PPFIA4, PSPH, RBFOX1, RGS7, RPLP2, SEC23A, SEC23IP, SEMA3E, SGCZ, SHROOM3, SLC16A5, STEAP3, SUCLG2, THBS2, THSD4, TIAL1, TIAM1, TJP2, TMED2, TNN, TNR, TRHR, UMPS, UTRN, VAV3, XRN1*
WIPF1	1.40×10^{-03}	3	79	*ABHD2, ABR, ADCY5, ADGRV1, ANKRD17, ARSB, ATP6V0A2, CAMK2D, CARD9, CCNE1, CD3D, CD3E, CD3G, CDC45, CDH10, CHRDL2, CNRIP1, CRACR2B, DDR1, DPYD, EDA, EDIL3, EIF2B1, FCAR, GDNF, GNAQ, GPSM1, HERC3, HID1, HPSE, IKBKE, IRAK4, KRT7, LFNG, LGR4, MED13L, mir-383, NAV2, NCOA3, NCR1, NEDD8, OSBPL3, P2RX2, PADI2, PCLO, PDE4D, PHF21A, PIDD1, PIK3CB, PIK3R1, PIP5K1C, PLXDC2, PSPH, PTCH1, PYGB, RGS7, RMDN3, ROBO1, RPLP2, SEC23A, SEMA3E, SGCZ, SHROOM3, SLC16A5, SLC25A4, ST8SIA1, STEAP3, THRB, THSD4, TIAL1,*

				TIAM1, TMED2, TNFRSF18, TNFRSF4, TNN, TNR, TRHR, UMPS, VAV3
WRN	7.60×10^{-03}	2	30	*ACTN1, AREG, CAMK2D, CDH10, CRACR2B, DDR1, EDIL3, EFNA1, EIF2B1, FOXM1, FZD1, GBX2, HAS2, ITGB5, MAD1L1, MAST4, MED13L, NAV2, NEDD8, PADI2, PFKP, PIDD1, PTCH1, RBL2, ROBO1, SHROOM3, STEAP3, THBS2, TNFRSF18, UMPS*
YWHAQ	8.80×10^{-03}	3	66	*ABHD2, ABR, ADCY5, ARSB, ATP13A5, ATP6V0D2, CCT6A, CD3D, CD3E, CDC45, CDH10, CNKSR1, CRACR2B, DDR1, DOCK10, EDIL3, EIF2B1, FADD, FCAR, GBX2, GCHFR, HERC3, HID1, HMG20B, IGSF9B, IRAK4, LGR4, LRP6, MAD1L1, MED13L, MZF1, NCOA3, NEDD8, P2RX2, PADI2, PCLO, PIDD1, PIK3CB, PITRM1, PLXDC2, PNPLA2, PSPH, PYGB, RASGEF1B, RBFOX1, RBL2, RGS10, RGS7, RMDN3, RSL1D1, SEC23A, SEMA3E, SERPINF2, SHROOM3, SLC16A5, STEAP3, SUCLG2, TEAD4, TIAM1, TNFRSF4, TNN, TNR, TRHR, TRPS1, UMPS, VAV3*
ZBTB16	7.30×10^{-03}	2	6	*CD3D, CD3E, CD3G, KCNJ8, SERPINF1, TNFRSF4*
Z-IETD-FMK	2.10×10^{-03}	3	59	*ACTN1, AREG, ATXN10, CARD9, CD3D, CD3G, CDH10, CLDN5, CRACR2B, DDR1, DOCK10, EDIL3, EFNA1, EIF2B1, FADD, GBX2, GDNF, GNAQ, GPSM1, HAS2, HERC3, IKBKE, LFNG, LRP6, MAD1L1, MAST4, MED13L, MZF1, NAV2, NCOA3, NEDD8, NELL2, PADI2, PIDD1, PLXDC2, POU2F2, PTPRN2, RBFOX1, RGS7, RMDN3, ROBO1, SEC23A, SEMA3E, SERPINF1, SERPINF2, SHROOM3, SLC25A4, STEAP3, SUCLG2, THBS2, TIAL1, TIAM1, TNFRSF18, TNFRSF4, TNMD, TNN, TRHR, UMPS, UTRN*
ZNF274	8.30×10^{-03}	3	81	*ABHD2, ABR, ADCY5, ADGRV1, ANKRD17, ATP6V0A2, CAMK2D, CCNE1, CD3G, CDC45, CDH10, CHRDL2, CNKSR1, CNRIP1, CRACR2B, DDR1, DPM3, DPYD, EDA, EDIL3, EFNA1, EIF2B1, EPB41L5, FCAR, GALNT2, GCHFR, GDNF, HAS2, HAS3, HERC3, HID1, HPSE, IKBKE, IRAK4, LFNG, LGR4, MAD1L1, MAST4, MED13L, mir-383, NAV2, NCOA3, NCR1, NEDD8, P2RX2, PADI2, PCLO, PDE4D, PHF21A, PIDD1, PIK3CB, PIK3R1, PIP5K1C, PLXDC2, POU2F2, PPFIA4, PSPH, PYGB, RGS7, RMDN3, ROBO1, RPLP2, SEC23A, SERPINF2, SGCZ, SHROOM3, SLC16A5, SLC25A4, ST8SIA1, STEAP3, THRB, THSD4, TIAM1, TMED2, TNFRSF18, TNMD, TNN, TNR, TRHR, UMPS, VAV3*

ZNF346	7.60 × 10^{-03}	2	28	*ACTN1, AREG, CAMK2D, CCNE1, CDH10, CRACR2B, DDR1, EDIL3, EFNA1, EIF2B1, FOXM1, GBX2, HAS2, ITGB5, MAD1L1, MED13L, NEDD8, PADI2, PFKP, PIDD1, PTCH1, RBL2, ROBO1, SHROOM3, STEAP3, THBS2, TNFRSF18, UMPS*
ZNF675	8.30 × 10^{-03}	3	81	*ABHD2, ABR, ADCY5, ADGRV1, ANKRD17, ATP6V0A2, CAMK2D, CCNE1, CD3G, CDC45, CDH10, CHRDL2, CNKSR1, CNRIP1, CRACR2B, DDR1, DPM3, DPYD, EDA, EDIL3, EFNA1, EIF2B1, EPB41L5, FCAR, GALNT2, GCHFR, GDNF, HAS2, HAS3, HERC3, HID1, HPSE, IKBKE, IRAK4, LFNG, LGR4, MAD1L1, MAST4, MED13L, mir-383, NAV2, NCOA3, NCR1, NEDD8, P2RX2, PADI2, PCLO, PDE4D, PHF21A, PIDD1, PIK3CB, PIK3R1, PIP5K1C, PLXDC2, POU2F2, PPFIA4, PSPH, PYGB, RGS7, RMDN3, ROBO1, RPLP2, SEC23A, SERPINF2, SGCZ, SHROOM3, SLC16A5, SLC25A4, ST8SIA1, STEAP3, THRB, THSD4, TIAM1, TMED2, TNFRSF18, TNMD, TNN, TNR, TRHR, UMPS, VAV3*

[1]This is the network bias corrected p-value, which accounts for network bias due to the presence of hub genes in the curated datasets.

[2]Depth scores represent the degree of separation or number of intermediate molecules (2 or 3) between the master regulator and the positional candidate genes.

[3]This is the number of positional candidate genes controlled by the master regulator.

Table S4. Master regulators associated with top positional candidate genes.

<table>
<tr><th>Positional Candidate Gene</th><th>Unique Master Regulators</th><th>Shared Master Regulators</th></tr>
<tr><td>MIR-383</td><td>CMA1, DNMT1, IL13RA2, NPY, PRKD2, SPSB1, THPO</td><td rowspan="2">Act1, AKR1B1, Aldose Reductase, ARHGAP1, BAK1, CARD11, CD180, CERS2, CRTC1, DAXX, DR4/5, DYNLL1, E2F3, EDAR, ERN1, Filamin, FLNB, FZD5, GCKR, Hspa1b, IL1RAP, imidazoquinolines, KLC1, KRT18, KRT8, KSR2, LGALS7/LGALS7B, mannan, MKK1/4, Nr1h, PDCD6, PELI2, PFKFB3, phosphatidylcholine, PIN1, PLA2G2A, PLC, PNPLA8, RANBP2, RPS3, S100B, SFRP2, SIAH1, SIAH2, SIGIRR, Sox, SPATA2, TFIIH, TMED10, TNFRSF10D, TNFRSF14, TNFRSF21, TNFSF18, TNIK, UBA3, ubiquinone 9, VLDL, WIPF1, ZNF274, ZNF675</td></tr>
<tr><td>SGCZ</td><td>ABL1, BTK, cytokine receptor, DHCR24, MAP3K3, SLK</td></tr>
</table>
